# Sorbicillinoid-Based Metabolites from a Sponge-Derived Fungus *Trichoderma saturnisporum*

**DOI:** 10.3390/md16070226

**Published:** 2018-07-04

**Authors:** Junjun Meng, Wei Cheng, Hajar Heydari, Bin Wang, Kui Zhu, Belma Konuklugil, Wenhan Lin

**Affiliations:** 1 State Key Laboratory of Natural and Biomimetic Drugs, Peking University, Beijing 100191, China; 13683042952@163.com (J.M.); chengwei@bjmu.edu.cn (W.C.); 2 Food and Medicine College, Zhejiang Ocean University, Zhoushan 316022, China; wangbin@zjou.edu.cn; 3 Department of Pharmacoguosy, Faculty of Pharmacy, Ankara University, Tandogan, Ankara 06100, Turkiye; hajar.heydari@yahoo.com (H.H.); belma.konuklugil@gmail.com (B.K.); 4 College of Veterinary Medicine, China Agricultural University, Beijing 100193, China; zhukcau@gmail.com

**Keywords:** marine fungus, *Trichoderma saturnisporum*, saturnispols A–H, structural elucidation, antibacterial effects

## Abstract

Antibacterial activity assessment and high performance liquid chromatography associated with nuclear magnetic resonance (HPLC/NMR) data revealed that the EtOAc extract of the fermented endophytic fungus *Trichoderma saturnisporum* DI-IA, obtained from the marine sponge *Dictyonella incisa*, contained conjugated olefinic metabolites with antibacterial activity. Chemical examination of the fungal strain resulted in the isolation of eight new sorbicillinoid-based compounds, namely saturnispols A–H (**1**–**8**). Their structures were determined on the basis of extensive spectroscopic analysis, including the experimental and calculated electronic circular dichroism (ECD) data for the configurational assignments. Saturnispol F exerted significant inhibition against a panel of bacteria strains including vancomycin-resistant enterococci (VRE) with a minimum inhibitory concentrations (MIC) ranging from 1.63 to 12.9 μg/mL, while saturnispol H showed selective effects against VRE and *B. subtilis*.

## 1. Introduction

Sorbicillinoids are a class of fungal metabolites with a cyclic hexaketide nucleus, which contains a typical or modified sorbyl sidechain [1,2]. Since the isolation of the first member of the sorbicillinoid family 60 years ago (sorbicillin, from the fungus *Penicillium notatum*) [3], approximately 100 sorbicillin derivatives have been reported from several strains of fungi obtained from diverse environmental sources. Based on their structural modification and condensation, they are classified into monomers [4], bisorbicillinoids [5,6,7,8], trisorbicillinoids [9,10], and hybrid sorbicillinoids [11,12,13]. These typical natural products possess a range of bioactivities, including cytotoxic [14], antibiotic [15], antiviral [16], antioxidant [17], and lipid-lowering properties [18]. However, few studies have been reported that investigate the structure-activity relationship. Although most sorbicillinoids have originated from terrestrial fungi, marine-derived fungi have proved to be a promising source of derivatives. It is noted that sorbicillinoid-bearing marine fungi are distributed widely, from shallow water to deep sea environments. Among the marine-derived analogues, marine sponge-associated fungi are recognized to produce diverse sorbicillinoid derivatives with unique scaffolds. Sorbifuranones A–C from the sponge-derived fungus *Penicillium chrysogenum* are a novel class of intriguing sorbicillinol-derived metabolites [19]. JBIR-59, a dimer of sorbicillinol that lacks a sorbyl sidechain, has antioxidant effects and is produced by the sponge-derived fungus *P. citrinum* [17], while a group of novel sorbicillinoid polyketide derivatives were isolated from the Caribbean sponge *Agelas dispar*-associated fungus *Trichoderma* sp. [20]. Sorbicillinoid alkaloids were first found from a sponge-derived *P. chrysogenum* strain [16], and vertinoid polyketides with unique scaffolds were isolated from the sponge-associated fungus *T. longibrachiatum*. These findings demonstrate that sponge-associated fungi are a rich source of structurally unique sorbifuranones.

**Figure 1:**
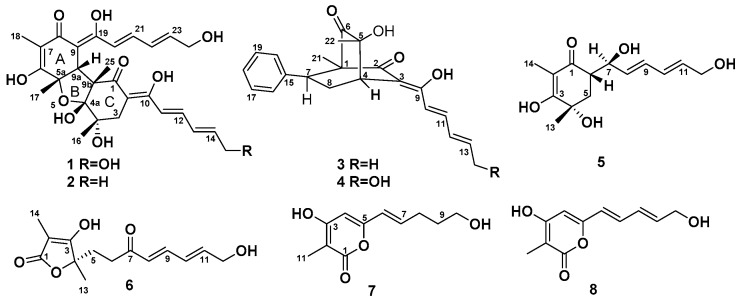
Structures of saturnispols A–H (**1**–**8**).

Vancomycin-resistant enterococci (VRE) have emerged as important hospital-acquired pathogens in immunosuppressed patients and are increasing steadily in occurrence. Antimicrobial therapy is problematic for all VRE, posing serious risks to the health of current and future patients. It is urgent to discover new natural antibiotics to overcome these drug-resistant pathogens. In the course of our search for antibacterial natural products derived from sponge-associated fungi, the marine fungus *Trichoderma saturnisporum*, which was isolated from the sponge of *Dictyonella incise*, showed a diverse chemical profile in its HPLC fingerprint and exerted antibacterial effects. This fungal species has rarely been chemically examined, with only one publication reporting peptaibols, namely paracelsins A–E [21]. Several *Trichoderma* species from terrestrial ecosystems have been registered as biocontrol agents to control fungal plant diseases [22,23]. The fungus *T. saturnisporum* is an unexploited species for the biocontrol of fungal diseases of plants, and it is a potential antagonist against important plant pathogens [22]. However, the chemical and biological properties of the marine-derived *T. saturnisporum* are unexplored and rarely described. In the present work, we describe the structural elucidation of eight new sorbicillinoid-based derivatives (Figure 1) from the sponge *Dictyonella incisa*-associated fungal strain *T. saturnisporum* and their antibacterial effects.

## 2. Results

The HPLC chromatograph in association with the ^1^H NMR and electrospray ionization mass spectrometry (ESI-MS) data revealed that the ethyl acetate (EtOAc) extract of the rice fermentation of the fungus *T. saturnisporum* featured a profile of conjugated olefinic metabolites. The EtOAc extract inhibited *Staphylococcus aureus* ATCC 29213 with an MIC < 128 μg/mL. Chromatographic separation of the EtOAc extract led to the isolation of eight new sorbicillinoid-related derivatives (Figure 1).

**Figure 2:**
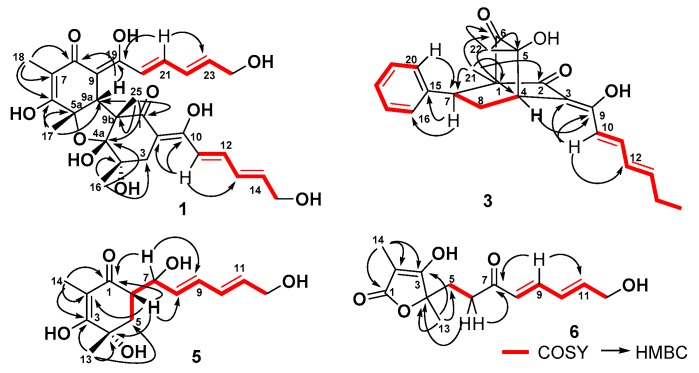
Key COSY and HMBC correlations of **1**, **3**, **5**, and **6**.

Saturnispol A (**1**) was obtained as a red oil. Its molecular formula was determined to be C_28_H_34_O_10_ on the basis of the high resolution electrospray ionization mass spectroscopy (HRESIMS) (*m*/*z* 529.2069 [M − H]^−^, calcd for C_28_H_33_O_10_, 529.2074) and NMR data, requiring 12 degrees of unsaturation. The infrared radiation (IR) absorptions suggested the presence of hydroxy (3380 cm^−1^), carbonyl (1738 cm^−1^), and olefinic (1614 cm^−1^) groups. The ^13^C NMR and distortionless enhancement by polarization transfer (DEPT) spectra exhibited a total of 28 carbon resonances, and they were classified into four methyls, three methylenes, nine methines, and 12 quaternary carbons. The connectivity of protons with their associated carbons was assigned by the heteronuclear single quantum coherence (HSQC) spectrum. The proton-proton correlation spectroscopy (^1^H–^1^H COSY) correlations established a side chain for a hydroxylated pentadiene from H-20 (*δ*_H_ 6.62, d, *J* = 15.2 Hz) to H_2_-24 (*δ*_H_ 4.22, d, *J* = 5.2 Hz). Inspection of the heteronuclear multiple bond correlation (HMBC) data, such as from H_3_-17 (*δ*_H_ 1.45, s) to C-6 (*δ*_C_ 168.3), C-5a (*δ*_C_ 79.2), and C-9a (*δ*_C_ 53.7), H_3_-18 (*δ*_H_ 1.43, s) to C-6, C-7 (*δ*_C_ 109.3), and C-8 (*δ*_C_ 191.9), as well as the correlations from H-9a (*δ*_H_ 3.67, s) to C-8, C-9 (*δ*_C_ 101.7), C-6, and C-5a, resulted in the presence of a cyclohexenone ring, in which Me-17 and Me-18 were positioned at C-5a and C-7, respectively. Additional HMBC correlations from H-20 to C-9 and C-19 (*δ*_C_ 166.5) deduced the location of a dihydroxylated hexadiene at C-9. Thus, the partial structure of compound **1** closely related to bisvertinol [9] was identified. Further examination of the HMBC and COSY correlations (Figure 2) established the second moiety for ring C to be a cyclohexanone unit, in which the same side chain as that of the first moiety was located at C-2 (*δ*_C_ 105.1) based on the HMBC correlations from H_2_-3 (*δ*_H_ 2.75, 2.47, d, *J* = 14.6 Hz) and H-11 (*δ*_H_ 6.42, d, *J* = 15.2 Hz) to C-2 and C-10 (*δ*_C_ 177.4). The coexistence of a methyl and a hydroxy group at C-4 (*δ*_C_ 72.8) was demonstrated by the HMBC correlations from H_3_-16 (*δ*_C_ 1.22, s) to C-3 (*δ*_C_ 35.1), C-4, and C-4a (*δ*_C_ 105.9), while the latter was assigned as a hemiketal carbon. Moreover, the HMBC correlations from H_3_-25 (*δ*_H_ 1.29) to C-1 (*δ*_C_ 193.6), C-9b (*δ*_C_ 59.2), and C-4a established that the methyl group and ketone were positioned at C-9a and C-1, respectively. Thus, the second moiety was determined to be related to that of bisvertinol. Since the assigned moieties required 11 degrees of unsaturation, the remaining one set was suggested to be contributed by a cyclic unit. The HMBC correlations between H_3_-25 and C-9a and from H-9a to C-4a and C-9a, in association with the deshielded shifts of C-4a and C-5a, allowed the formation of a tetrahydrofuran ring between rings A and C.

**Figure 3:**
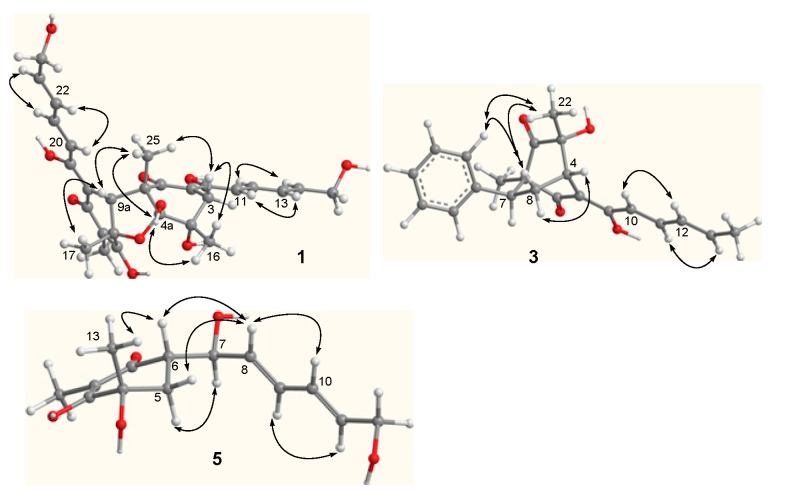
Key nuclear overhauser effect (NOE) interactions of **1**, **3**, and **5**.

**Figure 4:**
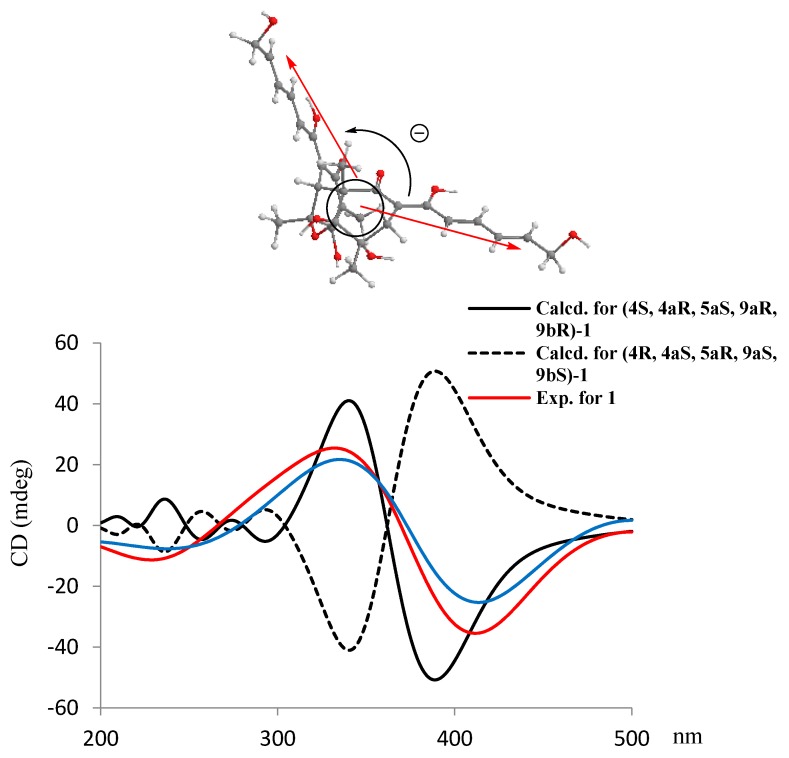
Experimental electronic circular dichroism (ECD) curves of **1** and **2** and the calculated ECD spectra of (±)-**1**.

The relative configuration of compound **1** was inferred from coupling constants and the NOE data. The conjugated double bonds at the side chains were assigned *E* geometries according to the large *J* values (15.2 Hz for *J*_H-11/H-12_, *J*_H-13/H-14_, *J*_H-22/H-23_, and *J*_H-20/H-21_). The *cis* fusion of rings A and B was ascribed to the NOE correlation between H_3_-17 and H-9a, while the *cis* fusion of rings B and C was suggested by the minimized energy for *cis* and *trans* junction of rings B and C, supporting more stability with lower energy for the *cis* fusion [23]. Additional NOE interactions from H_3_-25 to H-9a, H-3b (*δ*_H_ 2.47, d, *J* = 14.6 Hz) and between H-3b and H_3_-16 revealed H-9a and H_3_-16 to be cofacial with H_3_-25 (Figure 3). Based on the electronic circular dichroism (ECD) exciton chirality rule [24], the negative Cotton effect at 412 nm (∆*ε* −35.5), and a positive Cotton effect at 332 nm (∆*ε* +25.5) reflected the chromophores of two enol-sorbyl side chains to adopt a counterclockwise fashion (Figure 4), indicating 9b*R* configuration. Accordingly, the remaining stereogenic centers were assumed to be 4*S*, 4a*R*, 5a*S*, and 9a*R* configurations. Comparison of the experimental ECD data with that calculated for the enantiomers of **1** at the B31YP/6-311++G(2d, 2p) level in the gas phase using the B3LYP/6-31G(d) optimized geometries after conformational searches via the MMFF94S force field [25,26] (Figure 4) further supported the configurational assignment.

The molecular formula of saturnispol B (**2**) was determined as C_28_H_34_O_9_ on the basis of the HRESIMS (*m*/*z* 513.2125 [M − H]^−^, calcd for C_28_H_33_O_9_, 513.2125) and NMR data, showing the loss of one oxygen atom in comparison with that of compound **1**. Comparison of the NMR data (Table 1) and analyses of the two dimensional nuclear magnetic resonance (2D NMR) data revealed that the structure of compound **2** was closely related to that of compound **1**. The main difference was due to the presence of a methyl group (*δ*_H_ 1.87, d, *J* = 6.8 Hz; *δ*_C_ 18.1) at the side chain of ring C to replace the hydroxymethylene group of **1**. The COSY relationship between H_3_-15 (*δ*_H_ 1.87, d, *J* = 6.8 Hz) and H-14 (*δ*_H_ 6.24, dq, *J* = 6.8, 15.0 Hz) in association with the HMBC correlations from H_3_-15 to C-13 (*δ*_C_ 131.1) and C-14 (*δ*_C_ 139.2) supported the methyl location. The similar NOE interactions, such as from H-9a (*δ*_H_ 3.75) to H_3_-17 (*δ*_H_ 1.45) and H_3_-25 (*δ*_H_ 1.34) and from H-3b (*δ*_H_ 2.53, d, *J* = 14.0 Hz) to H_3_-25 and H_3_-16 (*δ*_H_ 1.19), identified the relative configuration of **2** to be the same as that of **1**. The similar Cotton effects in the ECD spectra (Figure 4) suggested **2** to be a 15-dehydroxylated analogue of **1**.

**Figure 5:**
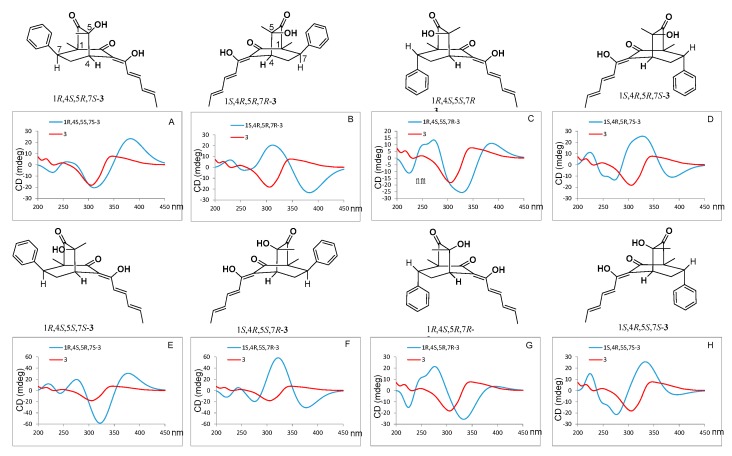
Experimental ECD of **3** and calculated ECD spectra of the isomers of **3**.

Saturnispol C (**3**) has a molecular formula of C_22_H_24_O_4_, as established by the HRESIMS (*m*/*z* 351.1600 [M − H]^−^, calcd for C_22_H_23_O_4_, 351.1596) and NMR data, containing 11 degrees of unsaturation. The IR absorptions at 3429, 1730, and 1601 cm^−1^ suggested the presence of hydroxy and ketone groups. The ^13^C NMR and DEPT spectra exhibited a total of 22 resonances for three methyls, one methylene, 11 methines, and seven quaternary carbons, of which 12 olefinic or aromatic resonances and two ketones were distinguished. A COSY spin system initiated from H-10 (*δ*_H_ 6.30, d, *J* = 14.8 Hz) and extended to H-14 (*δ*_H_ 1.94, d, *J* = 6.8 Hz), along with the HMBC correlation from H-10 and a D_2_O exchangeable proton at *δ*_H_ 14.34 (s) to C-9 (*δ*_C_ 167.1) and C-3 (*δ*_C_ 112.0), assigned an enolic sorbyl side chain. A cyclohexandione ring was inferred from the HMBC correlations from H_3_-21 (*δ*_H_ 0.93, s) to C-1 (*δ*_C_ 64.6) and two ketone carbons C-2 (*δ*_C_ 197.7) and C-6 (*δ*_C_ 211.7), H_3_-22 (*δ*_H_ 1.29, s) to C-4 (*δ*_C_ 40.5), C-5 (*δ*_C_ 74.8) and C-6, in addition to the correlation from H-4 (*δ*_H_ 3.32, t, *J* = 2.8 Hz) to C-2, C-3, C-5, and C-6 (Figure 2). These data also revealed the position of the methyl groups at C-1 and C-5 and an enolic sorbyl unit at C-3, respectively. The HMBC correlation from a D_2_O exchangeable proton at *δ*_H_ 2.69 (s) to C-4, C-5, and C-6 showed C-5 to be hydroxylated. Moreover, a mono-substituted phenyl ring was recognized by the spin system of the aromatic protons at *δ*_H_ 6.98 (d, *J* = 7.6 Hz, 2H) and 7.26 (t, *J* = 7.6 Hz, 3H). The remaining two alkyl carbons C-7 (*δ*_C_ 47.7) and C-8 (*δ*_C_ 31.4) formed a substituted ethyl unit according to the COSY relationship between H-7 (*δ*_H_ 3.15, dd, *J* = 5.6, 10.8 Hz) and H_2_-8 (*δ*_H_ 1.90, 3.05), while the linkage of the aromatic ring at C-7 was based on the HMBC correlation from H-7 to the aromatic carbons C-15 (*δ*_C_ 141.4) and C-16/C-20 (*δ*_C_ 128.4). Diagnostic COSY and HMBC data (Figure 2), such as the HMBC correlation between H_3_-21 and C-7 and the COSY relationship from H_2_-8 to H-4 and H-7, confirmed the fusion of an ethylbenzene unit to the cyclohexandione ring by the C-1/C-7 and C-4/C-8 linkage. Thus, the planar structure with a bicyclo (2,2,2) octane skeleton was established. The NOE interactions between H_3_-22/H-8b (*δ*_H_ 3.05), H-8b/H-16(H-20) (*δ*_H_ 6.98), and H-8b/ H-16(H-20) revealed Me-22 to be spatially approximated to H-8b, while both H-8b and the aromatic moiety were in the same face (Figure 3). In order to assign the absolute configuration, the ECD data of eight isomers with the same planar structure of compound **3** were calculated at the B31YP/6-311++G(2d, 2p) level. As shown in Figure 5, isomers (Figure 5A,C,E,G) with 1*R* and 4*S* configurations exhibited a negative Cotton effect (CE) at 300–340 nm and a positive CE at 350–400 nm, whereas the opposite phases were observed in the same range for the isomers with 1*S* and 4*R* configurations (Figure 5B,D,F,H) based on the calculated results. Thus, the negative CE at 310 nm and positive CE at 350 nm in the ECD spectrum of **3** were in agreement with 1*R* and 4*S* configurations. However, the configurational variation at C-5 and C-7 slightly induced the Cotton effects. Based on the NOE correlation mentioned above, the aromatic moiety linked to C-7 was oriented toward the hydroxy-ketone bridge and OH-5 was located on the same side as the unsaturated side chain, thus the stereogenic centers at C-5 and C-7 were assumed to be 5*R* and 7*S* configurations.

**Figure 6:**
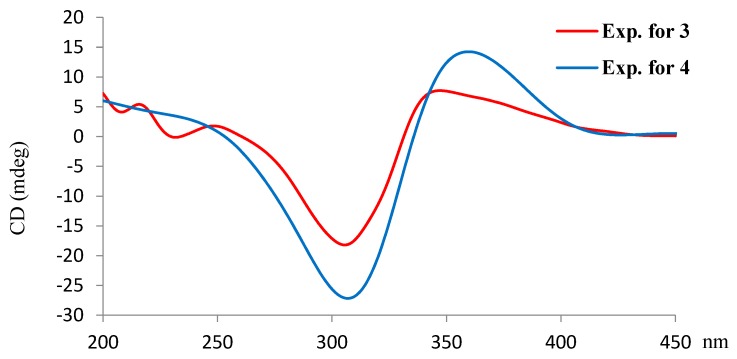
Experimental ECD curves of **3** and **4**.

The NMR data of saturnispol D (**4**) were almost superimposed to those of **3** (Table 2), with the exception of a hydroxymethylene unit in **4** to replace the terminal methyl group at the enolic sorbyl side chain. The COSY relationship between the hydroxymethylene protons H_2_-14 (*δ*_H_ 4.28, d, *J* = 4.4 Hz) and the olefinic proton H-13 (*δ*_H_ 6.39, dt, *J* = 4.4, 15.2 Hz) in addition to the HMBC correlations from H_2_-14 to C-12 (*δ*_C_ 127.7) and C-13 (*δ*_C_ 142.9) indicated **4** to be a 14-hydroxylated analogue of **3**. The comparable ECD data supported both **3** and **4** sharing the same configuration (Figure 6).

**Figure 7:**
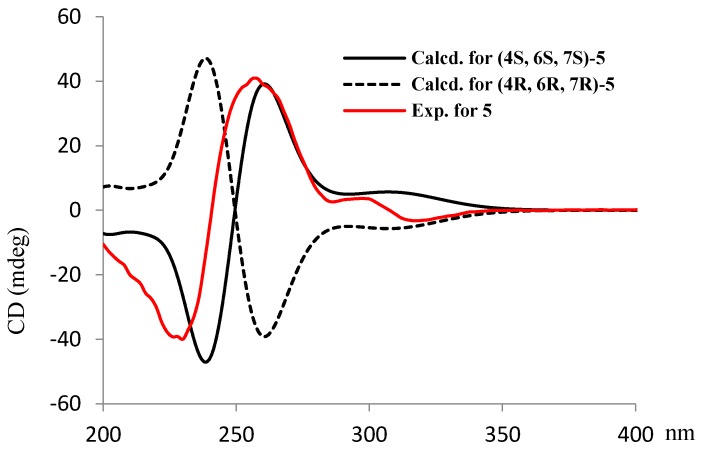
Experimental ECD curves of **5** and the calculated ECD spectra of (±)-**5**.

The molecular formula (C_14_H_20_O_5_) of saturnispol E (**5**) was determined by the HRESIMS (*m*/*z* 267.1237 [M − H]^−^, calcd for C_14_H_19_O_5_, 267.1232) and NMR data, bearing 5 degrees of unsaturation. IR absorptions at 3365, 1740, and 1623 cm^−1^ suggested the presence of hydroxy and carbonyl groups. The ^1^H and ^13^C NMR data (Table 3 and Table 4) suggested **5** to be structurally related to sorbicillinol [27]. A cyclohexenone nucleus was demonstrated by the COSY coupling between H-6 (*δ*_H_ 2.69, ddd, *J* = 4.8, 7.4, 12.0 Hz) and H_2_-5 (*δ*_H_ 1.66, 1.98), and the HMBC correlations from H_3_-13 (*δ*_H_ 1.34, s) to C-3 (*δ*_C_ 192.2), C-4 (*δ*_C_ 71.1), and C-5 (*δ*_C_ 37.1), H_3_-14 (*δ*_H_ 1.68, s) to C-1 (*δ*_C_ 183.2), C-2 (*δ*_C_ 108.4), and C-3, in association with the correlations from H-6 to C-1, C-2, C-4, and C-5. These findings also allowed the substitution of methyl groups at C-2 and C-4, respectively. The deshielded quaternary carbons C-3 and C-4, as in the case of **1**, suggested both C-3 and C-4 to be hydroxylated. The COSY relationships established a spin system for a linear side chain of dihydroxyhexadiene, in which a conjugated diene was located between C-8 (*δ*_C_ 131.6) and C-11 (*δ*_C_ 133.5). The COSY relationships between H-7 (*δ*_H_ 4.49, t, *J* = 7.4 Hz)/H-8 (*δ*_H_ 5.65, dd, *J* = 7.4, 14.4 Hz) and H-11 (*δ*_H_ 5.86, dt, *J* = 5.6, 14.4 Hz)/H_2_-12 (*δ*_H_ 4.12, d, *J* = 5.6 Hz), in addition to the carbon resonances of C-7 (*δ*_C_ 74.2) and C-12 (*δ*_C_ 61.7) as assigned by the HSQC spectrum, indicated both C-7 and C-12 to be hydroxylated. The linkage of the side chain to C-6 (*δ*_C_ 43.9) was evident from the COSY coupling between H-6 and H-7, as well as the HMBC correlation from H-7 to C-1, C-5 and C-6. The *E, E* geometry of the conjugated diene was deduced by the coupling constants of *J*_H-8/H-9_ and *J*_H-10/H-11_ (15.4 Hz). The NOE interaction between H-6 and H_3_-13 suggested the same orientation of both protons (Figure 3). The *J*_H-6/H-7_ (7.4 Hz) value, in addition to the NOE interaction from H-7 (*δ*_H_ 4.49) and H-8 to H-5a (*δ*_H_ 1.66), and that between H-6 and H-8, suggested that the side chain maintained a stable geometry, in which H-7 was *trans* relative to H-6, while H-7 and H-8 were spatially close to H-5a. Thus, the relative configurations of **5** were suggested to be 4S*, 6S*, and 7S*. The ECD data for the stereogenic isomers of (4*R*, 6*R*, 7*R*)-**5** and (4*S*, 6*S*, 7*S*)-**5** were calculated. Comparison of the experimental ECD data with those calculated for the isomers confirmed **5** to be in 4*S*, 6*S*, and 7*S* configurations (Figure 7).

**Figure 8:**
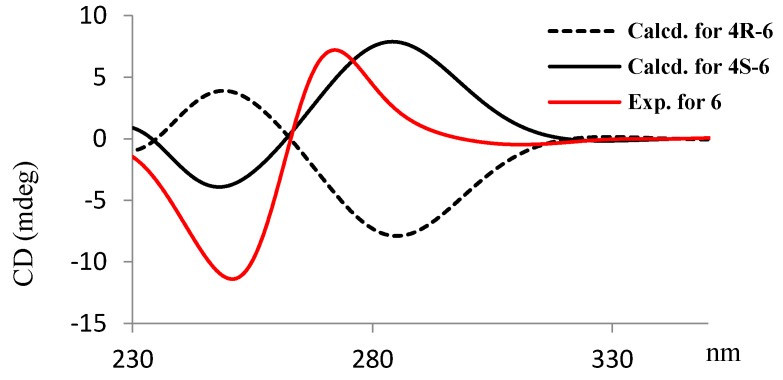
Experimental and calculated ECD spectra of **6**.

Saturnispol F (**6**) has a molecular formula of C_14_H_18_O_5_, as established by the HRESIMS (*m*/*z* 265.1073 [M − H]^−^, calcd for C_14_H_17_O_5_, 265.1076) and NMR data. The ^13^C NMR and DEPT spectra displayed 14 carbon resonances, including six olefinic carbons, two carbonyl carbons, and three methylene carbons. The IR absorptions at 1737 and 1655 cm^−1^ suggested the presence of an unsaturated lactone. The ^1^H NMR spectrum exhibited the proton resonances, including two methyl singlets at *δ*_H_ 1.44 (s, H_3_-13) and *δ*_H_ 1.66 (s, H_3_-14). The ^1^H-^1^H COSY spectrum correlated four conjugated olefinic protons for a diene unit from H-8 (*δ*_H_ 6.18, d, *J* = 16.0 Hz) to H-11 (*δ*_H_ 6.36, dt, *J* = 4.6, 16.0 Hz) and the protons for two methylenes H_2_-5 (*δ*_H_ 2.07, t, *J* = 7.6 Hz) and H_2_-6 (*δ*_H_ 2.50, t, *J* = 7.6 Hz), while H-11 extended its COSY correlation with the methylene protons at *δ*_H_ 4.22 (d, *J* = 4.6 Hz). These data are in agreement with the HMBC correlations from a ketone carbon (*δ*_C_ 200.6, C-7) to H_2_-5, H_2_-6, H-8, and H-9 (*δ*_H_ 7.25, dd, *J* = 10.6, 16.0 Hz), which established a 11-hydroxy-7-oxo-8,10-octadienyl side chain. The remaining NMR resonances were attributed to C-1 (*δ*_C_ 176.8), C-2 (*δ*_C_ 93.6), C-3 (*δ*_C_ 180.8), C-4 (*δ*_C_ 83.0), and two methyl groups. The HMBC spectrum correlated H_3_-13 with C-3 and C-4 and H_3_-14 with C-1, C-2, and C-3, in association with the degrees of the molecular unsaturation, allowed the assignment of a 2,4-dimethyl-*γ*-lactone unit, while the remaining OH group was positioned at the quaternary carbon C-3. The linkage of the side chain to C-4 in the *γ*-lactone ring was evident from additional HMC correlations from H_3_-13 to C-5 (*δ*_C_ 30.6) and H_2_-5 to C-3 and C-4. Thus, the planar structure of **6** was closely related to vertinolide [28], except for the substitution of a hydroxy group at C-12 (*δ*_C_ 61.4). The *E, E* geometry of the diene was evidenced by the coupling constants of *J*_H-8/H-9_ and *J*_H-10/H-11_ (16.0 Hz). Regarding the absolute configuration, the comparable specific rotation between **6** (${\left[\alpha \right]}_{D}^{15}$, CHCl_3_) and (*S*)-(-)-vertinolide [29] suggested the 4*S* configuration of **6**. This depiction was confirmed by the experimental ECD data, which agreed with those calculated for the 4*S* isomer (Figure 8).

The HRESIMS data provided the molecular formula of saturnispol G (**7**) as C_11_H_14_O_4_, requiring 5 degrees of unsaturation. The HSQC spectrum assigned all protons and their corresponding carbons. The ^1^H NMR spectrum showed three olefinic protons, a methyl singlet, and six alkyl protons, while the ^13^C NMR and DEPT spectra afforded a methyl, three methylenes, six olefinic resonances, and a carbonyl carbon. Analyses of the HMBC data, such as the correlations from H_3_-11 (*δ*_H_ 1.89, s) to C-1 (*δ*_C_ 164.4), C-2 (*δ*_C_ 98.9), and C-3 (*δ*_C_ 156.1) and from H-4 (*δ*_H_ 6.01, s) to C-2 and C-5 (*δ*_C_ 164.8), suggested a 2-methyl-3-hydroxypyranone nucleus. The COSY relationships provided a spin system for a hydroxylated pentene side chain, in which the hydroxy group was positioned at C-10 (*δ*_C_ 60.7), based on the COSY relationship between H_2_-10 (*δ*_H_ 3.61, t, *J* = 6.4 Hz) and H_2_-9 (*δ*_H_ 1.71, tt, *J* = 6.4, 7.6 Hz). The HMBC correlation of the olefinic proton H-6 (*δ*_H_ 6.11, d, *J* = 16.0 Hz) to C-4 (*δ*_C_ 99.6) and C-5 (*δ*_C_ 164.8) allowed the side chain to be linked to C-5. The 6*E* geometry was identified by the *J*_H-6/H-7_ (16.0 Hz) value.

The structure of saturnispol H (**8**) was determined as a 6,8-diene analogue of **7**, based on the similar NMR data of both compounds (Table 3 and Table 4) with the exception of an additional double bond C-8 (*δ*_C_ 128.4)/C-9 (*δ*_C_ 138.4). The COSY correlation established a spin system of a side chain from H-6 to H_2_-10 (*δ*_H_ 4.20, d, *J* = 5.0 Hz). The geometry of the diene unit was in 6*E*, 8*E* according to the *J* values.

**Scheme 1:**
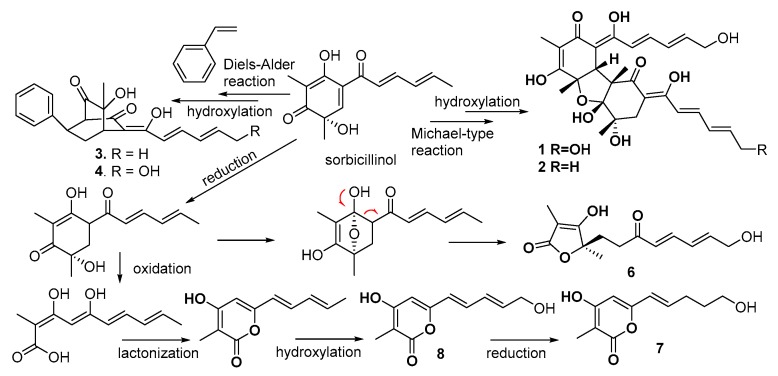
Postulation of the biogenetic relationship among the isolated compounds.

Biogenetically, sorbicillin-derived sorbicillinol is postulated as the precursor to generate the isolated compounds. The bisorbicillinoids **1** and **2** were supposed to be derived by the condensation of two sorbicillinol units via a Michael-type condensation, an approach similar to the biogenetic formation of bisvertinolone [30]. The hydroxylation at the terminal of the side chain was likely induced by the P450 enzyme. The (2 + 4) “Diels-Alder” cycloaddition [31] of sorbicillinol with a phenylethylene generated **3**, followed by hydroxylation to yield **4**. Compound **5** was likely generated from sorbicillinol by olefinic reduction and enol rearrangement. The unsaturated *γ*-lactone was supposed to be originated from sorbicillinol through reduction, hemiketal formation, *retro*-Claisen cleavage [32], and terminal hydroxylation. Neumann [33] postulated that the pyrone unit was derived from sorbicillinol by multiple steps. We suggest oxidation to lose a C_3_ unit in sorbicillinol to produce **8** and **7** (Scheme 1).

All compounds were evaluated for antimicrobial activity toward several bacterial strains, including *Staphylococcus aureus* ATCC 29213, vancomycin-resistant Enterococci faecalis A4 (VRE), *Bacillus subtilis* ATCC 6051, *Pseudomonas aeruginosa* 14, and *Klebsiella pneumoniae* WNX-1. As shown in Table 5, the unsaturated *γ*-lactone **6** exhibited significant inhibition against a panel of bacterial strains, of which the MIC value toward VRE was 1.63 μg/mL. The pyrone derivative **8** selectively inhibited VRE and *B. subtilis* with an MIC value of 12.9 μg/mL. The remaining compounds showed MIC values higher than 64 μg/mL.

## 3. Discussion

Fungal species of the *Trichoderma* genus are recognized as biological control agents against plant-associated fungal pathogens, with the biological effects of these fungal strains due to the profiles of fungus-derived metabolites. Pentylpyrone from *T. koningii* inhibited the growth of several soilborne plant pathogens [15], while sorbicillin-related analogues exerted radical scavenging and other bioactivities [34]. These findings suggested that the *Trichoderma* fungus inhabiting the host sponges produces sorbicillinoids, which may play chemoecological role. In the present work, the fungus host, sponge *Dictyonella incisa*, was reported to produce conjugated and degraded sterols and fatty acids without cytotoxicity [35,36,37]. These data, along with the various bioactivities of sorbicillinoids, suggested that the endophytic fungus *T. saturnisporum* produced sorbicillinoid-based derivatives to help the host sponge in its defense against pathogenic invasion. For a pharmaceutical point of view, the pathogenic bacterium *E. faecium* is often resistant to vancomycin and ampicillin, and is difficult to treat by antibacterial agents [38]. The potent anti-VRE effect of the unsaturated *γ*-lactone (**6**) provides a new natural scaffold for further development as a promising anti-VRE lead after structural modification.

## 4. Materials and Methods

### 4.1. General Experimental Procedures

Optical rotations were measured on a Rudolph IV Autopol automatic polarimeter. IR spectra were recorded on a Thermo Nicolet Nexus 470 FT-IR spectrometer. ^1^H and ^13^C NMR as well as 2D NMR spectra were recorded on a Bruker Avance 400 NMR spectrometer (400 MHz for ^1^H and 100 MHz for ^13^C, respectively). Chemical shifts are expressed in *δ* (ppm), referenced to the solvent peaks at *δ*_H_ 3.31 and *δ*_C_ 49.3 for CD_3_OD or *δ*_H_ 2.05 and *δ*_C_ 30.2, 206.8 for acetone-*d*_6_, and coupling constants are in Hz. The temperature for NMR measurement is 298 K, the coupling constant for HMBC experiments is optimized as 8 Hz, and the mixing time used for ROESY experiments is 2 s. HREIMS spectra were obtained from an Autospec Ultima-TOF spectrometer. TLC: Precoated Si gel G plates (Qingdao Marine Chemical Factory, Qingdao, China). Silica gels (200–300 mesh, Qingdao Marine Chemistry Co., Ltd.) and octadecylsilyl silica (ODS) (YMC) were used for column chromatography. Semi-preparative HPLC chromatography was performed on an Alltech instrument (426-HPLC pump) equipped with an Alltech uvis-200 detector at 210 nm, and semi-preparative reversed-phase columns (YMC-packed C_18_, 5 μm, 250 mm × 10 mm) were purchased from YMC Co LTD (Kyoto, Japan). ECD spectra were obtained on a Jasco J-810 spectropolarimeter. The bacterium strains (*Staphylococcus aureus* ATCC 29213, *Bacillus subtilis* ATCC 6051) were purchased from American Type Culture Collection (ATCC), and vancomycin-resistant *Enterococci faecalis* A4 (VRE), *Pseudomonas aeruginosa* 14, and *Klebsiella pneumoniae* WNX-1 were supplied by Prof. Kui Zhu (the Microbiology Center, Agriculture University of China, Beijing, China).

### 4.2. Fungal Strain

The fungus, strain DI-IA, was isolated from the sponge *Dictyonella incisa* collected at a depth of 10 m in the Seferihisar bay of Turkey. It was identified as *Trichoderma saturnisporum* on the basis of its ribosomal internal transcribed spacers (ITS) and the 5.8S ribosomal RNA gene (ITS1-5.8S-ITS2). Working stocks were prepared on potato dextrose agar slants stored at 4 °C in our laboratory.

### 4.3. Fermentation, Extraction, and Isolation

The fermentation was carried out in Fernbach flasks (500 mL), which contained 80 g of rice. Distilled H_2_O (120 mL) was added to the flask, and the contents were soaked overnight before autoclaving at 15 psi for 30 min. After cooling to room temperature (rt), each flask was inoculated with 5.0 mL of the spore inoculums and incubated at 25 °C for 40 days. The fermented material was extracted successively with EtOAc (3 × 500 mL), and then evaporated to dryness under vacuum to afford a crude extract. The crude extracts were tested against *S. aureus* and showed an inhibitory effect with an MIC value of 64 μg/mL. Thus, the EtOAc extract was detected by the ^1^H NMR spectrum, showing the rich conjugated olefinic resonances in the range of 5–7 ppm.

The EtOAc extract (1.1 g) extract was subjected to silica gel vacuum liquid chromatography (VLC), eluting with a gradient of petroleum/ethyl acetate (from 50:0 to 1:1, *v*:*v*) and CH_2_Cl_2_-MeOH (from 20:1 to 10:1, *v*:*v*) to obtain nine fractions (F1–F9). F9 (90 mg) was subjected to semi-preparative HPLC (ODS, 26 × 310 mm, 2 mL/min, UV detection at 400 nm) with MeOH-H_2_O-TFA (60:40:0.1) as a mobile phase to give **2** (15 mg). F8 (120 mg) was purified by RP- HPLC (YMC-packed C18, 5 μm, 250 × 10 mm, 2 mL/min, UV detection at 210 nm), eluting with MeOH-H_2_O-TFA (45:55:0.1) to yield **5** (4.1 mg), **6** (11.1 mg), **7** (6.9 mg), and **8** (7.6 mg). F7 (150 mg) was separated by RP-HPLC (YMC-packed C_18_, 5 μm, 250 × 10 mm, 2 mL/min, UV detection at 210 nm) eluting with MeOH-H_2_O-TFA (71:29:0.1) to obtain **1** (10 mg), **4** (4.2 mg), and **3** (7.5 mg). The IR, HRESIMS, 1D and 2D NMR spectra of the compounds were provided in the Supporting Information Figures S1–S62.

*Saturnispol A* (**1**): red oil. [α]_D_ = −445.5 (*c* 1.1, MeOH); UV (MeOH) (log ε) *λ*_max_ 228, 272, 32, 401 nm; IR (MeOH) *γ*_max_ 3380, 2919, 2850, 1738, 1614, 1555, 1366 cm^−1^; ^1^H NMR (CD_3_OD, 400 MHz) and ^13^C NMR (CD_3_OD, 100 MHz) data, see Table 1; HRESIMS *m*/*z* 529.2069 [M − H]^−^ (calcd for C_28_H_33_O_10_, 529.2074).

*Saturnispol B* (**2**): yellow oil. [α]_D_ = −367.3 (*c* 1.1, MeOH); UV (MeOH) (log *ε*) *λ*_max_ 238, 278, 336, 402 nm; IR (MeOH) *ν*_max_ 3404, 2970, 2928, 1736, 1714, 1614, 1560 cm^−1^; ^1^H NMR (acetone-*d*_6_, 400 MHz) and ^13^C NMR (acetone-*d*_6_, 100 MHz) data, see Table 1; HRESIMS *m*/*z* 513.2125 [M − H]^−^ (calcd for C_28_H_33_O_9_, 513.2125).

*Saturnispol C* (**3**): yellow oil. [α]_D_ = +20.0 (*c* 0.12, MeOH); UV (MeOH) (log *ε*) *λ*_max_ 367 nm; IR (MeOH) *ν*_max_ 3429, 2925, 2853, 1730, 1712, 1601, 1453, 1377, 1230 cm^−1^; ^1^H NMR (CDCl_3_, 400 MHz) and ^13^C NMR (CDCl_3_, 100 MHz) data, see Table 2; HRESIMS *m*/*z* 351.1600 [M − H]^−^ (calcd for C_22_H_23_O_4_, 351.1596).

*Saturnispol D* (**4**): yellow oil. [α]_D_ = +26.0 (*c* 0.08, MeOH); UV (MeOH) (log *ε*) *λ*_max_ 339, 360 nm; IR (MeOH) *ν*_max_ 3439, 2926, 2854, 1771, 1732, 1617, 1440, 1390 cm^−1^; ^1^H NMR (acetone-*d*_6_, 400 MHz) and ^13^C NMR (acetone-*d*_6_, 100 MHz) data, see Table 2; HRESIMS *m*/*z* 367.1544 [M − H]^−^ (calcd for C_22_H_23_O_5_, 367.1545).

*Saturnispol E* (**5**): yellow oil. [α]_D_ = +43.1 (*c* 1.3, MeOH); UV (MeOH) (log ε) *λ*_max_ 229, 264 nm; IR (MeOH) *ν*_max_ 3365, 2927, 1740, 1713, 1623, 1454 cm^−1^; ^1^H NMR (CD_3_OD, 400 MHz) and ^13^C NMR (CD_3_OD, 100 MHz) data, see Table 3 and Table 4; HRESIMS *m*/*z* 267.1237 [M − H]^−^ (calcd for C_14_H_19_O_5_, 267.1232).

*Saturnispol F* (**6**): light yellow oil. [α]_D_ = −15 (*c* 1.0, CHCl_3_); UV (MeOH) (log *ε*) *λ*_max_ 250, 272, 372 nm; IR (MeOH) *ν*_max_ 3422, 2924, 2853, 1737, 1655, 1640, 1366 cm^−1^; ^1^H NMR (CD_3_OD, 400 MHz) and ^13^C NMR (CD_3_OD, 100 MHz) data, see Table 3 and Table 4; HRESIMS *m*/*z* 265.1073 [M − H]^−^ (calcd for C_14_H_17_O_5_, 265.1076).

*Saturnispol G* (**7**): yellow oil. UV (MeOH) (log *ε*) *λ*_max_ 222, 324 nm; IR (MeOH) *ν*_max_ 3437, 2923, 1670, 1631, 1570, 1411 cm^−1^; ^1^H NMR (acetone-*d*_6_, 400 MHz) and ^13^C NMR (acetone-*d*_6_, 100 MHz) data, see Table 3 and Table 4; HRESIMS *m*/*z* 209.0811 [M − H]^−^ (calcd for C_11_H_13_O_4_, 209.0814).

*Saturnispol H* (**8**): light yellow oil. UV (MeOH) (log *ε*) *λ*_max_ 219, 347 nm; IR (MeOH) *ν*_max_ 3438, 2919, 2850, 1736, 1653, 1381, 1367, 1229 cm^−1^; ^1^H NMR (CD_3_OD, 400 MHz) and ^13^C NMR (CD_3_OD, 100 MHz) data, see Table 3 and Table 4; HRESIMS *m*/*z* 207.0657 [M − H]^−^ (calcd for C_11_H_11_O_4_, 207.0657).

### 4.4. ECD Calculation

Conformational analyses were carried out via random searching in the Sybyl-X 2.0 using the MMFF94S force field with an energy cutoff of 2.5 kcal/mol. Subsequently, the conformers were re-optimized using discrete fourier transform (DFT) at the B3LYP/6-31++G (d) level in the gas phase using the GAUSSIAN 09 program. The energies, oscillator strengths, and rotational strengths (velocity) of the first 60 electronic excitations were calculated using the time-dependent density functional theory (TDDFT) methodology at the B3LYP/6-311++G (2d, 2p) level in vacuum. The ECD spectra were simulated by the overlapping Gaussian function (half the bandwidth at 1/e peak height, *σ* = 0.2 eV). To obtain the final spectra, the simulated spectra of the conformers for each structure were averaged according to the Boltzmann distribution theory and their relative Gibbs free energy (ΔG). Theoretical ECD spectra of the corresponding enantiomers were obtained by the direct inversions of the ECD spectra of the abovementioned compounds.

### 4.5. Antibacterial Assay

All compounds were tested for antibacterial activities against Gram-positive bacteria, *S. aureus* (ATCC 29213), *B. subtilis*, and vancomycin-resistant *E. faecalis* A4; Gram-negative *P. aeruginosa* 14; and pathogenic bacterium K. pneumoniae. The minimum inhibitory concentrations (MIC) were determined using the broth micro-dilution method according to the clinical and laboratory standards institute (CLSI) 2015 guideline. The commercial antimicrobial agents Ciprofloxacin, Colistin, and Bacaucin were used as positive controls. After 16–20 h incubation at 37 °C, MIC values were defined as the lowest concentrations of antibiotics with no visible growth of bacteria. Values of more than 64 μg/mL were considered as no antibacterial activity.

## 5. Conclusions

Our work first explored the secondary metabolites of *T. saturnisporum* derived from a marine sponge, and continued to uncover eight new sorbicillin-based derivatives. This finding provided additional evidence to support the assumption that the same fungal species from a marine environment are capable of activating a distinct biosynthetic pathway, like those of terrestrial origin. Saturnispols A–H as the sorbicillin-based derivatives are classified into five different scaffolds, while a terminal hydroxylation at the side chain is rare in the sorbicillinold family. In the biogenetic consideration, these compounds were generated from sorbicillinol as a precursor to derive different skeletons. Compound **6** serves as a new natural scaffold that possesses significant inhibition against drug-resistant bacterium (VRE), suggesting it to be a potential lead to overcome certain drug-resistant pathogens after structural optimization. In addition, both *K. pneumonia and P. aeruginosa* are Gram-negative, indicating that they are resistant to a large range of antibiotics, resulting in clinically unsuccessful treatment. The significant inhibition of **6** against these two pathogens suggests that it may be a promising agent against Gram-negative bacteria.

## Figures and Tables

**Table 1:** ^1^H and ^13^C NMR data of **1**–**2** (*δ* (ppm), *J* (Hz)).

	1 ^a^		2 ^b^	
	*δ*_H_	*δ*_C_	*δ*_H_	*δ*_C_
1		193.6		193.1
2		105.1		104.8
3	2.75, d (14.6) 2.47, d (14.6)	35.1	2.83, d (14.0) 2.53, d (14.0)	35.6
4		72.8		72.8
4a		105.9		106.1
5a		79.2		78.9
6		168.3		166.7
7		109.3		108.9
8		191.9		191.7
9		101.7		101.6
9a	3.67, s	53.7	3.75, s	53.8
9b		59.2		58.7
10		177.4		179.2
11	6.42, d (15.2)	122.3	6.40, d (14.4)	121.1
12	7.28, dd (11.2, 15.2)	141.0	7.25, dd (14.4, 12.0)	141.9
13	6.54, dd (11.2, 15.2)	128.3	6.39, dd (14.4, 12.0)	131.1
14	6.27, dt (5.2, 15.2)	141.8	6.24, dq (14.4, 6.8)	139,2
15	4.22, t (5.2)	61.5	1.87, d (6.8)	18.1
16	1.22, s	21.3	1.19, s	21.5
17	1.45, s	24.5	1.45, s	25.1
18	1.43, s	5.74	1.45, s	6.2
19		166.5		167.6
20	6.62, d (15.2)	122.5	6.72, d (14.4)	122.8
21	7.25, dd (11.2, 15.2)	136.9	7.25, dd (14.4, 12.0)	137.1
22	6.54, dd (11.2, 15.2)	128.9	6.63, dd (14.4, 12.0)	128.6
23	6.18, dt (5.2, 15.2)	138.6	6.23, dd (14.4, 4.6)	139.9
24	4.22, d (5.2)	61.6	4.23, d (4.6)	62.1
25	1.29, s	18.7	1.34, s	19.0

^a^ Measured in CD_3_OD; ^b^ measured in acetone-*d*_6_.

**Table 2:** ^1^H and ^13^C NMR data of **3**–**4** (*δ* (ppm), *J* (Hz)).

	3 ^a^		4 ^b^	
	*δ*_H_	*δ*_C_	*δ*_H_	*δ*_C_
1		64.6		64.8
2		197.7		198.4
3		112.0		112.9
4	3.32, t (2.8)	40.5	3.46, t (2.8)	40.7
5		74.8		73.7
6		211.7		209.2
7	3.15, dd (10.8, 5.6)	47.7	3.24, dd (10.8, 6.0)	46.4
8	3.05, ddd (13.5, 10.8, 2.8) 1.90, ddd (13.5, 5.6, 2.8)	31.4	3.09, ddd (13.6, 10.8, 2.8) 1.87, ddd (13.6, 6.0, 2.8)	31.5
9		167.1		166.8
10	6.30, d (14.8)	118.1	6.75, d (15.2)	120.3
11	7.41, dd (14.8, 10.8)	142.4	7.43, dd (15.2, 10.8)	141.0
12	6.33, dd (14.8, 10.8)	130.9	6.68, dd (15.2, 10.8)	127.7
13	6.25, dq (14.8, 6.8)	139.7	6.39, dt (15.2, 4.4)	142.9
14	1.94, d (6.8)	18.9	4.28, t (4.4)	61.7
15		141.4		142.1
16	6.98, d (7.6)	128.4	7.07, d (7.6)	128.4
17	7.26, t (7.6)	128.6	7.29, t (7.6)	128.4
18	7.26, t (7.6)	127.4	7.26, t (7.6)	127.0
19	7.26, t (7.6)	128.6	7.29, t (7.6)	128.4
20	6.98, d (7.6)	128.4	7.07, d (7.6)	128.4
21	0.93, s	10.5	0.80, s	10.4
22	1.29, s	24.4	1.26, s	23.2
OH-5	2.69, s		5.05, s	
OH-9	14.34, s		14.40, s	
OH-14			4.10, t (4.4)	

^a^ Measured in CDCl_3_; ^b^ measured in acetone-*d*_6_.

**Table 3:** ^1^H NMR data of **5**–**8** (*δ* (ppm), *J* (Hz), 400 MHz).

	5 ^a^	6 ^a^	7 ^b^	8 ^a^
4			6.01, s	6.08, s
5	1.96, dd (12.8, 4.8) 1.66, dd (12.8, 12.0)	2.07, t (7.6)		
6	2.69, ddd (12.0, 7.4, 4.8)	2.50, t (7.6)	6.11, d (16.0)	6.19, d (15.2)
7	4.49, t (7.4)		6.63, dt (16.0, 7.2)	7.06, dd (15.2, 10.8)
8	5.65, dd (14.4, 7.4)	6.18, d (16.0)	2.32, dt (7.2, 7.6)	6.45, dd (15.2, 10.8)
9	6.31, dd (14.4, 10.6)	7.25, dd (16.0, 10.6)	1.71, tt (7.6, 6.4)	6.15, dt (15.2, 5.0)
10	6.29, dd (14.4, 10.6)	6.47, dd (16.0, 10.6)	3.61, t (6.4)	4.20, d (5.0)
11	5.86, dt (14.4, 5.6)	6.36, dt (16.0, 4.6)	1.89, s	1.90, s
12	4.12, d (5.6)	4.22, d (4.6)		
13	1.34, s	1.44, s		
14	1.68, s	1.66, s		

^a^ Measured in CD_3_OD; ^b^ measured in acetone-*d*_6_.

**Table 4:** ^13^C NMR data of **5**–**8**.

	5 ^a^	6 ^a^	7 ^b^	8 ^a^
1	183.2	176.8	164.4	166.7
2	108.4	93.6	98.9	99.2
3	192.2	180.8	156.1	156.5
4	71.1	83.0	99.6	101.4
5	37.1	30.6	164.8	167.1
6	43.9	33.7	122.2	121.9
7	74.2	200.6	137.1	134.0
8	131.6	128.5	28.8	128.4
9	132.3	142.9	31.8	138.4
10	129.4	127.4	60.7	61.6
11	133.5	143.6	8.0	7.2
12	61.7	61.4		
13	24.0	22.2		
14	6.4	4.6		

^a^ Measured in CD_3_OD; ^b^ measured in acetone-*d*_6_.

**Table 5:** Antimicrobial activities of compounds **1**–**8.**

Compounds	MIC (μg/mL)				
	*S. Aureus*	VRE	*B. Subtilis*	*P. Aeruginosa*	*K. Pneumonica*
**1**	>64	>64	>64	>64	>64
**2**	>64	>64	>64	>64	>64
**3**	>64	>64	>64	>64	>64
**4**	>64	>64	>64	>64	>64
**5**	>64	>64	>64	>64	>64
**6**	3.32	1.63	>64	6.65	6.65
**7**	>64	>64	>64	>64	>64
**8**	>64	12.9	12.9	>64	>64
ciprofloxacin	0.125	ND	0.125	ND	ND
colistin	ND	ND	ND	0.125	0.125
bacaucin	ND	16	ND	ND	ND

MIC: minimum inhibitory concentrations, ND = no detection.

## Supplementary Materials

The following are available online at http://www.mdpi.com/1660-3397/16/7/226/s1, HRESIMS, ^1^H, ^13^C NMR, HSQC, HMBC, ROESY, and IR spectra of the new compounds.
