# Supplementary material for: Sorbicillinoid-Based Metabolites from a Sponge-Derived Fungus Trichoderma saturnisporum
Source: Mar Drugs. 2018 Jul 4;16(7):226. doi: 10.3390/md16070226 (PMC6071017; doi:10.3390/md16070226)
Supplement: Supplementary file 1 [file marinedrugs-16-00226-s001.pdf]

List of Contents

no.	Content	Page
1	**Figure 1.** The IR Spectrum of Compound **1**	1
2	**Figure 2.** The HRESIMS Data of Compound **1**	2
3	**Figure 3.** The ^{1}H NMR Spectrum of Compound **1** in CD_3OD (400 MHz)	3
4	**Figure 4.** The ^{13}C APT Spectrum of Compound **1** in CD_3OD (100 MHz)	4
5	**Figure 5.** The ^{1}H-^{1}H COSY Spectrum of Compound **1** in CD_3OD (400 MHz)	5
6	**Figure 6.** The HSQC Spectrum of Compound **1** in CD_3OD (400 MHz for ^{1}H)	6
7	**Figure 7.** The HMBC Spectrum of Compound **1** in CD_3OD (400 MHz for ^{1}H)	7
8	**Figure 8.** The ROESY Spectrum of Compound **1** in CD_3OD (400 MHz)	8
9	**Figure 9.** The IR Spectrum of Compound **2**	9
10	**Figure 10.** The HRESIMS Data of Compound **2**	10
11	**Figure 11.** The ^{1}H NMR Spectrum of Compound **2** in Acetone-d_6 (400 MHz)	11
12	**Figure 12.** The ^{13}C CPD Spectrum of Compound **2** in Acetone-d_6 (100 MHz)	12
13	**Figure 13.** The ^{1}H-^{1}H COSY Spectrum of Compound **2** in Acetone-d_6 (400 MHz)	13
14	**Figure 14.** The HSQC Spectrum of Compound **2** in Acetone-d_6 (400 MHz for ^{1}H)	14
15	**Figure 15.** The HMBC Spectrum of Compound **2** in Acetone-d_6 (400 MHz for ^{1}H)	15
16	**Figure 16.** The ROESY Spectrum of Compound **2** in Acetone-d_6 (400 MHz)	16
17	**Figure 17.** The IR Spectrum of Compound **3**	17
18	**Figure 18.** The HRESIMS Data of Compound **3**	18
19	**Figure 19.** The ^{1}H NMR Spectrum of Compound **3** in $CDCl_3$ (400 MHz)	19
20	**Figure 20.** The ^{13}C CPD Spectrum of Compound **3** in $CDCl_3$ (100 MHz)	20
21	**Figure 21.** The ^{1}H-^{1}H COSY Spectrum of Compound **3** in $CDCl_3$ (400 MHz)	21
22	**Figure 22.** The HSQC Spectrum of Compound **3** in $CDCl_3$ (400 MHz for ^{1}H)	22
23	**Figure 23.** The HMBC Spectrum of Compound **3** in $CDCl_3$ (400 MHz for ^{1}H)	23
24	**Figure 24.** The ROESY Spectrum of Compound **3** in $CDCl_3$ (400 MHz)	24
25	**Figure 25.** The IR Spectrum of Compound **4**	25
26	**Figure 26.** The HRESIMS Data of Compound **4**	26
27	**Figure 27.** The ^{1}H NMR Spectrum of Compound **4** in Acetone-d_6 (400 MHz)	27
28	**Figure 28.** The ^{13}C CPD Spectrum of Compound **4** in Acetone-d_6 (100 MHz)	28
29	**Figure 29.** The ^{1}H-^{1}H COSY Spectrum of Compound **4** in Acetone-d_6 (400 MHz)	29
30	**Figure 30.** The HSQC Spectrum of Compound **4** in Acetone-d_6 (400 MHz for ^{1}H)	30
31	**Figure 31.** The HMBC Spectrum of Compound **4** in Acetone-d_6 (400 MHz for ^{1}H)	31

32	**Figure 32.** The ROESY Spectrum of Compound **4** in Acetone-d_6 (400 MHz)	32
33	**Figure 33.** The IR Spectrum of Compound **5**	33
34	**Figure 34.** The HRESIMS Data of Compound **5**	34
35	**Figure 35.** The ^{1}H NMR Spectrum of Compound **5** in CD_3OD (400 MHz)	35
36	**Figure 36.** The ^{13}C CPD Spectrum of Compound **5** in CD_3OD (100 MHz)	36
37	**Figure37.** The ^{1}H-^{1}H COSY Spectrum of Compound **5** in CD_3OD (400 MHz)	37
38	**Figure 38.** The HSQC Spectrum of Compound **5** in CD_3OD (400 MHz for ^{1}H)	38
39	**Figure 39.** The HMBC Spectrum of Compound **5** in CD_3OD (400 MHz for ^{1}H)	39
40	**Figure 40.** The ROESY Spectrum of Compound **5** in CD_3OD (400 MHz)	40
41	**Figure 41.** The IR Spectrum of Compound **6**	41
42	**Figure 42.** The HRESIMS Data of Compound **6**	42
43	**Figure 43.** The ^{1}H NMR Spectrum of Compound **6** in CD_3OD (400 MHz)	43
44	**Figure 44.** The ^{13}C APT Spectrum of Compound **6** in CD_3OD (100 MHz)	44
45	**Figure 45.** The ^{1}H-^{1}H COSY Spectrum of Compound **6** in CD_3OD (400 MHz)	45
46	**Figure 46.** The HSQC Spectrum of Compound **6** in CD_3OD (400 MHz for ^{1}H)	46
47	**Figure 47.** The HMBC Spectrum of Compound **6** in CD_3OD (400 MHz for ^{1}H)	47
48	**Figure 48.** The ROESY Spectrum of Compound **6** in CD_3OD (400 MHz)	48
49	**Figure 49.** The IR Spectrum of Compound **7**	49
50	**Figure 50.** The HRESIMS Data of Compound **7**	50
51	**Figure 51.** The ^{1}H NMR Spectrum of Compound **7** in Acetone-d_6 (400 MHz)	51
52	**Figure 52.** The ^{13}C APT Spectrum of Compound **7** in Acetone-d_6 (100 MHz)	52
53	**Figure 53.** The ^{1}H-^{1}H COSY Spectrum of Compound **7** in Acetone-d_6 (400 MHz)	53
54	**Figure 54.** The HSQC Spectrum of Compound **7** in Acetone-d_6 (400 MHz for ^{1}H)	54
55	**Figure 55.** The HMBC Spectrum of Compound **7** in Acetone-d_6 (400 MHz for ^{1}H)	55
56	**Figure 56.** The IR Spectrum of Compound **8**	56
57	**Figure 57.** The HRESIMS Data of Compound **8**	57
58	**Figure 58.** The ^{1}H NMR Spectrum of Compound **8** in CD_3OD (400 MHz)	58
59	**Figure 59.** The ^{13}C APT Spectrum of Compound **8** in CD_3OD (100 MHz)	59
60	**Figure 60.** The ^{1}H-^{1}H COSY Spectrum of Compound **8** in CD_3OD (400 MHz)	60
61	**Figure 61.** The HSQC Spectrum of Compound **8** in CD_3OD (400 MHz for ^{1}H)	61
62	**Figure 62.** The HMBC Spectrum of Compound **8** in CD_3OD (400 MHz for ^{1}H)	62

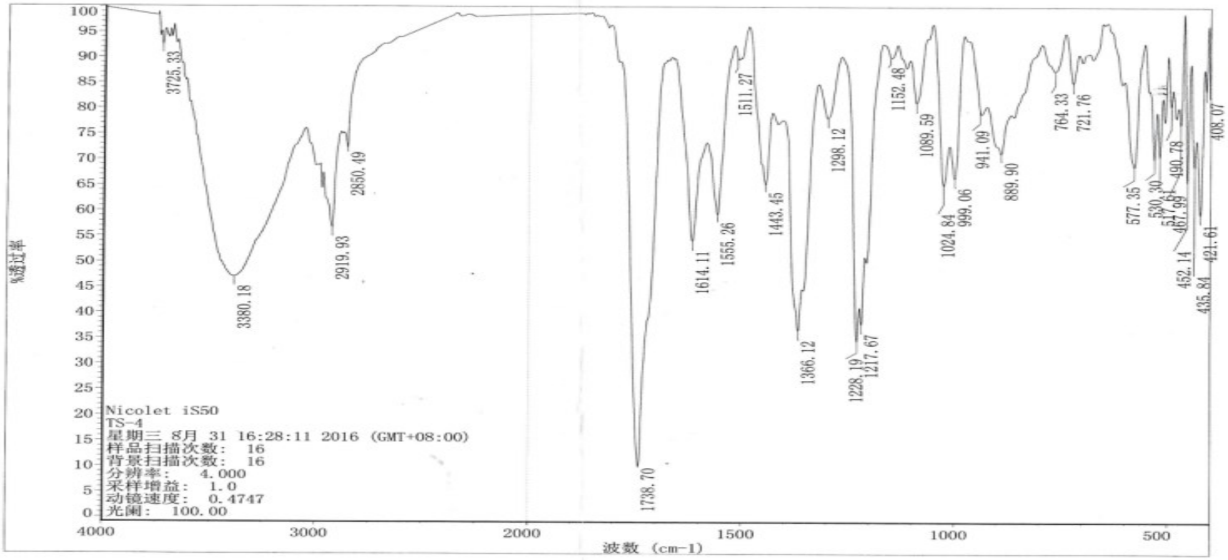

Figure 1. The IR Spectrum of Compound **1**

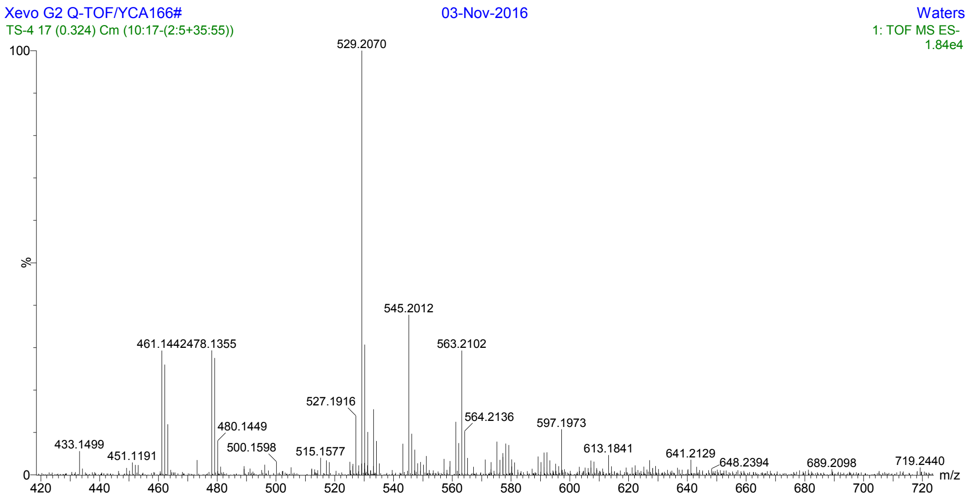

Figure 2. The HRESIMS Data of Compound **1**

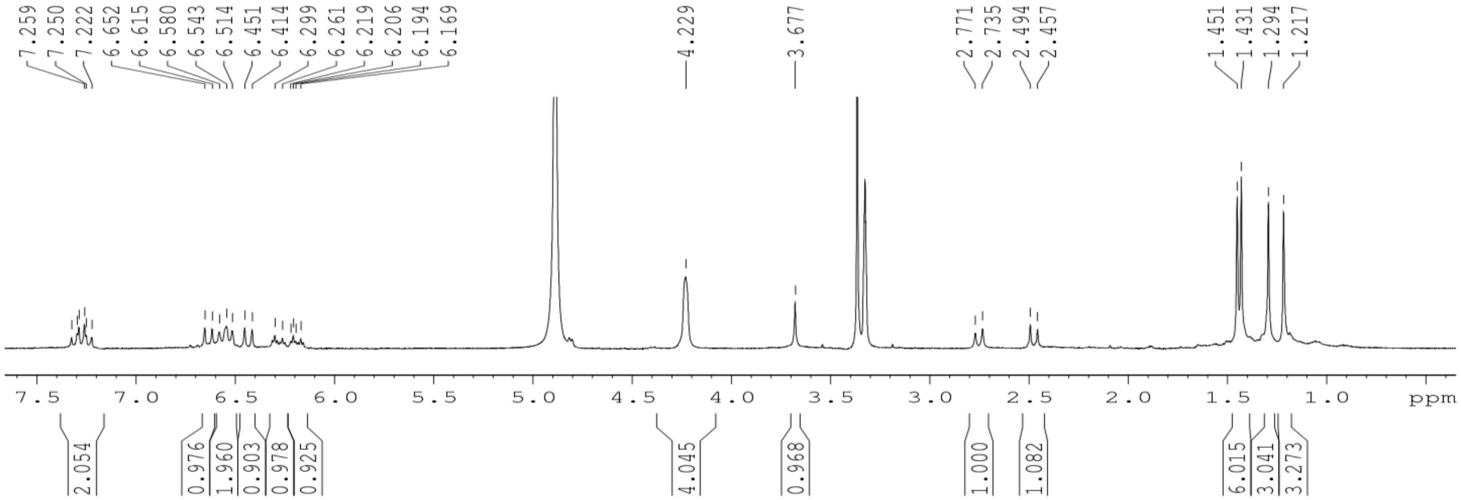

Figure 3. The ^{1}H NMR Spectrum of Compound **1** in CD_3OD (400 MHz)

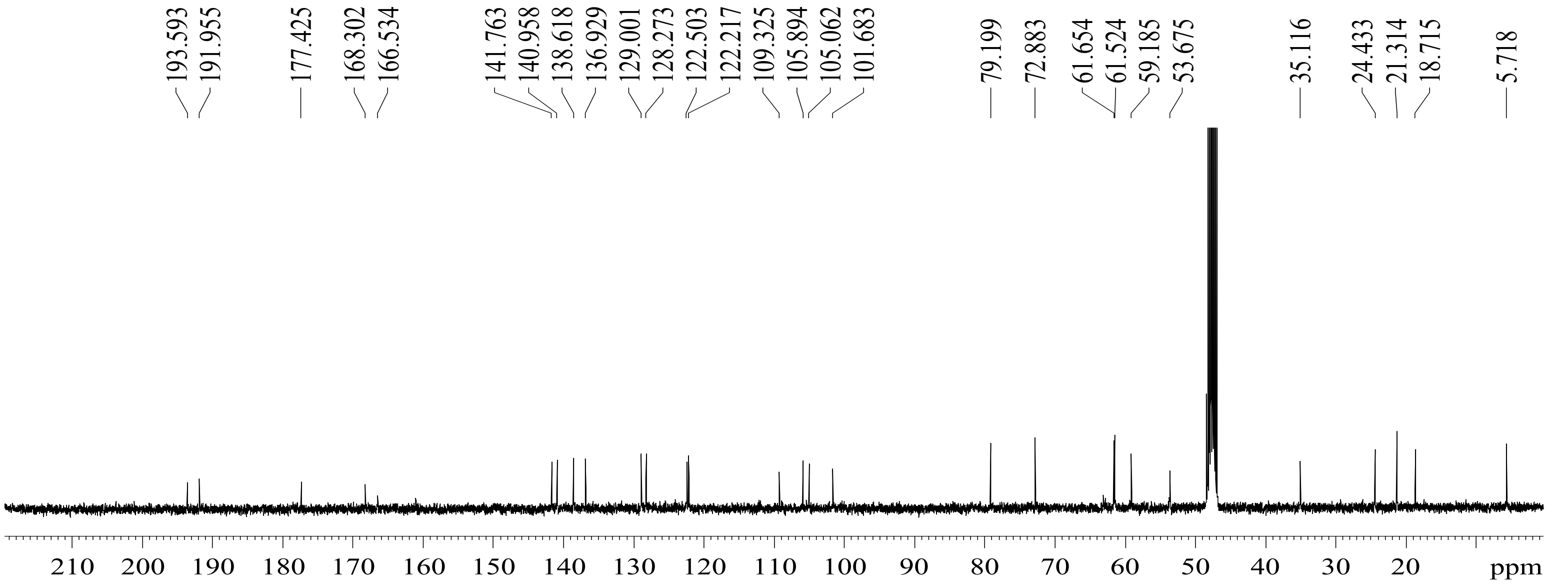

Figure 4. The ^{13}C CPD NMR Spectrum of Compound **1** in CD_3OD (100 MHz)

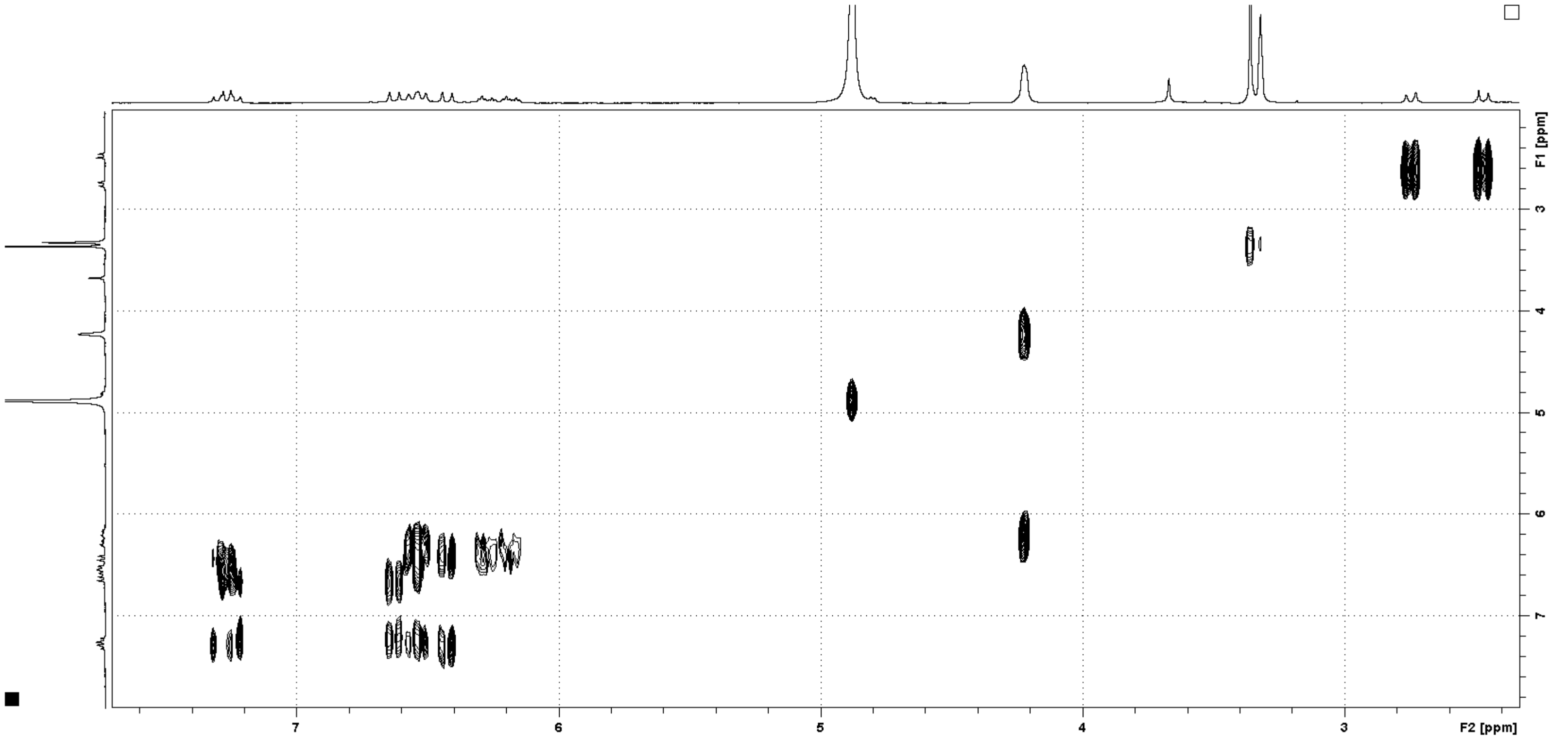

Figure 5. The ^{1}H-^{1}H COSY Spectrum of Compound **1** in CD_3OD (400 MHz)

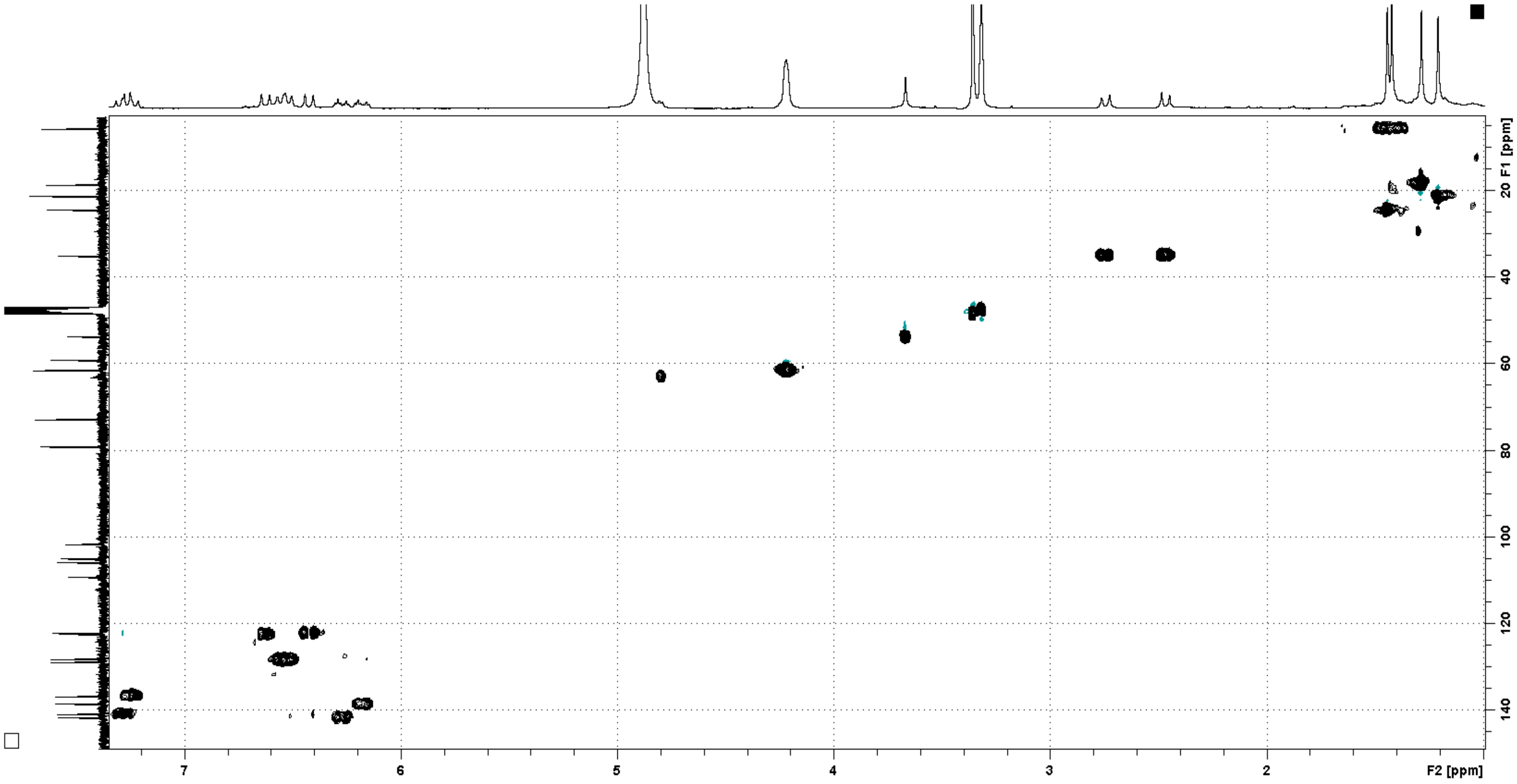

Figure 6. The HSQC Spectrum of Compound **1** in CD_3OD (400 MHz for ^1H)

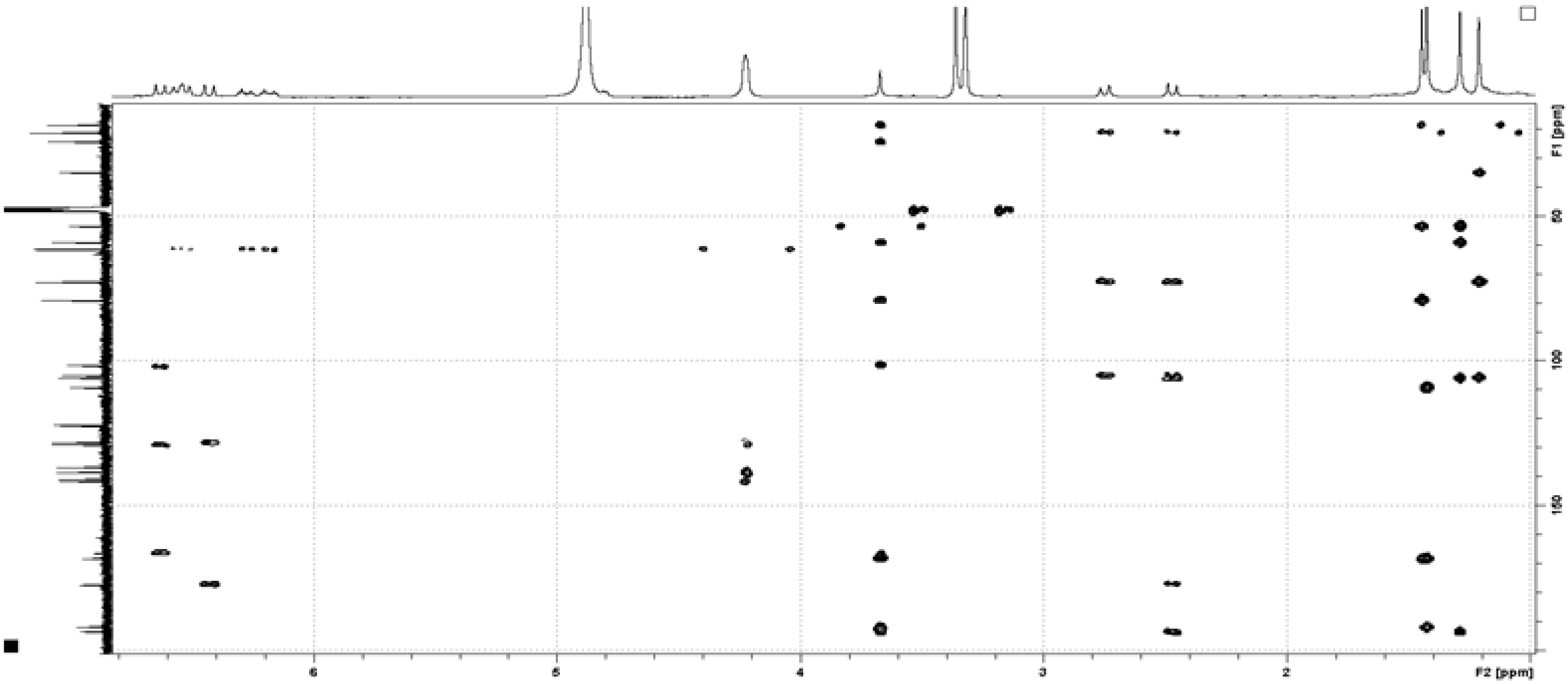

Figure 7. The HMBC Spectrum of Compound **1** in CD_3OD (400 MHz for ^1H)

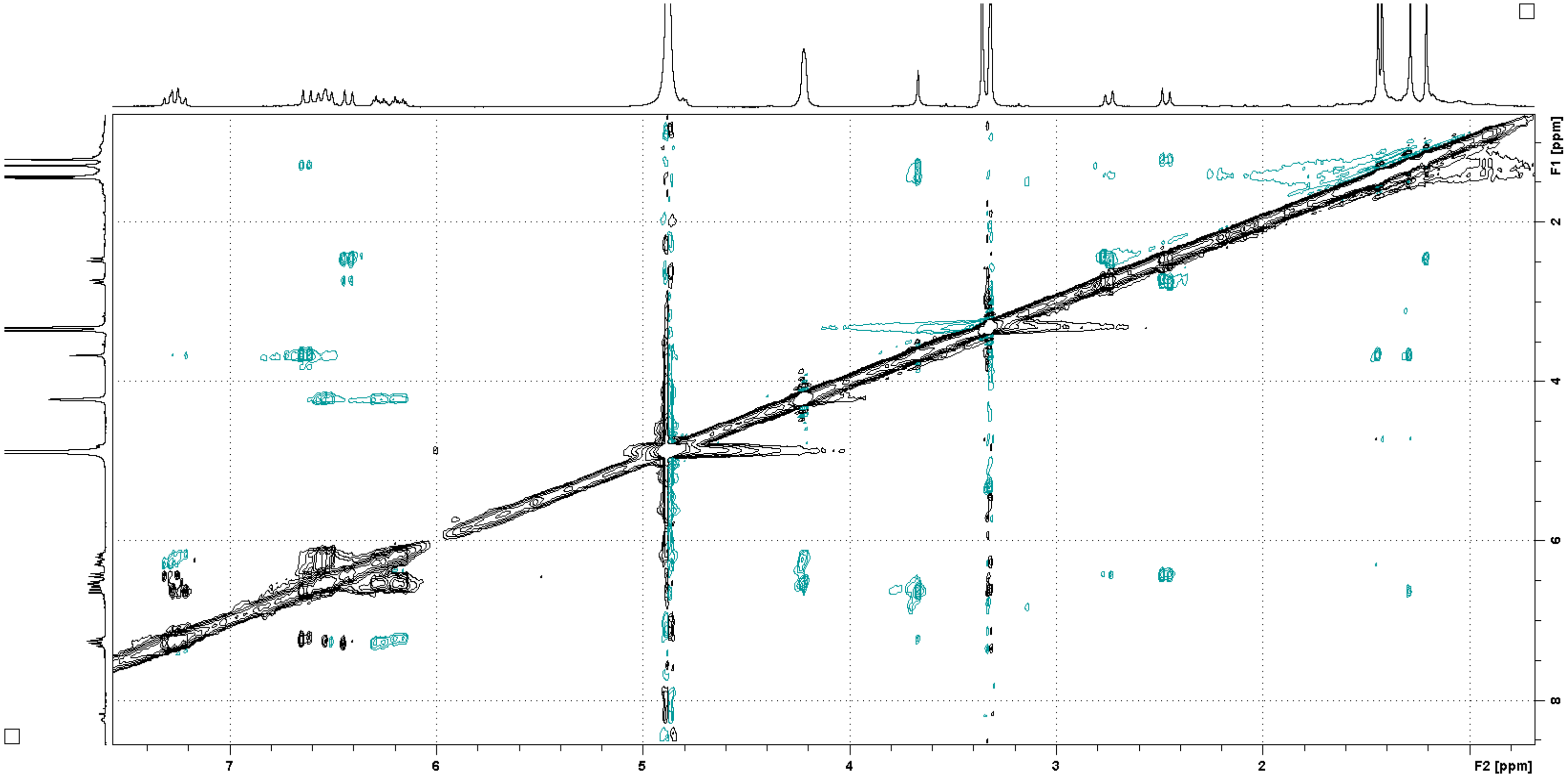

Figure 8. The ROESY Spectrum of Compound **1** in CD_3OD (400 MHz)

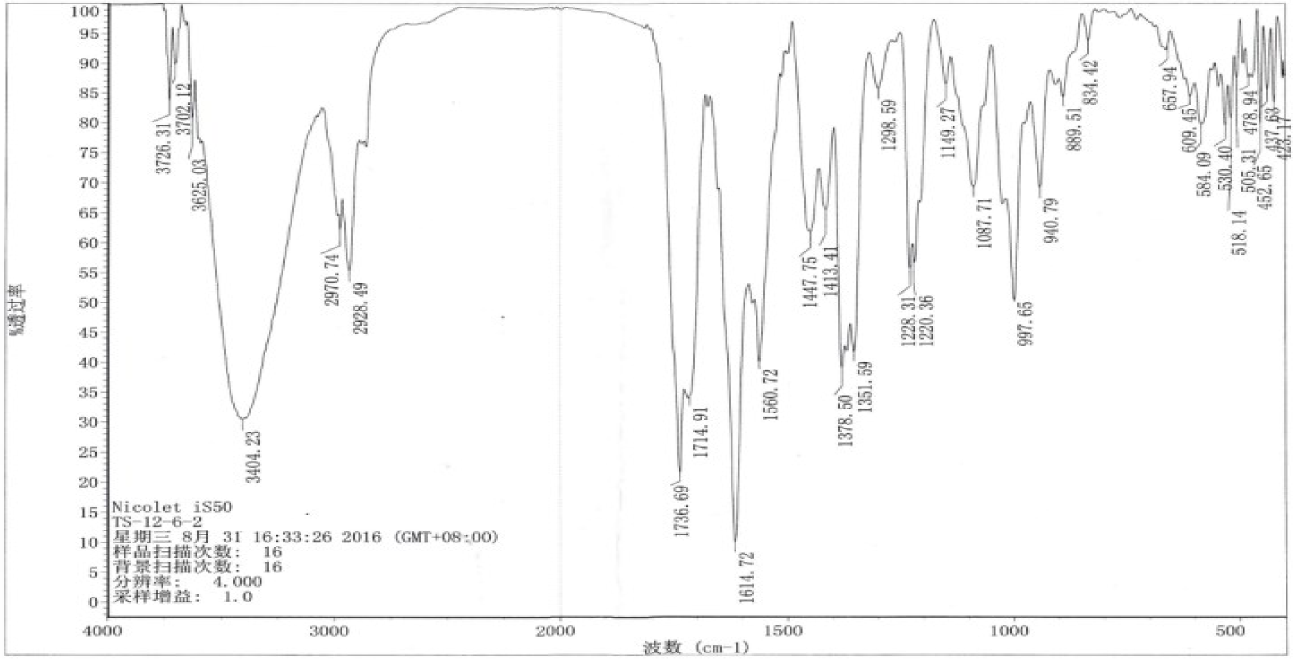

Figure 9. The IR Spectrum of Compound **2**

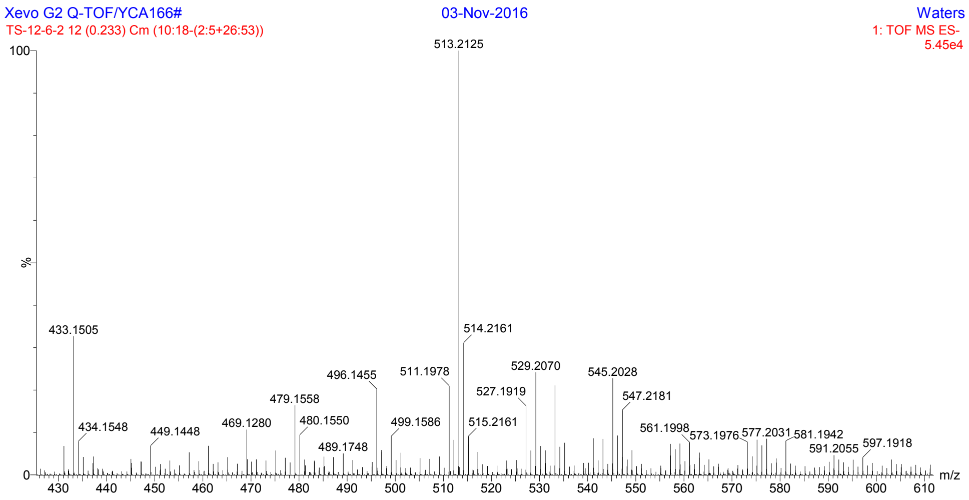

Figure 10. The HRESIMS Data of Compound **2**

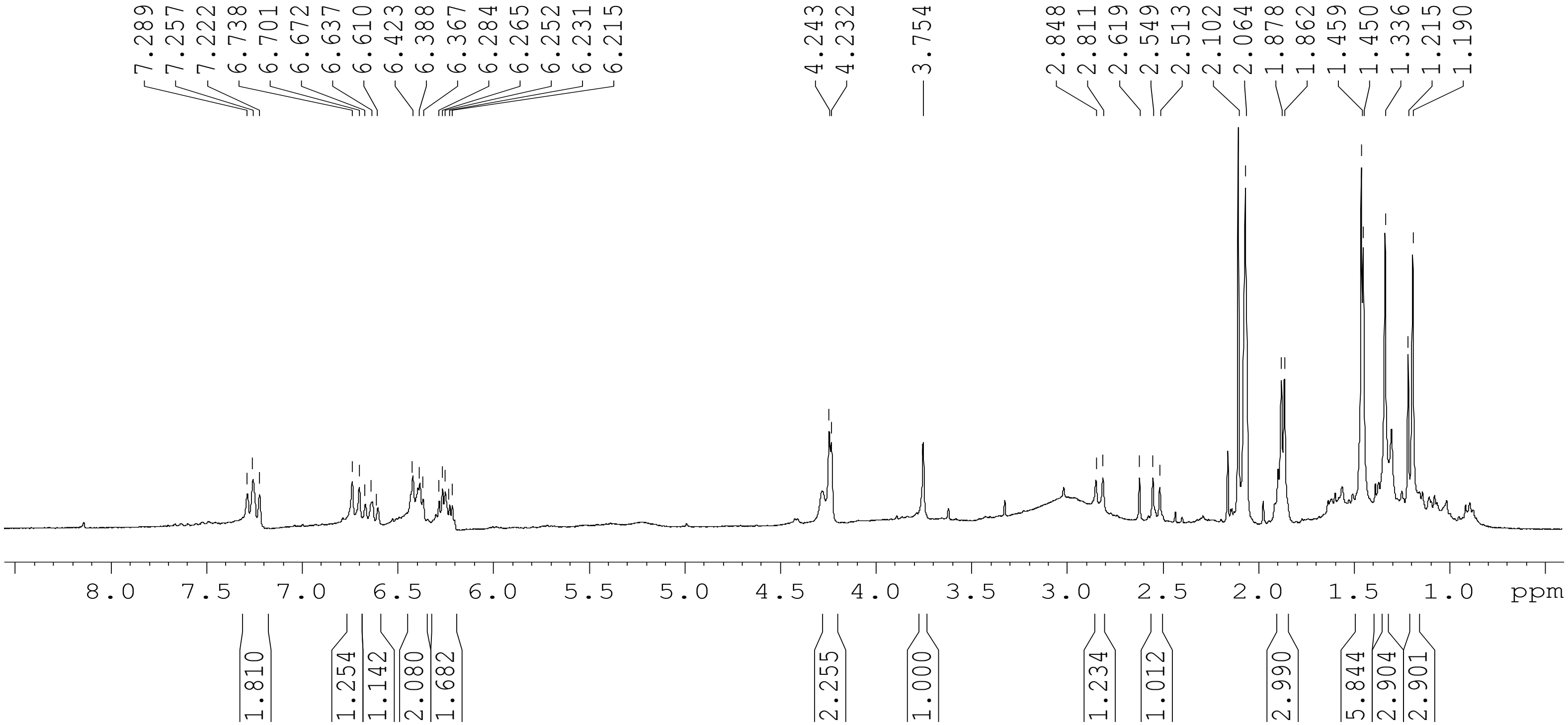

Figure 11. The ^{1}H NMR Spectrum of Compound **2** in in Acetone-d$_6$ (400 MHz)

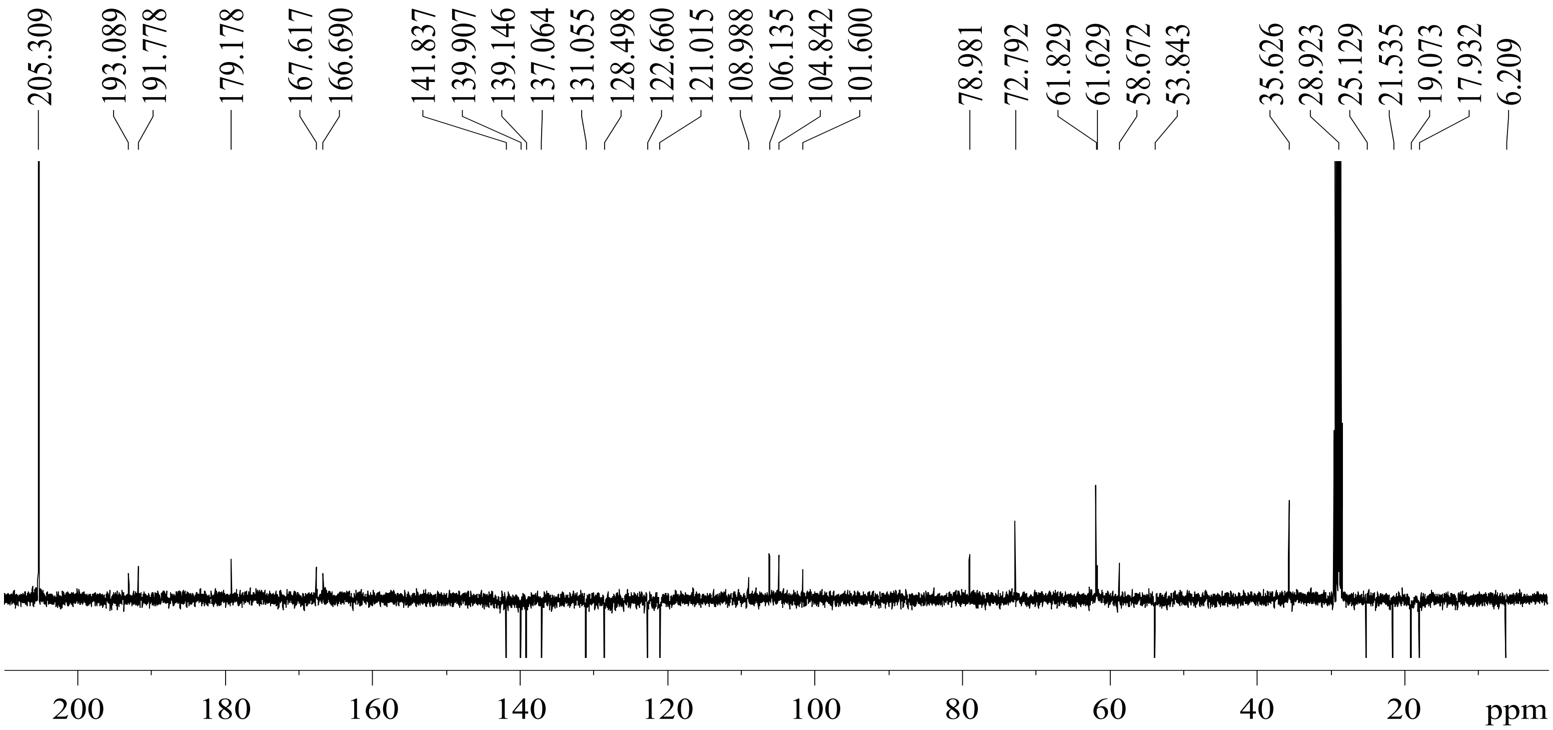

Figure 12. The ^{13}C APT NMR Spectrum of Compound **2** in Acetone-d_6 (100 MHz)

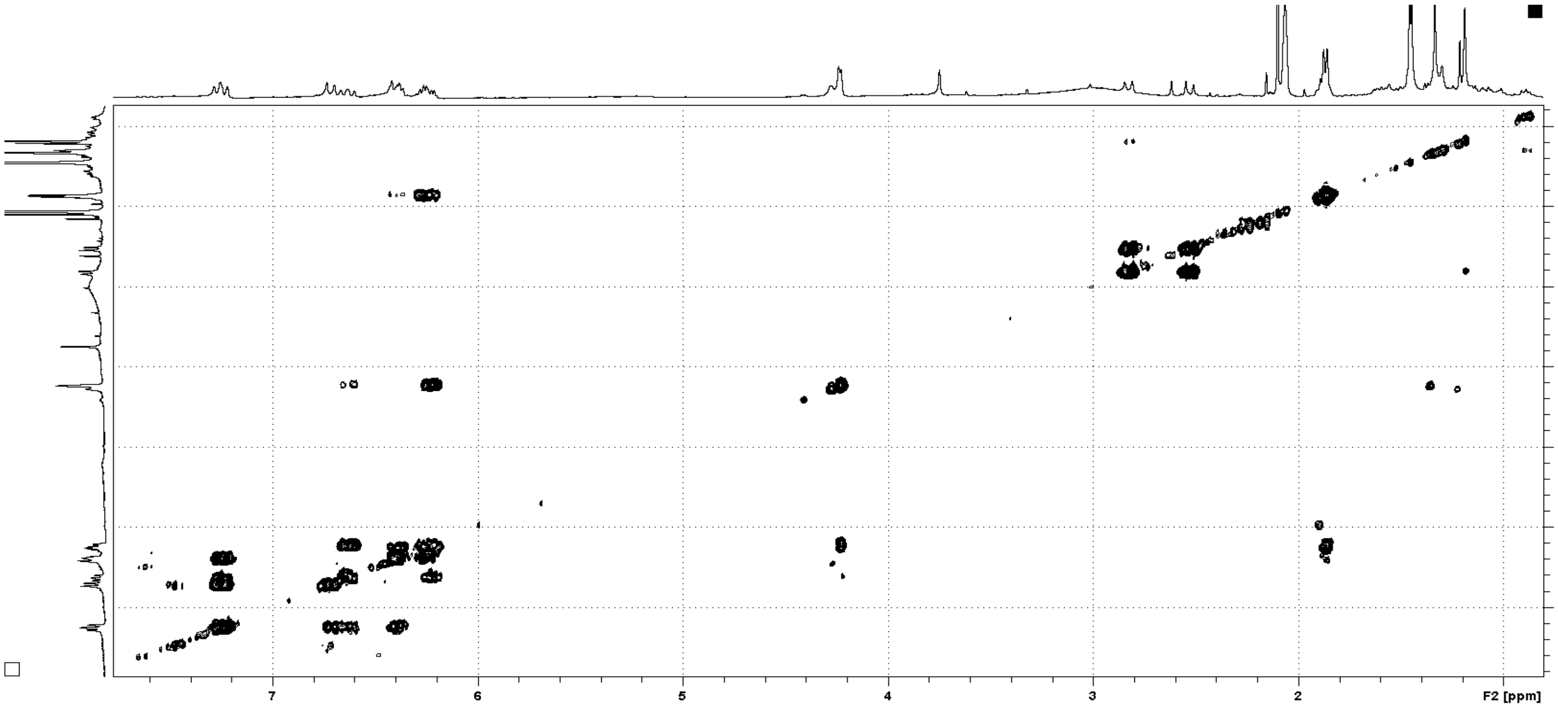

Figure 13. The ^{1}H-^{1}H COSY Spectrum of Compound **2** in Acetone-d_6 (400 MHz)

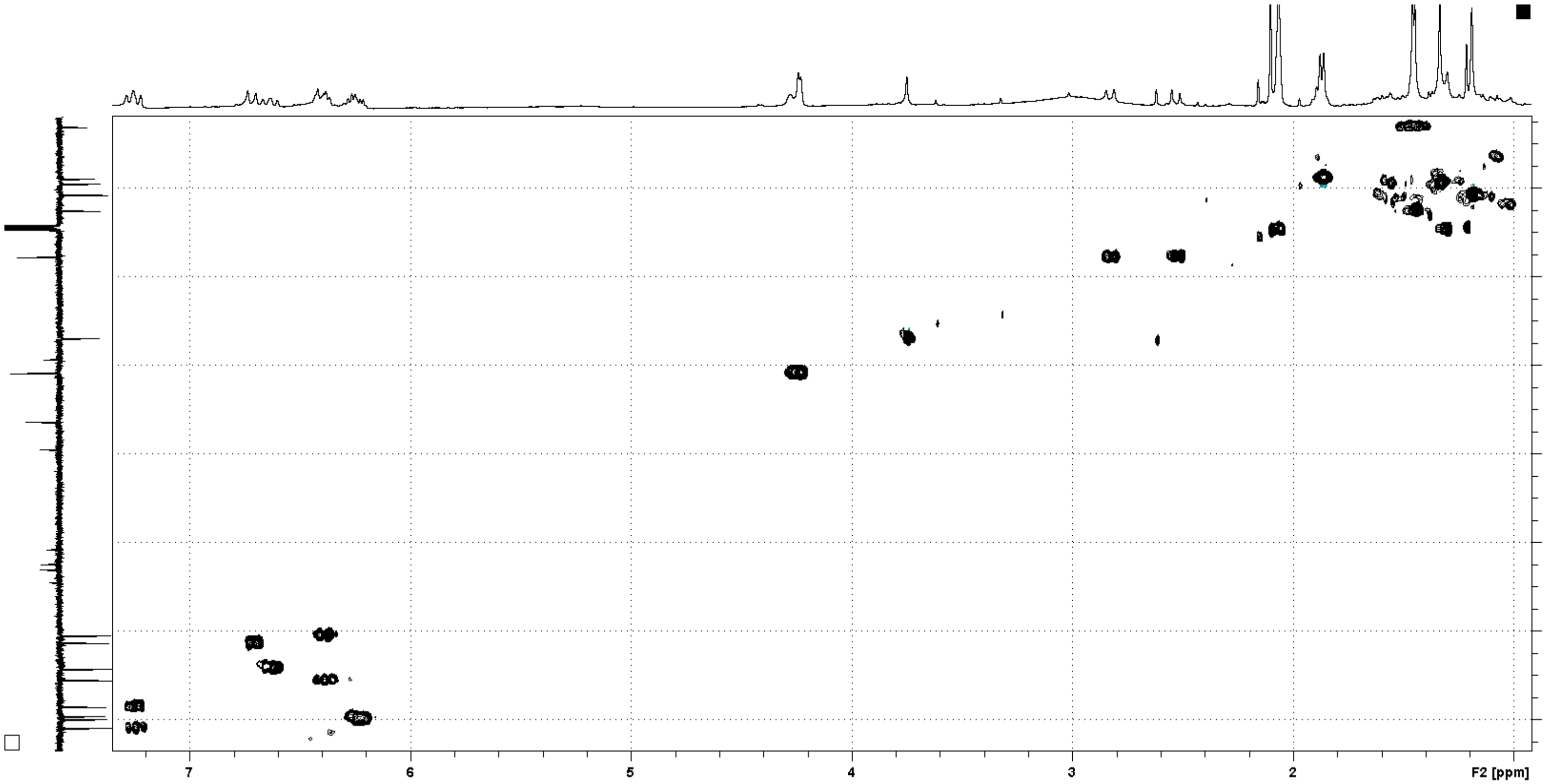

Figure 14. The HSQC Spectrum of Compound **2** in Acetone-d_6 (400 MHz for ^1H)

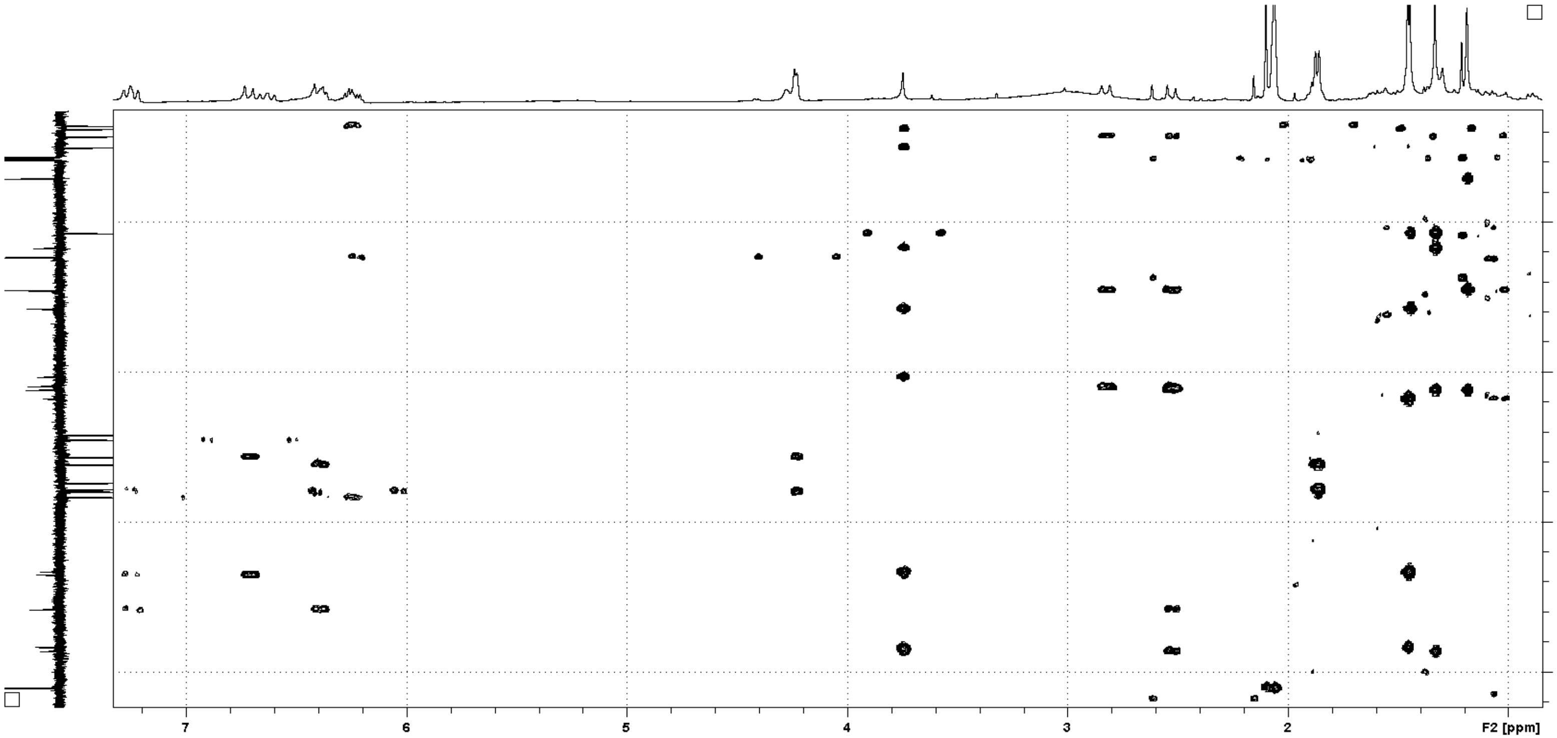

Figure 15. The HMBC Spectrum of Compound **2** in Acetone-d_6 (400 MHz for ^1H)

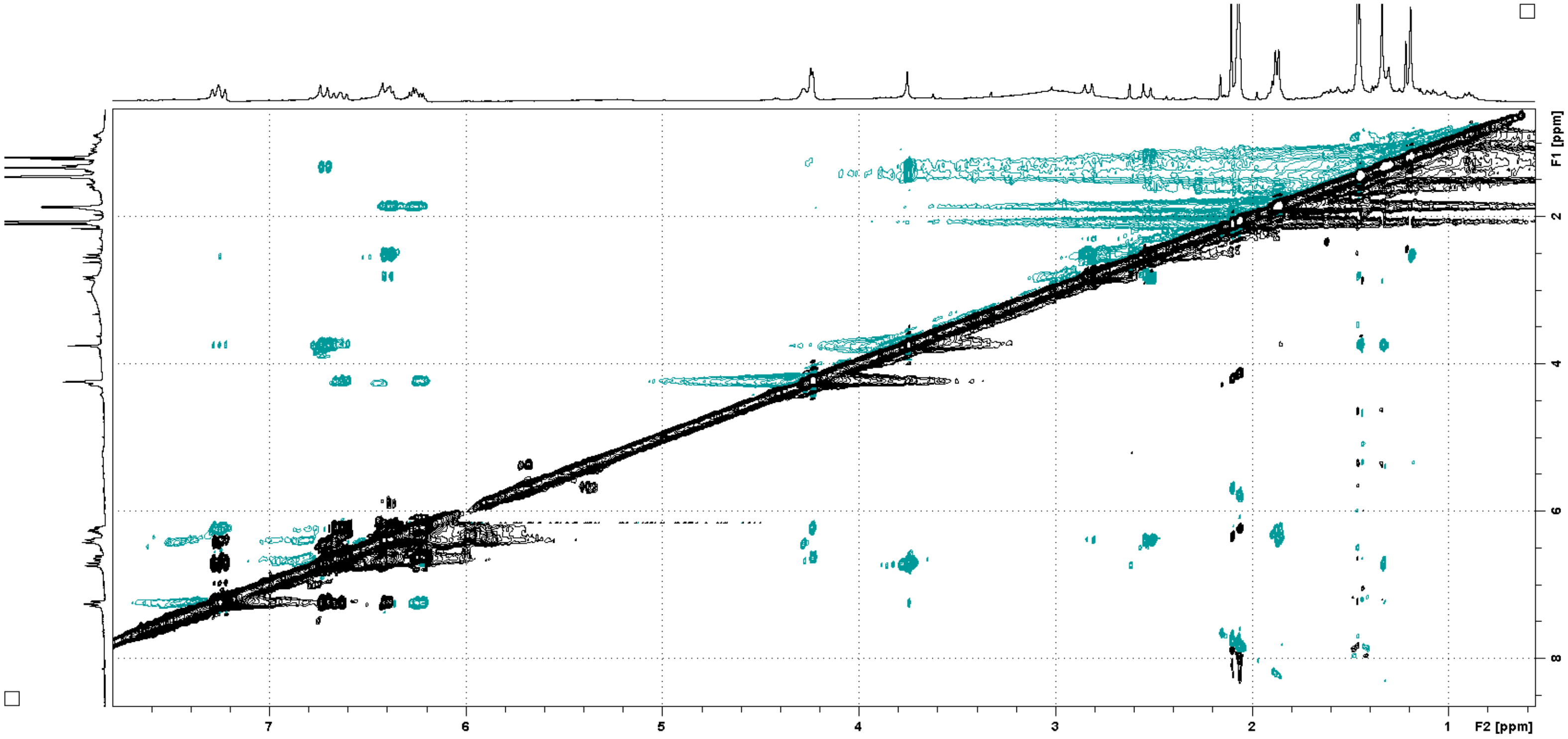

Figure 16. The ROESY Spectrum of Compound **2** in Acetone-d_6 (400 MHz)

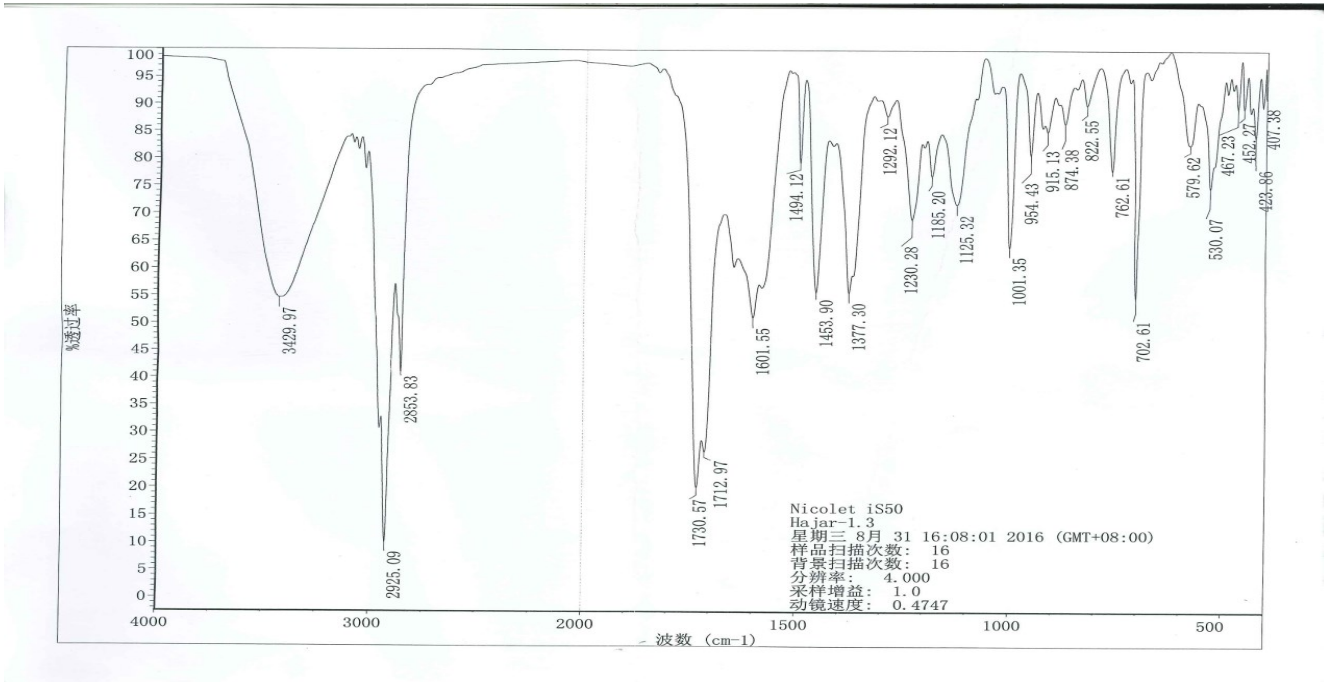

Figure 17. The IR Spectrum of Compound **3**

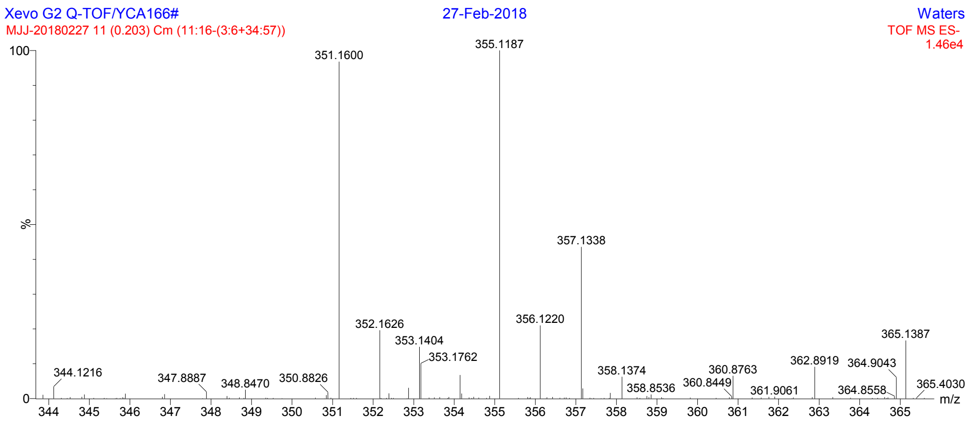

Figure 18. The HRESIMS Data of Compound **3**

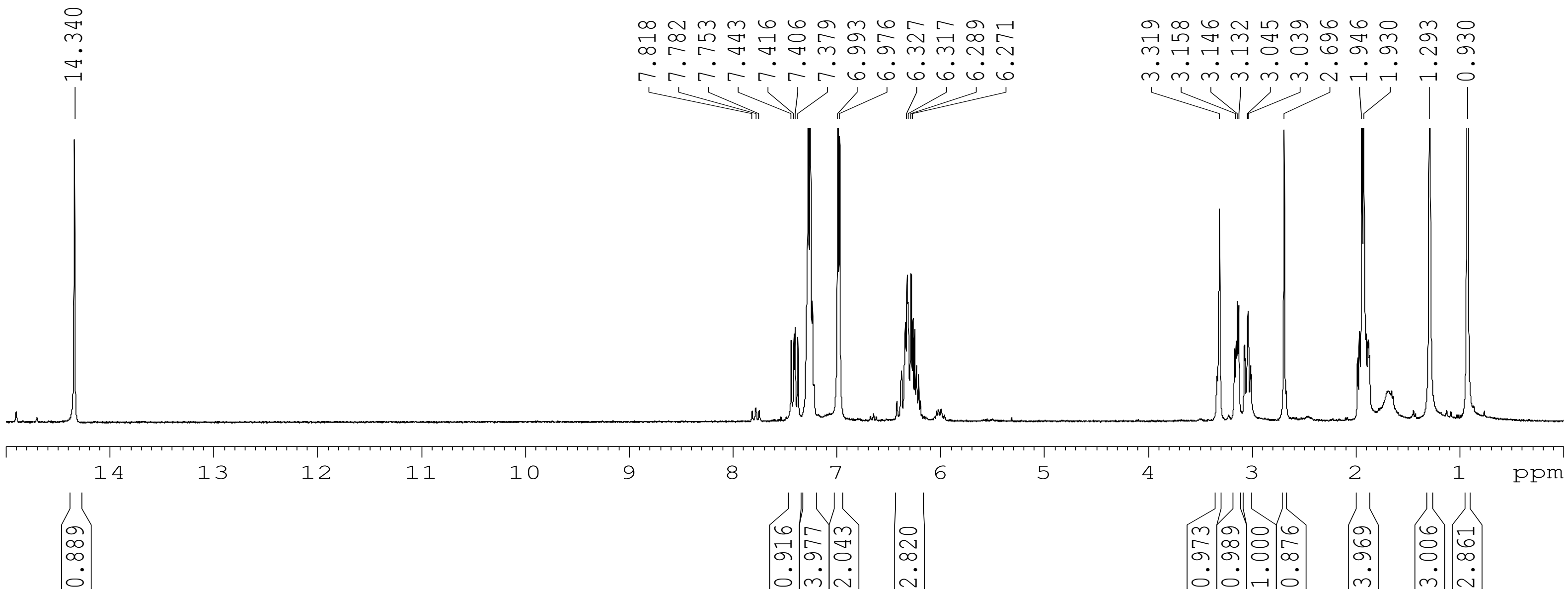

Figure 19. The ^{1}H NMR Spectrum of Compound **3** in $CDCl_3$ (400 MHz)

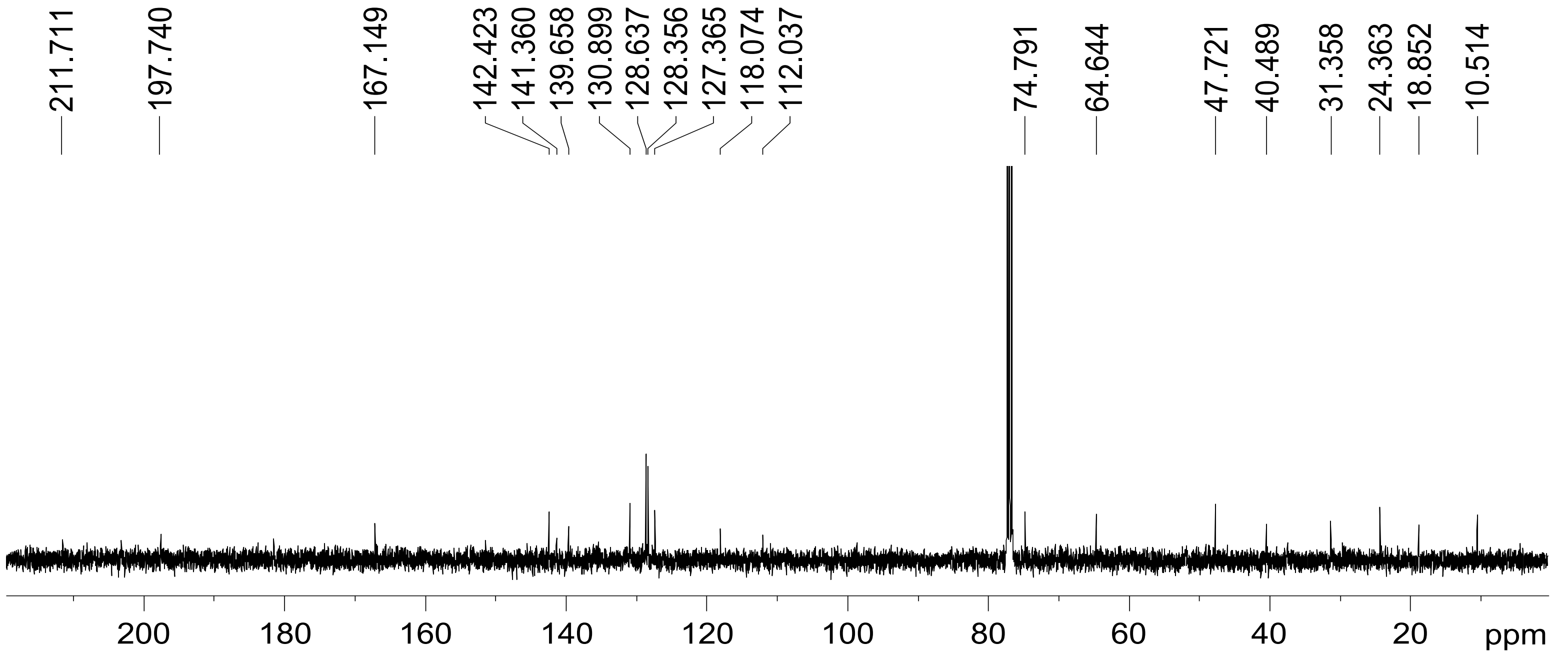

Figure 20. The ^{13}C CPD NMR Spectrum of Compound **3** in $CDCl_3$ (100 MHz)

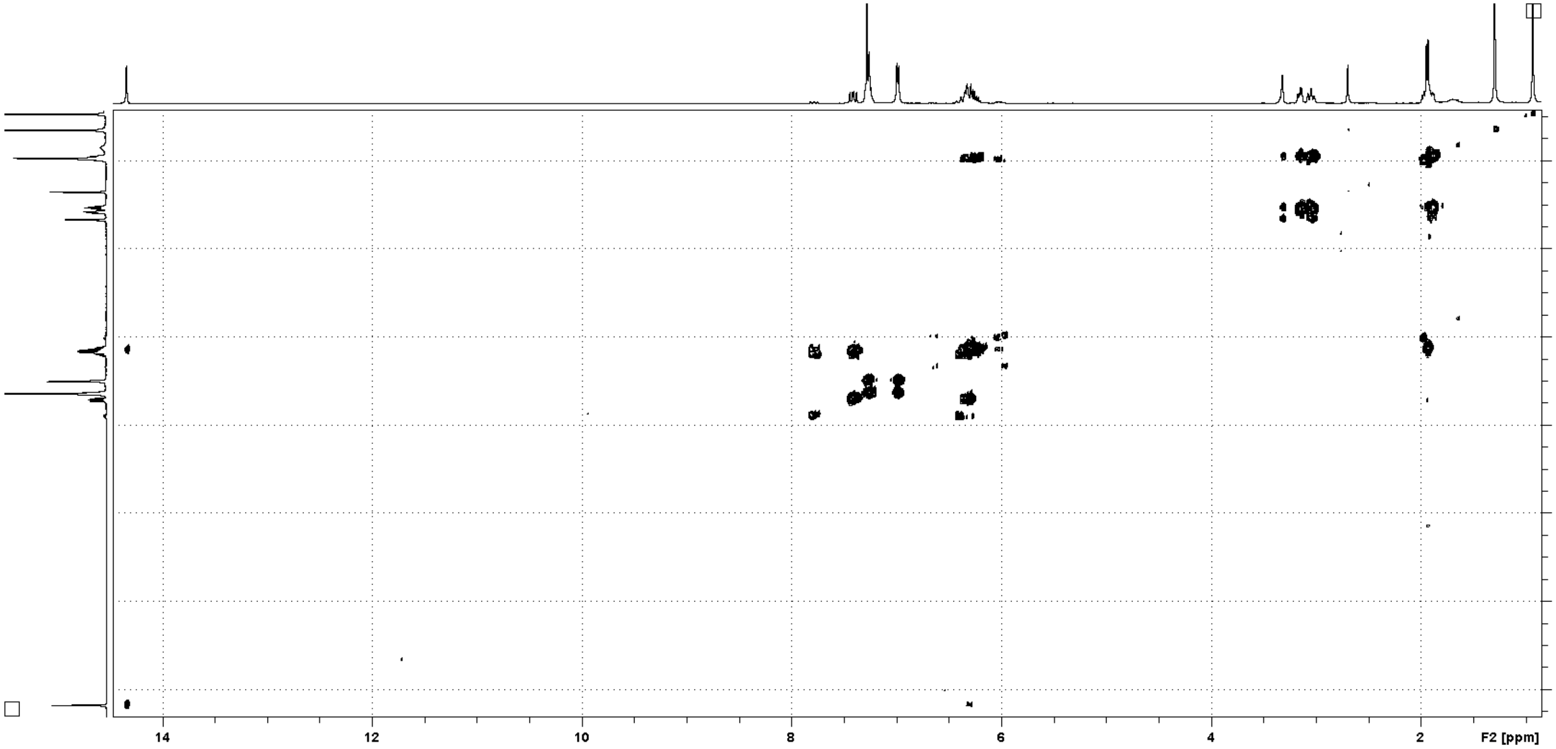

Figure 21. The ^{1}H-^{1}H COSY Spectrum of Compound **3** in $CDCl_3$ (400 MHz)

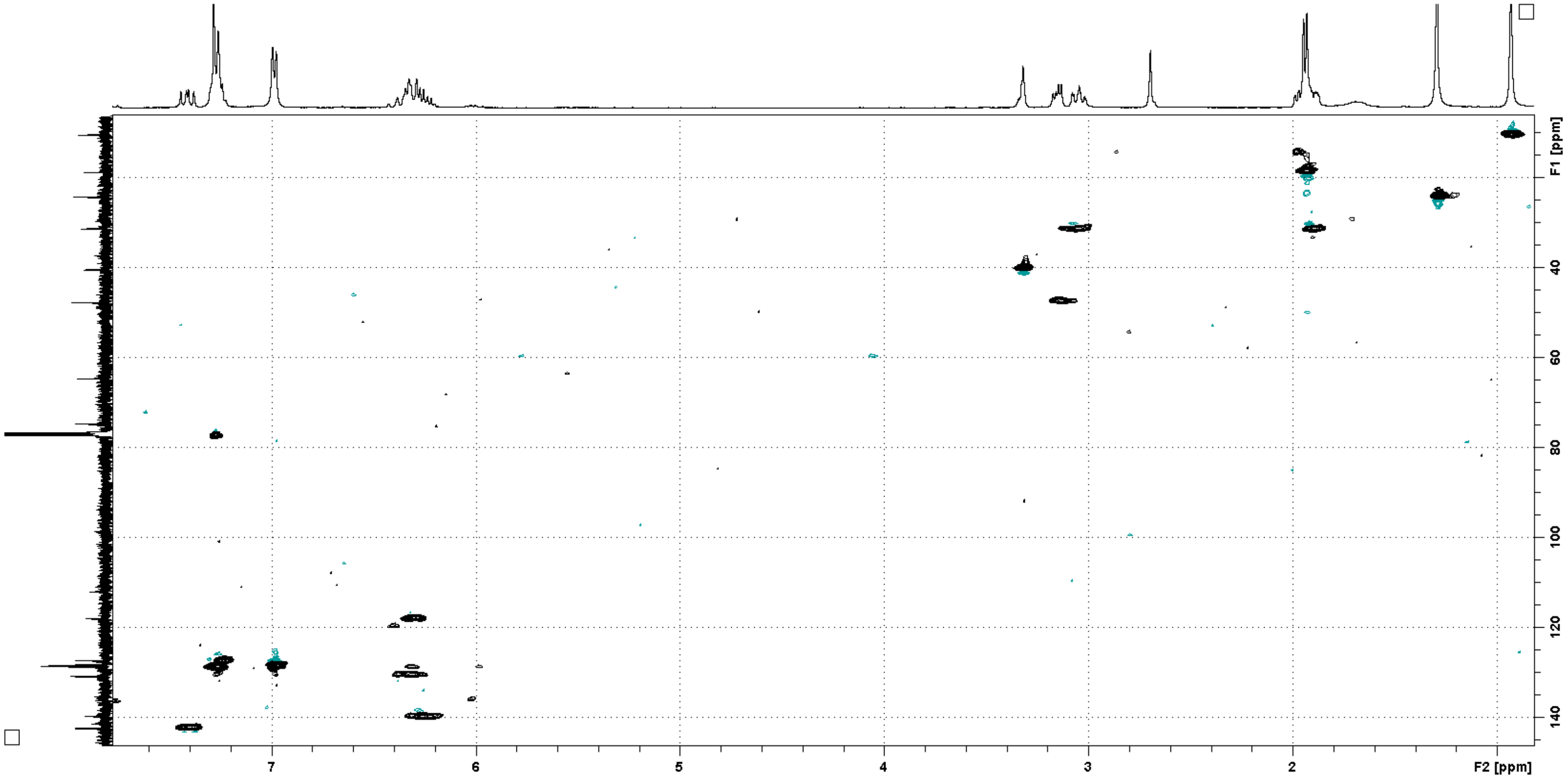

Figure 22. The HSQC Spectrum of Compound **3** in $CDCl_3$ (400 MHz for ^{1}H)

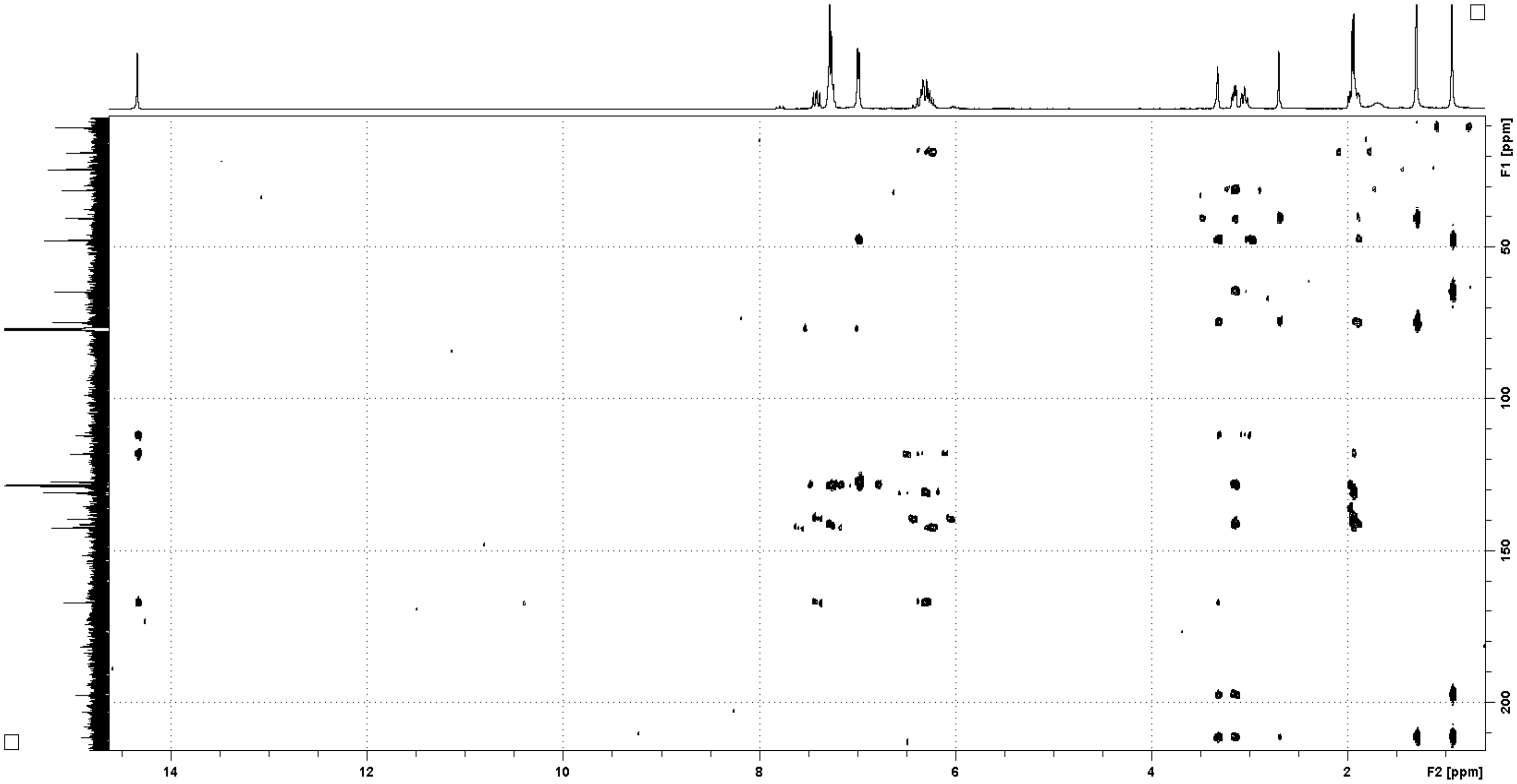

Figure 23. The HMBC Spectrum of Compound **3** in $CDCl_3$(400 MHz for ^1H)

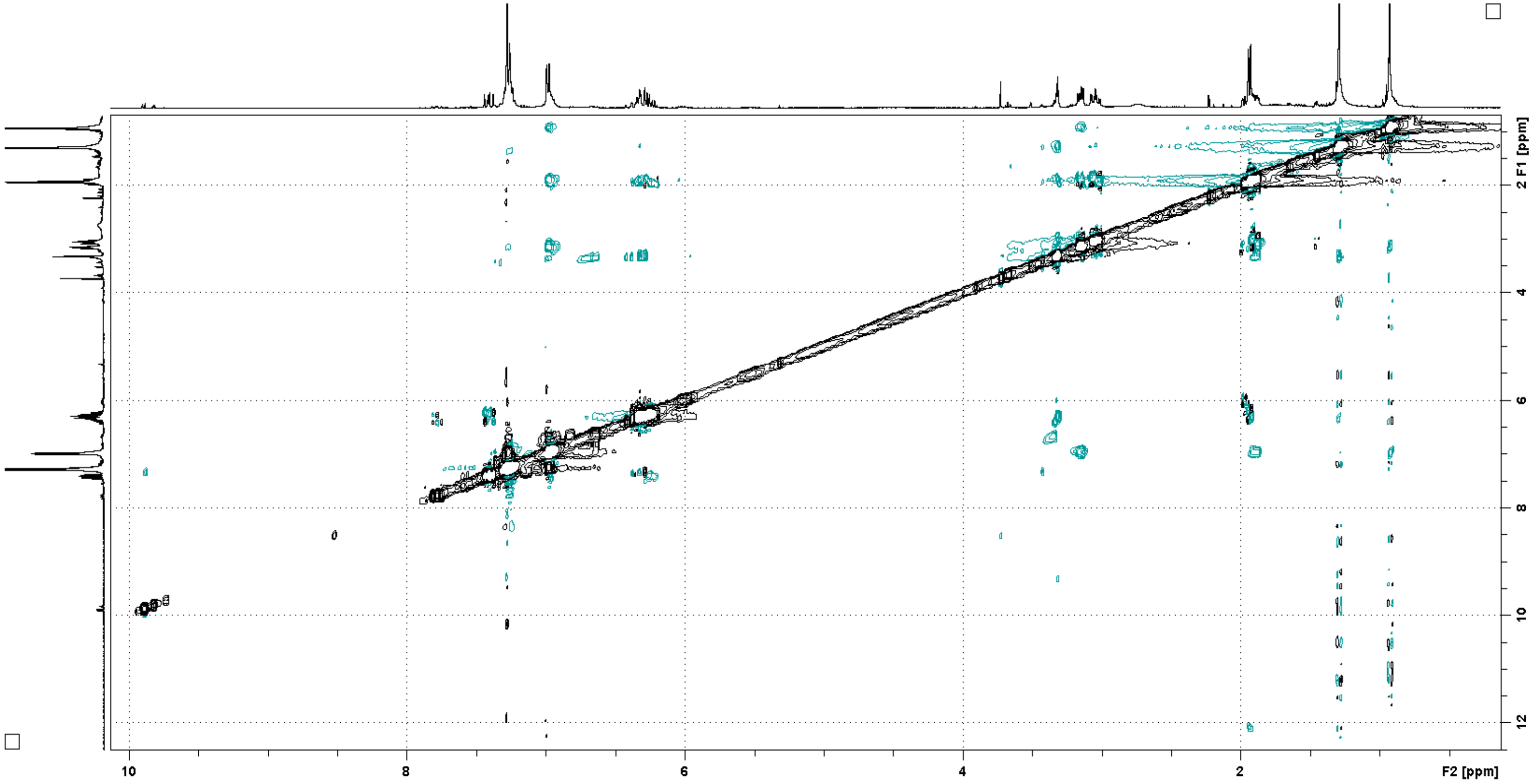

Figure 24. The ROESY Spectrum of Compound **3** in $CDCl_3$ (400 MHz)

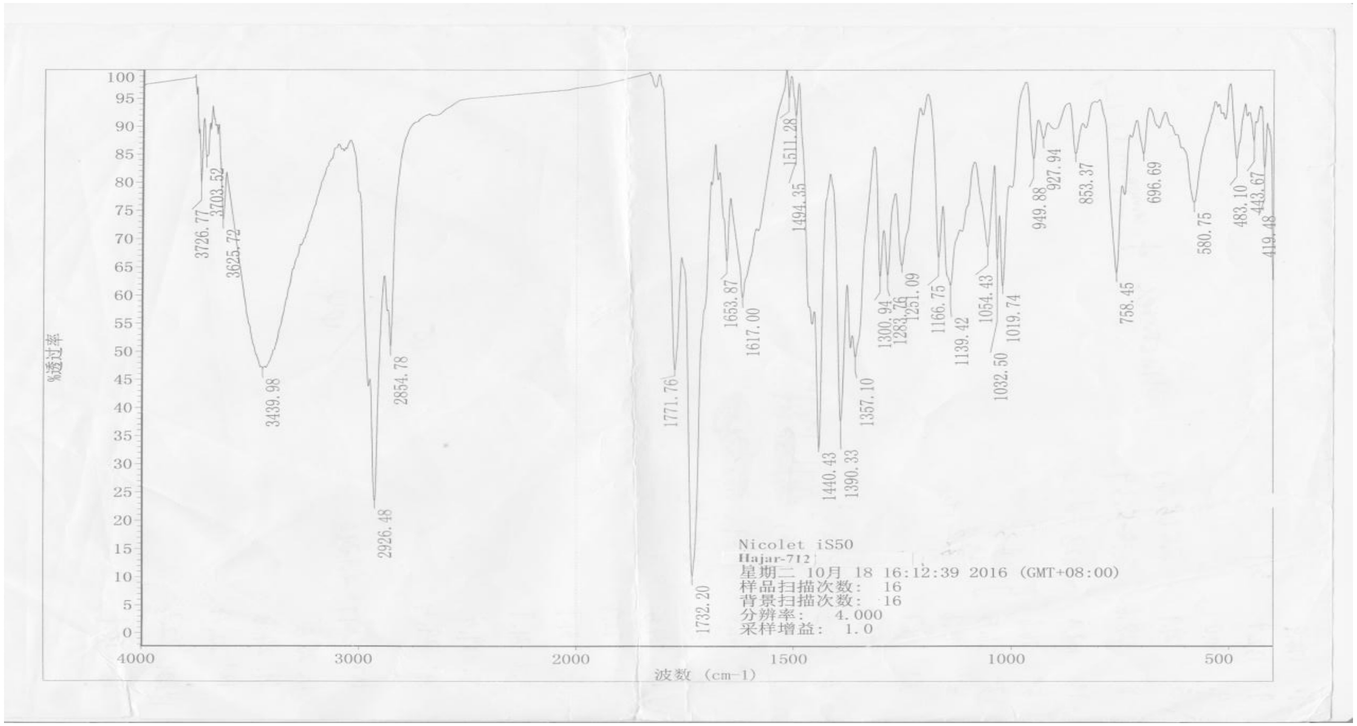

Figure 25. The IR Spectrum of Compound **4**

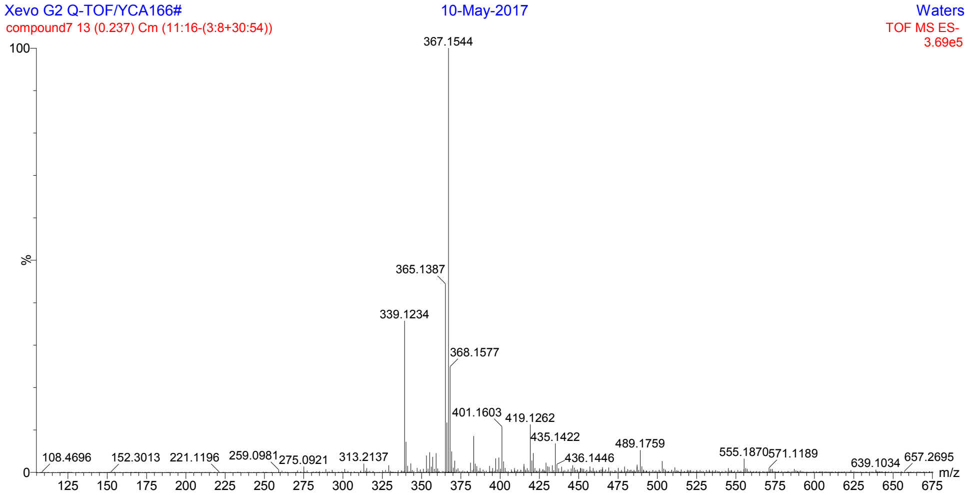

Figure 26. The HRESIMS Data of Compound **4**

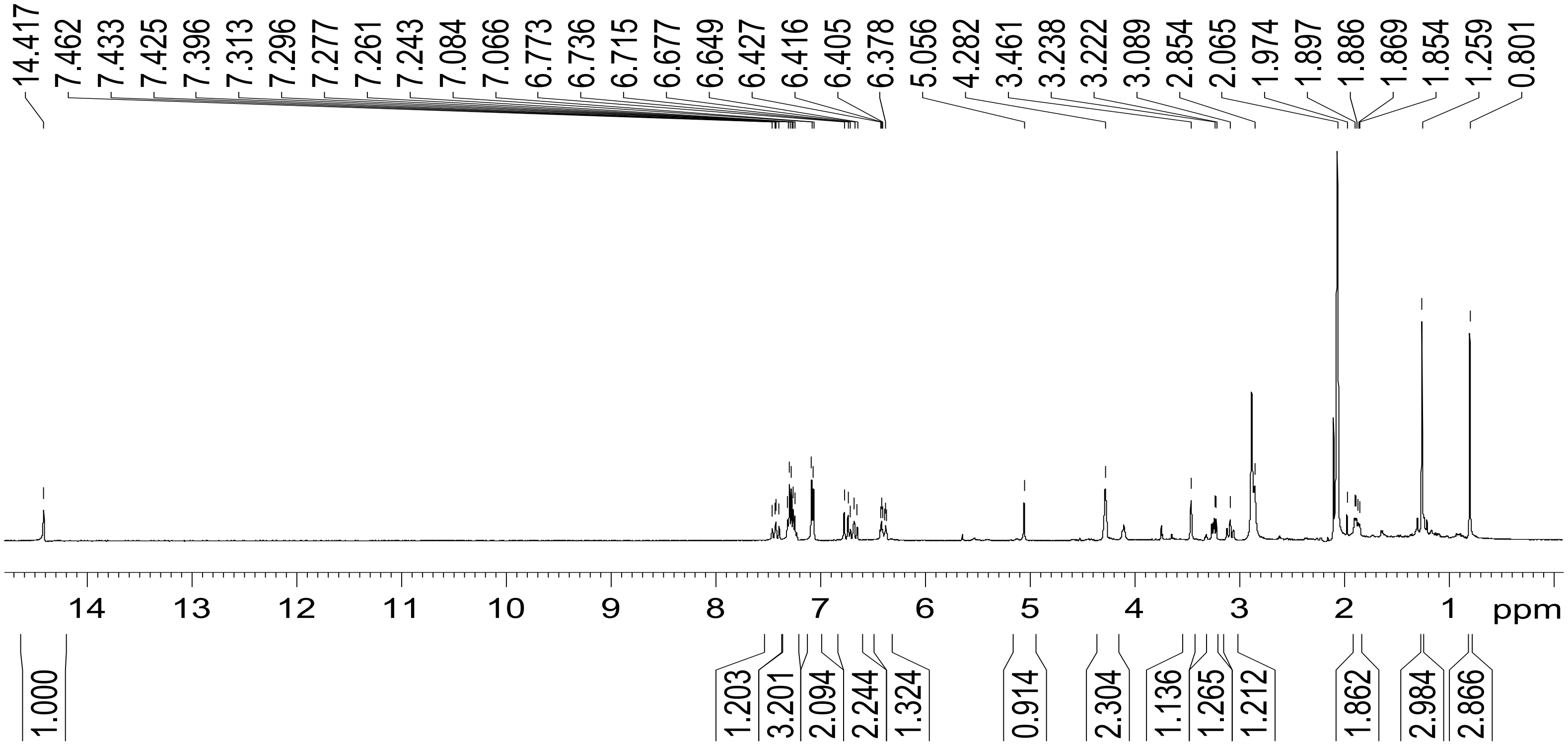

1.

Figure 27. The ^{1}H NMR Spectrum of Compound **4** in Acetone-d_6 (400 MHz)

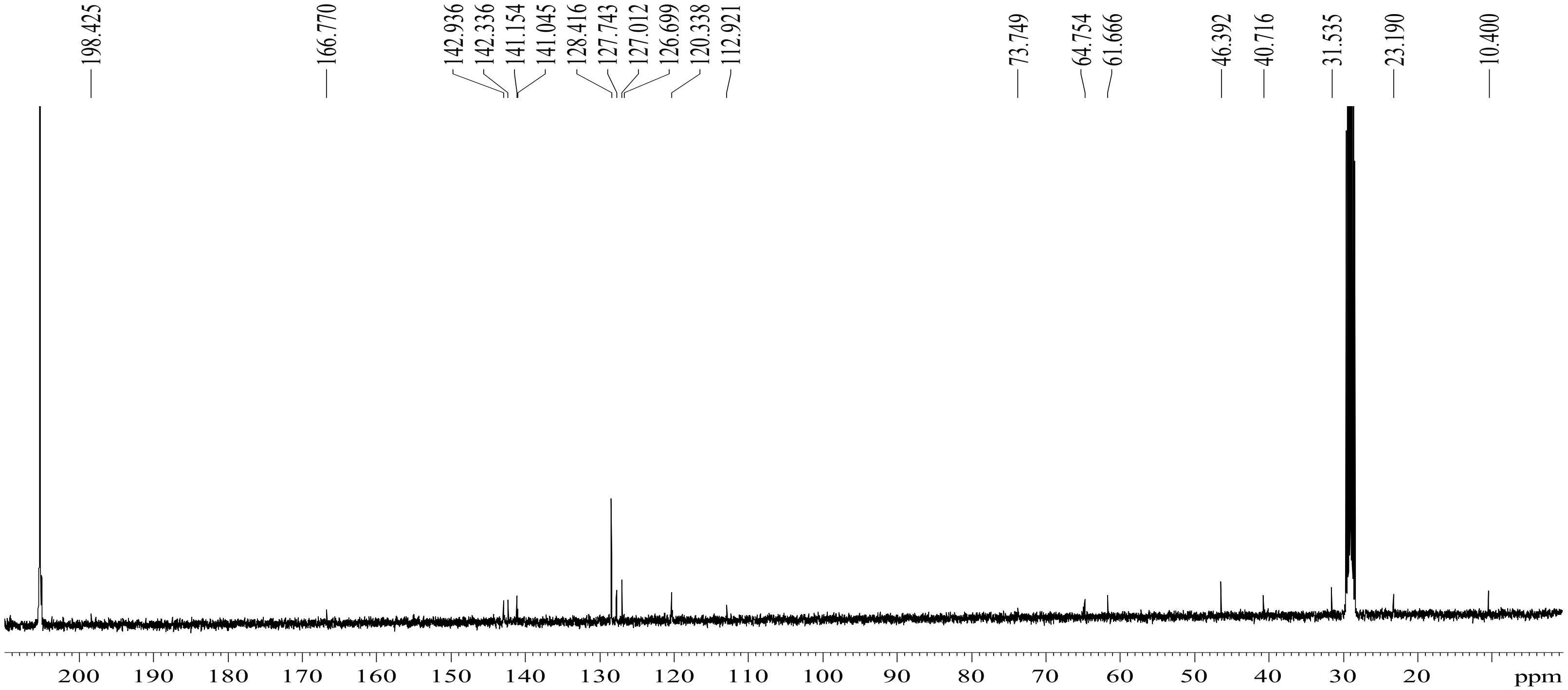

Figure 28. The ^{13}C CPD NMR Spectrum of Compound **4** in Acetone-d_6 (100 MHz)

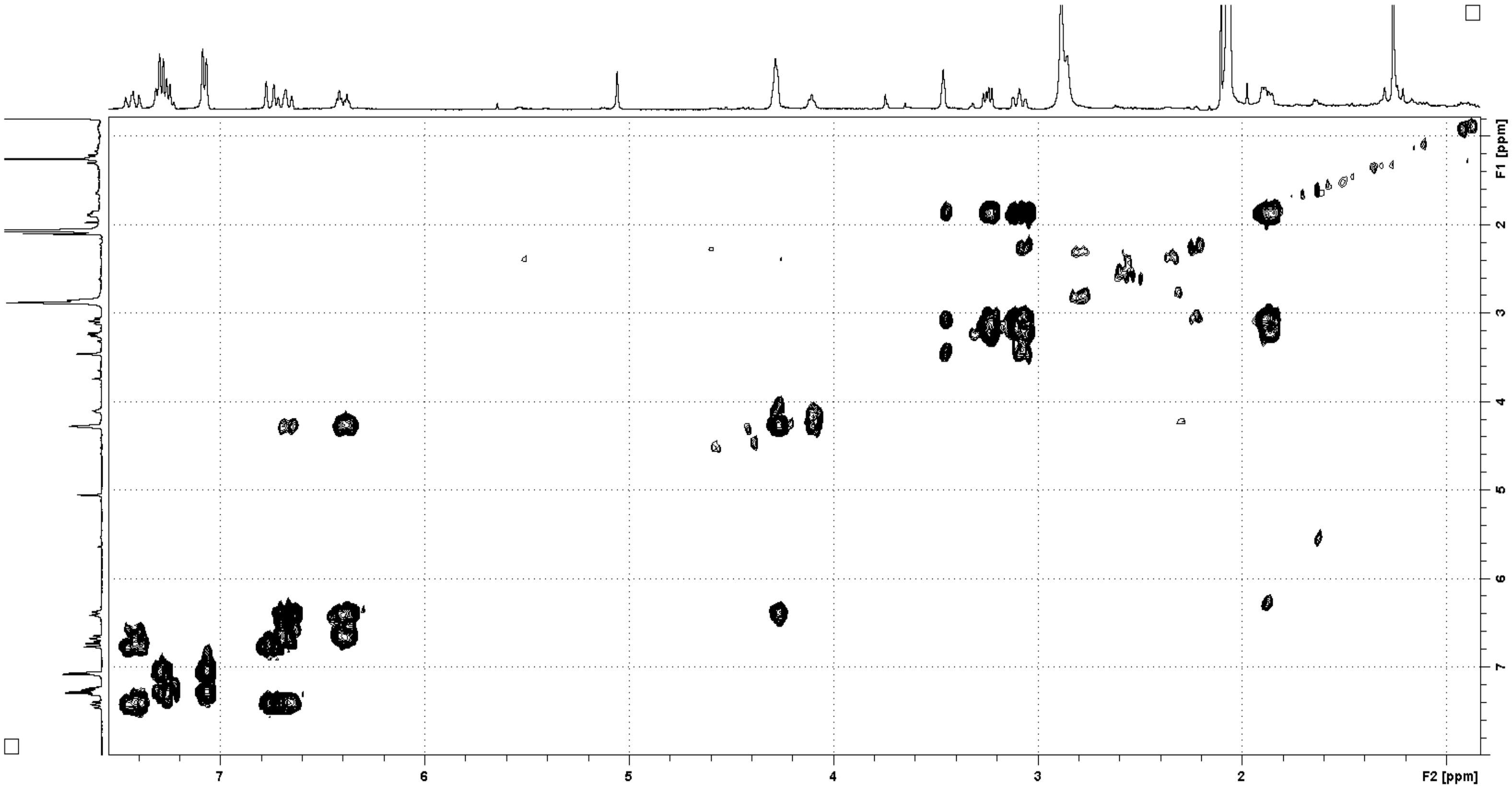

Figure 29. The ^{1}H-^{1}H COSY Spectrum of Compound **4** in Acetone-d_6 (400 MHz)

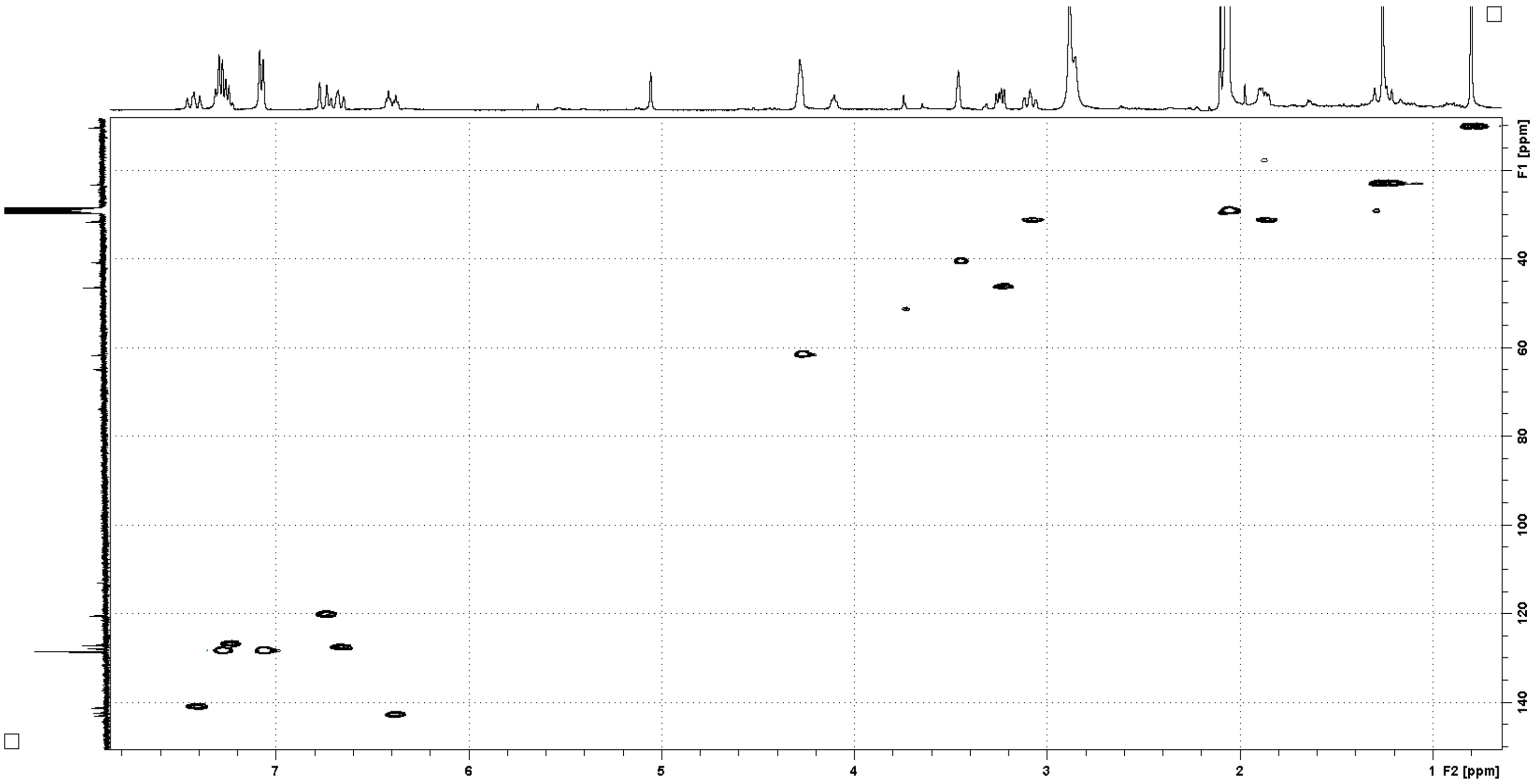

Figure 30. The HSQC Spectrum of Compound **4** in Acetone-d_6 (400 MHz for ^{1}H)

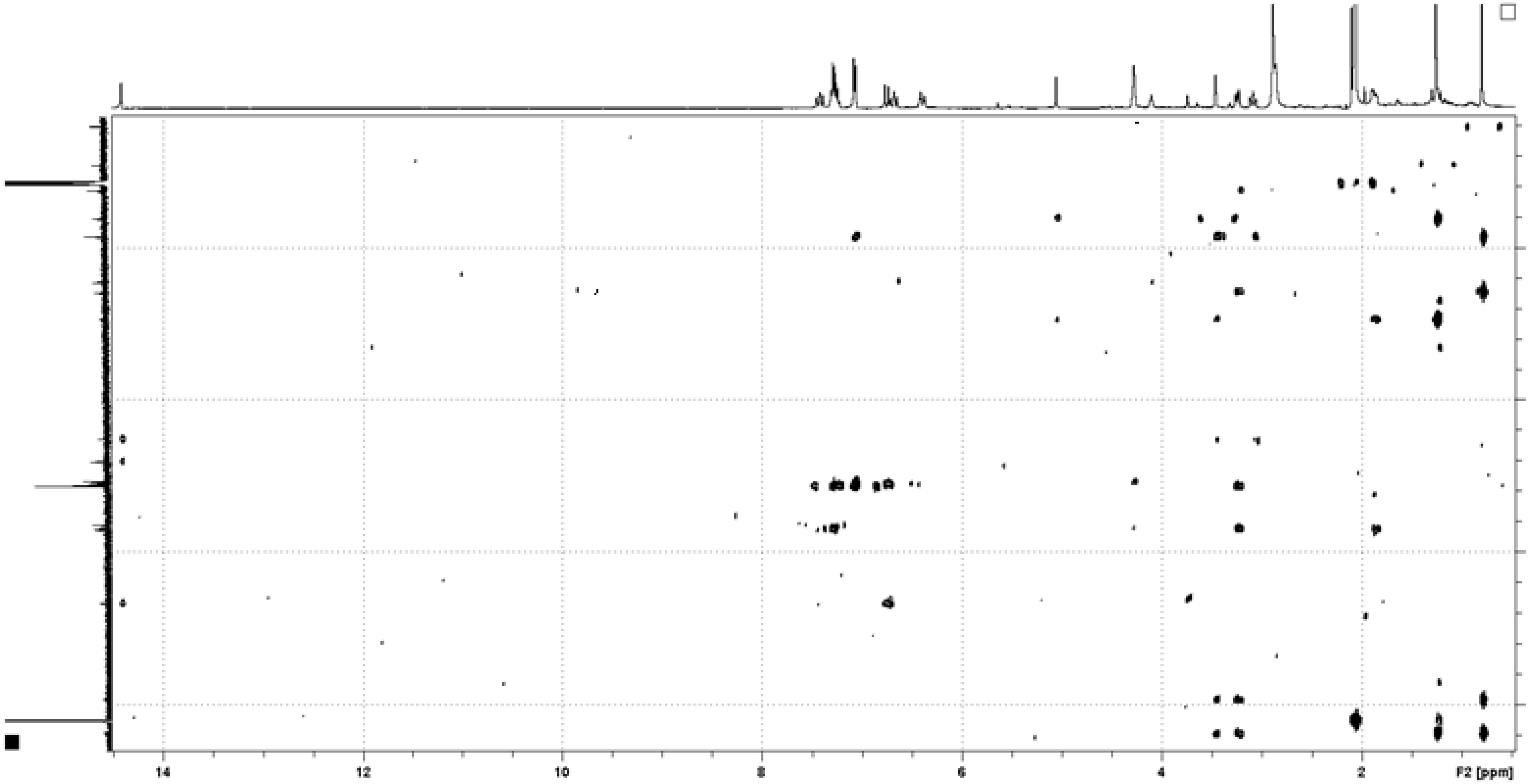

Figure 31. The HMBC Spectrum of Compound **4** in Acetone-d_6 (400 MHz for ^{1}H)

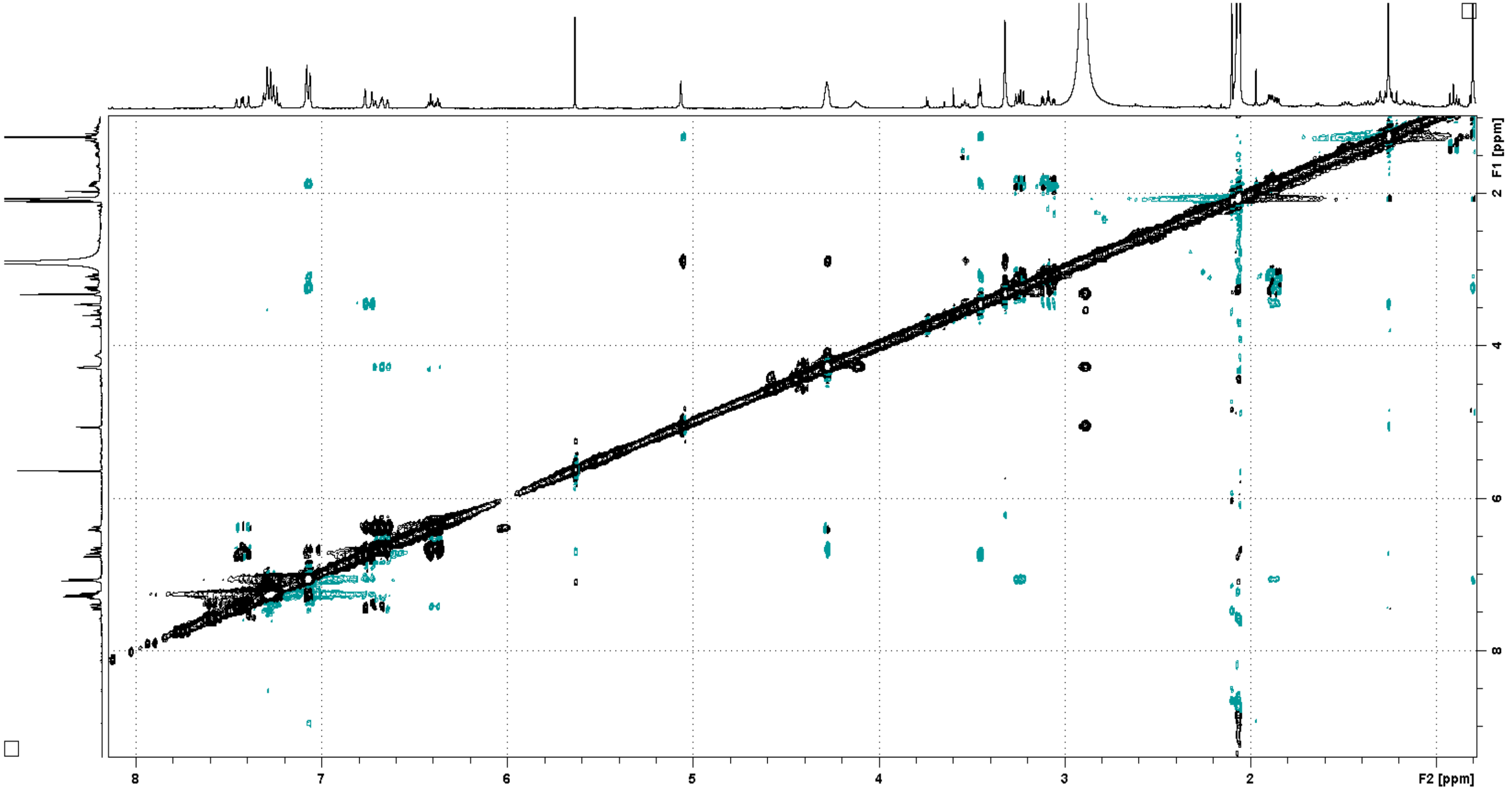

Figure 32. The ROESY Spectrum of Compound **4** in Acetone-d_6 (400 MHz)

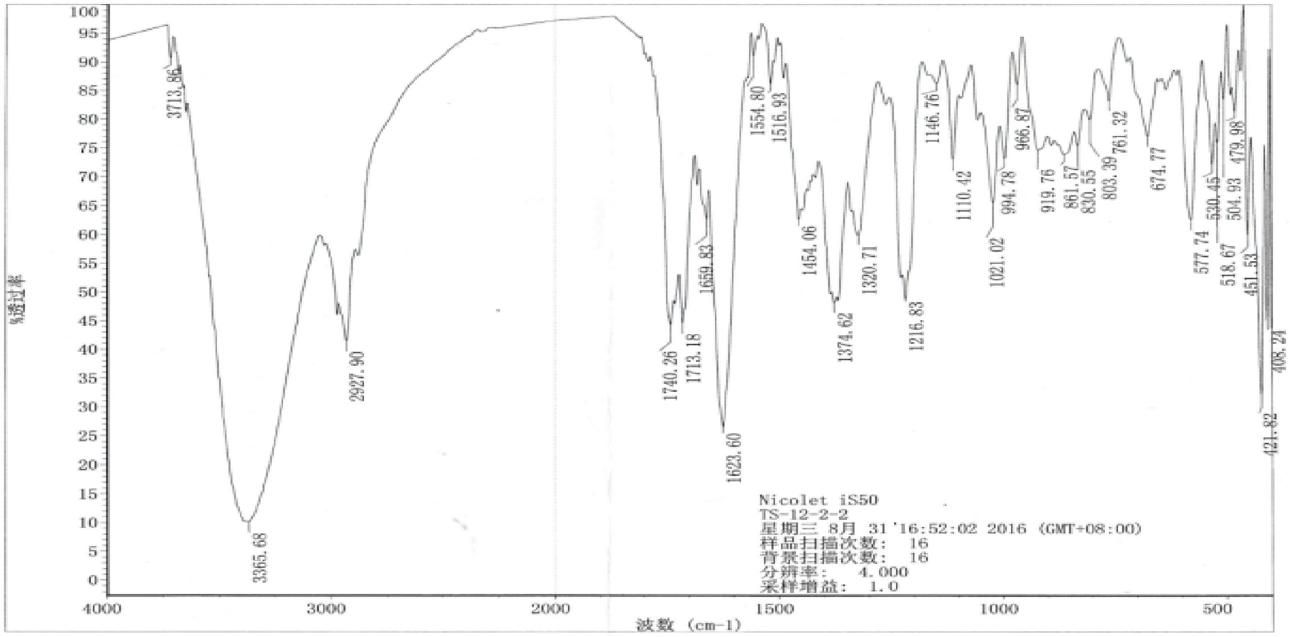

Figure 33. The IR Spectrum of Compound **5**

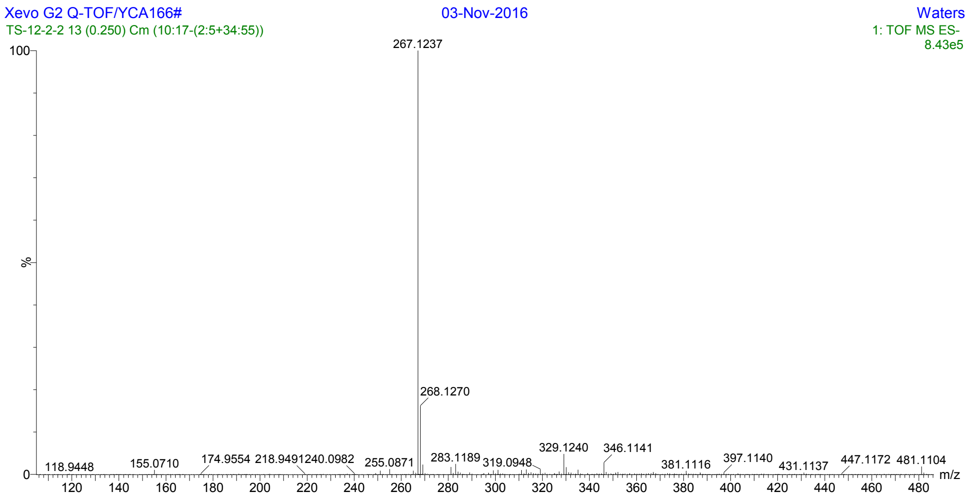

Figure 34. The HRESIMS Data of Compound **5**

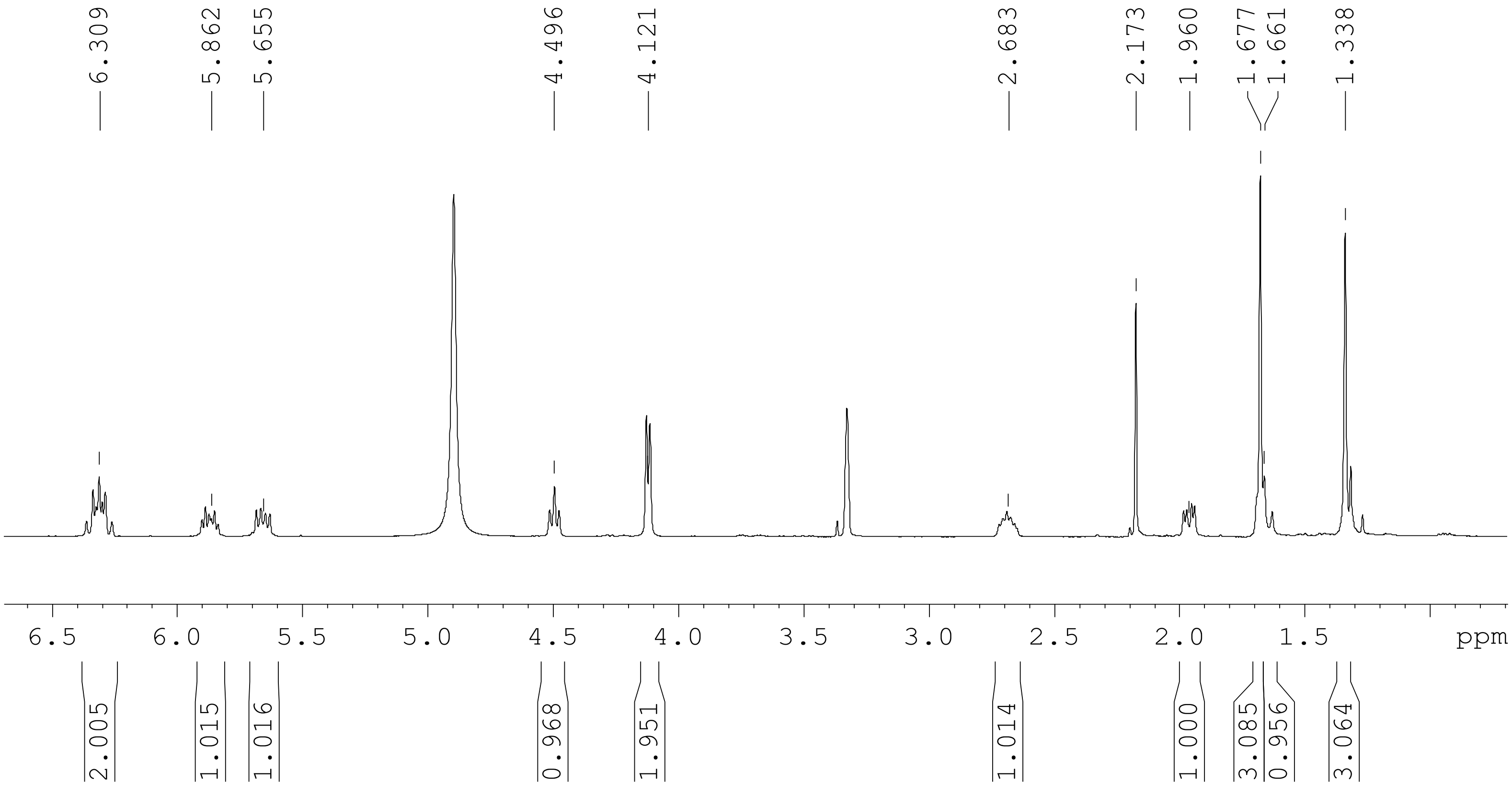

Figure 35. The ^{1}H NMR Spectrum of Compound **5** in CD_3OD (400 MHz)

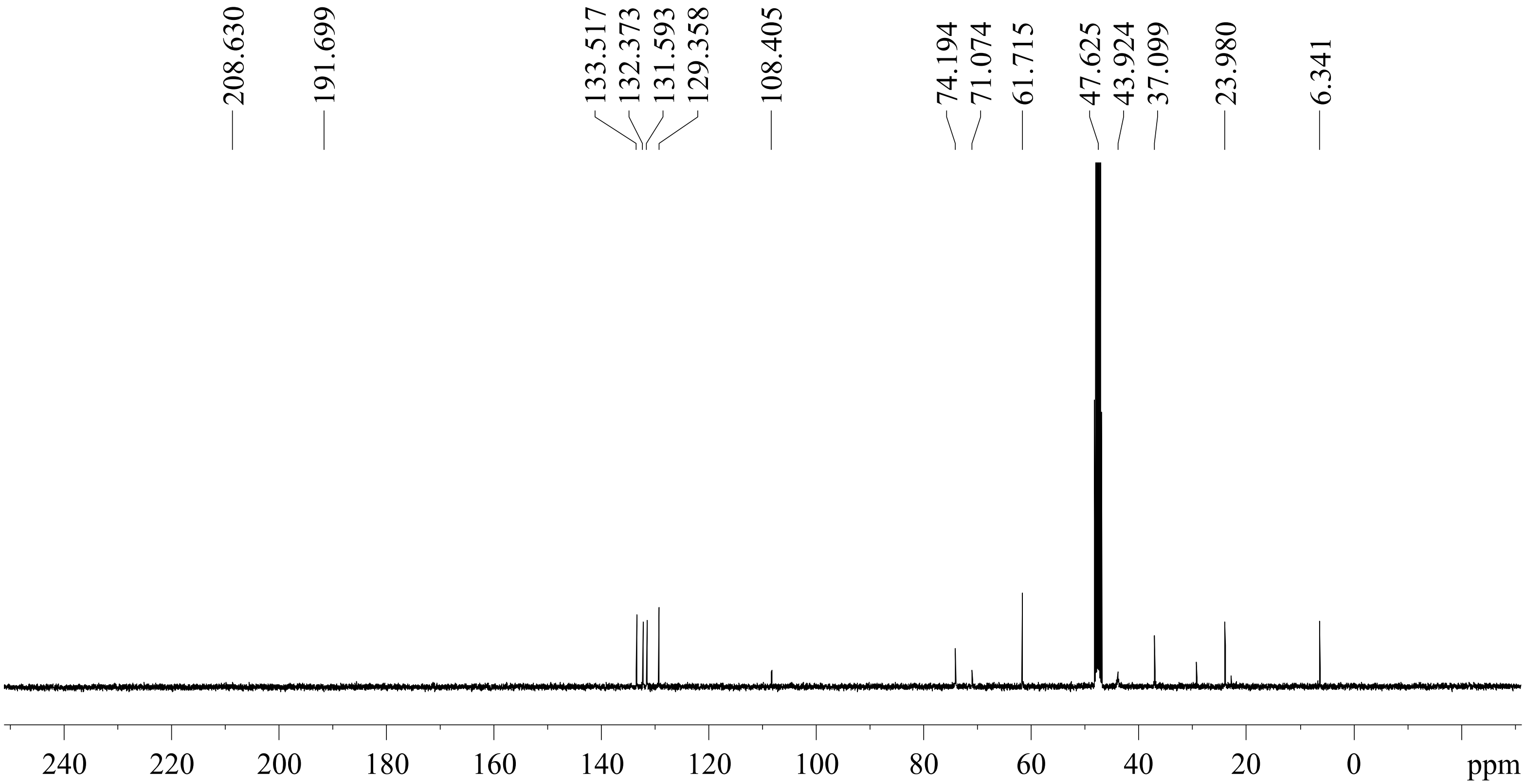

Figure 36. The ^{13}C CPD NMR Spectrum of Compound **5** in CD_3OD (100 MHz)

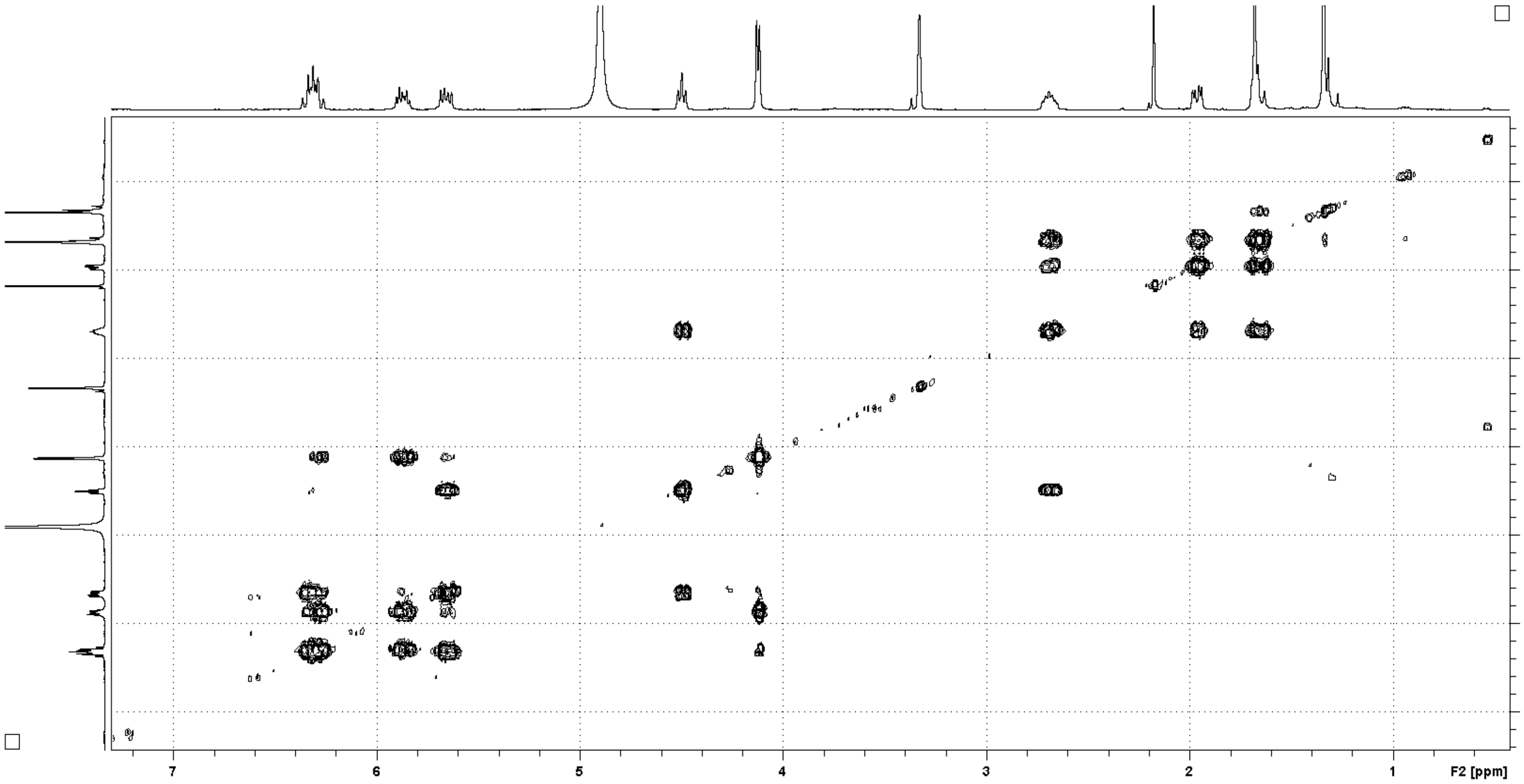

Figure 37. The ^{1}H-^{1}H COSY Spectrum of Compound **5** in CD_3OD (400 MHz)

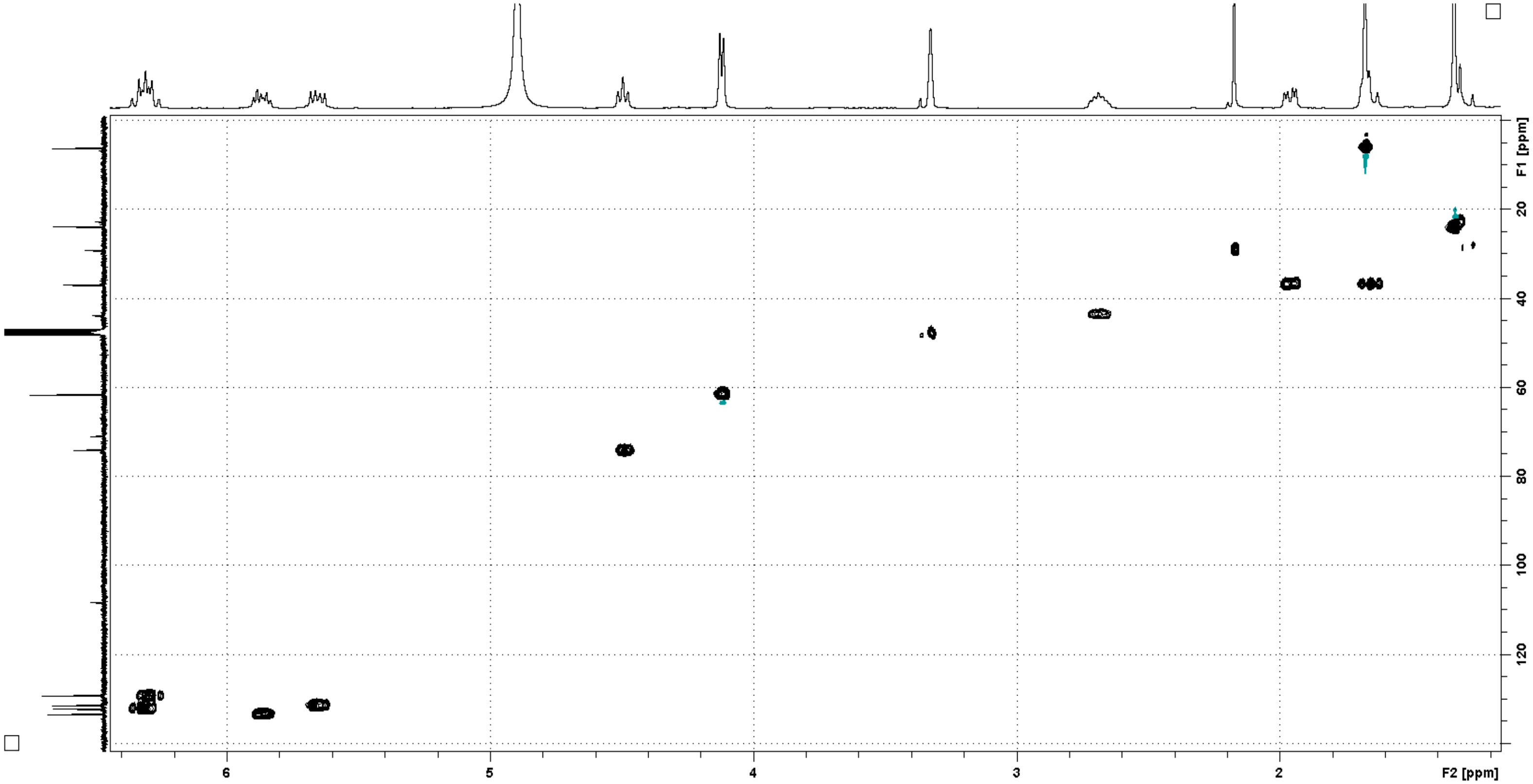

Figure 38. The HSQC Spectrum of Compound **5** in CD_3OD (400 MHz for ^1H)

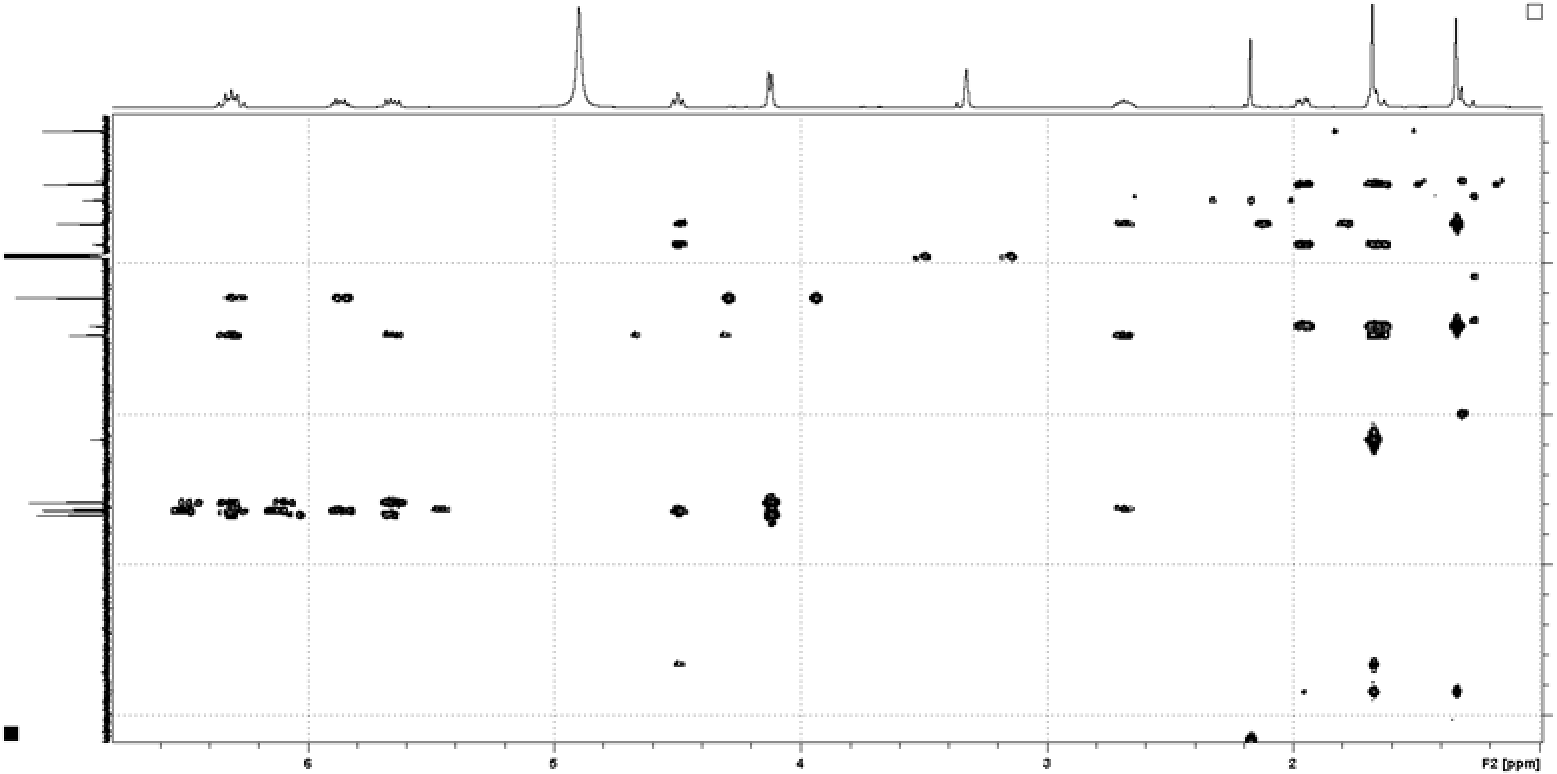

Figure 39. The HMBC Spectrum of Compound **5** in CD_3OD (400 MHz for ^1H)

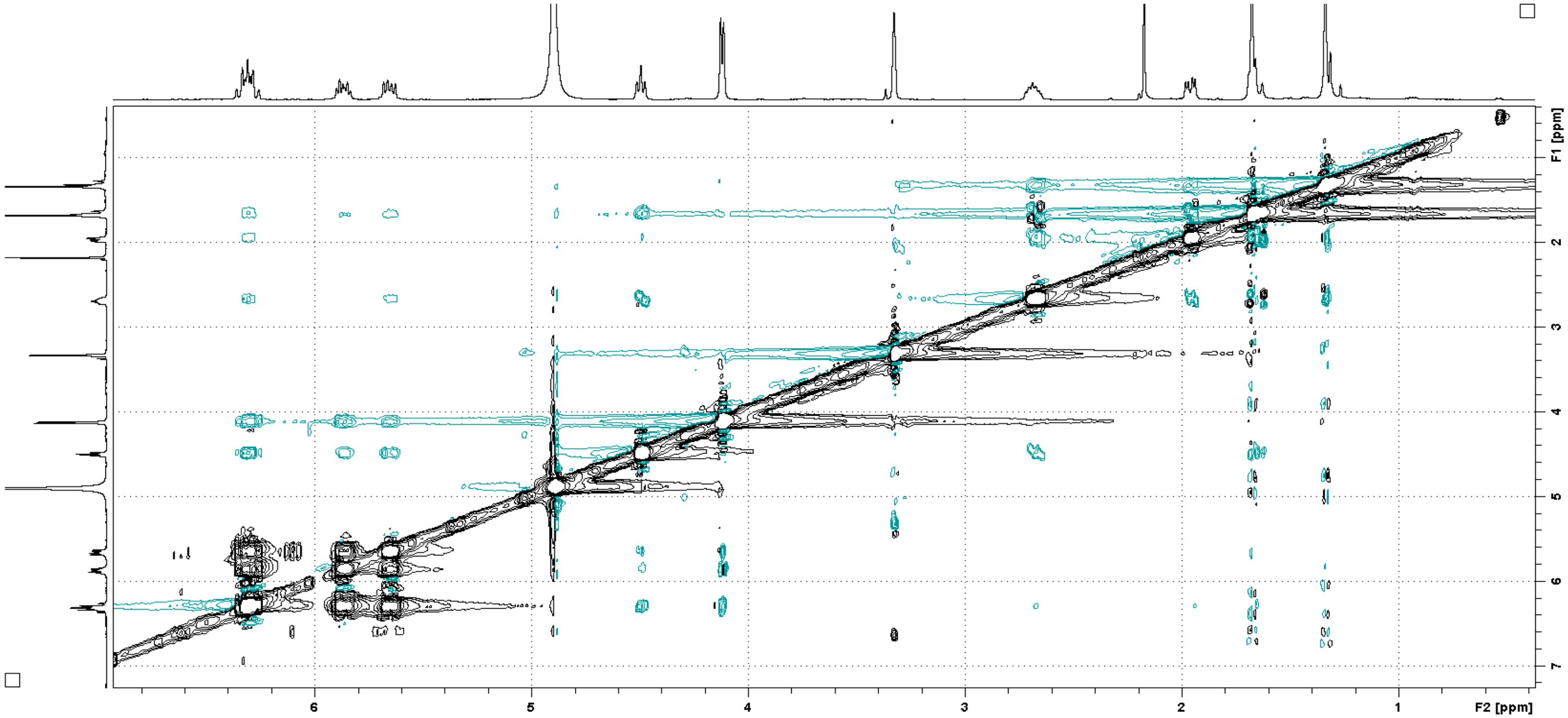

Figure 40. The ROESY Spectrum of Compound **5** in CD_3OD (400 MHz)

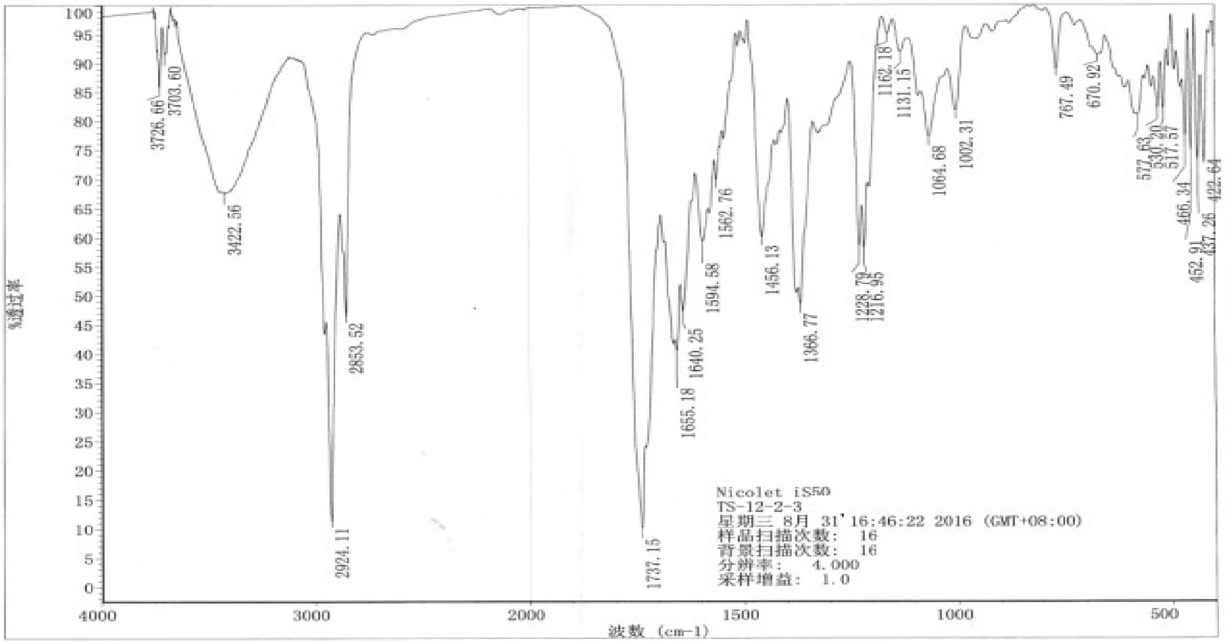

Figure 41. The IR Spectrum of Compound **6**

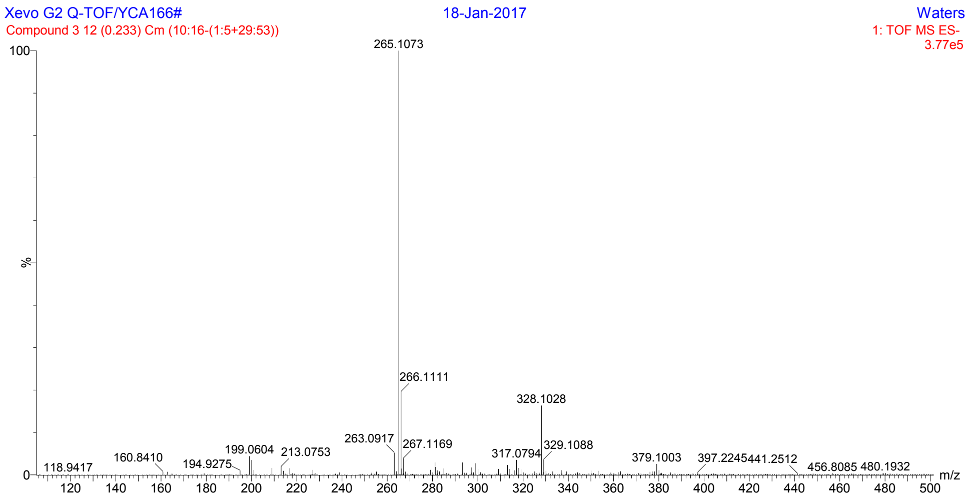

Figure 42. The HRESIMS Data of Compound **6**

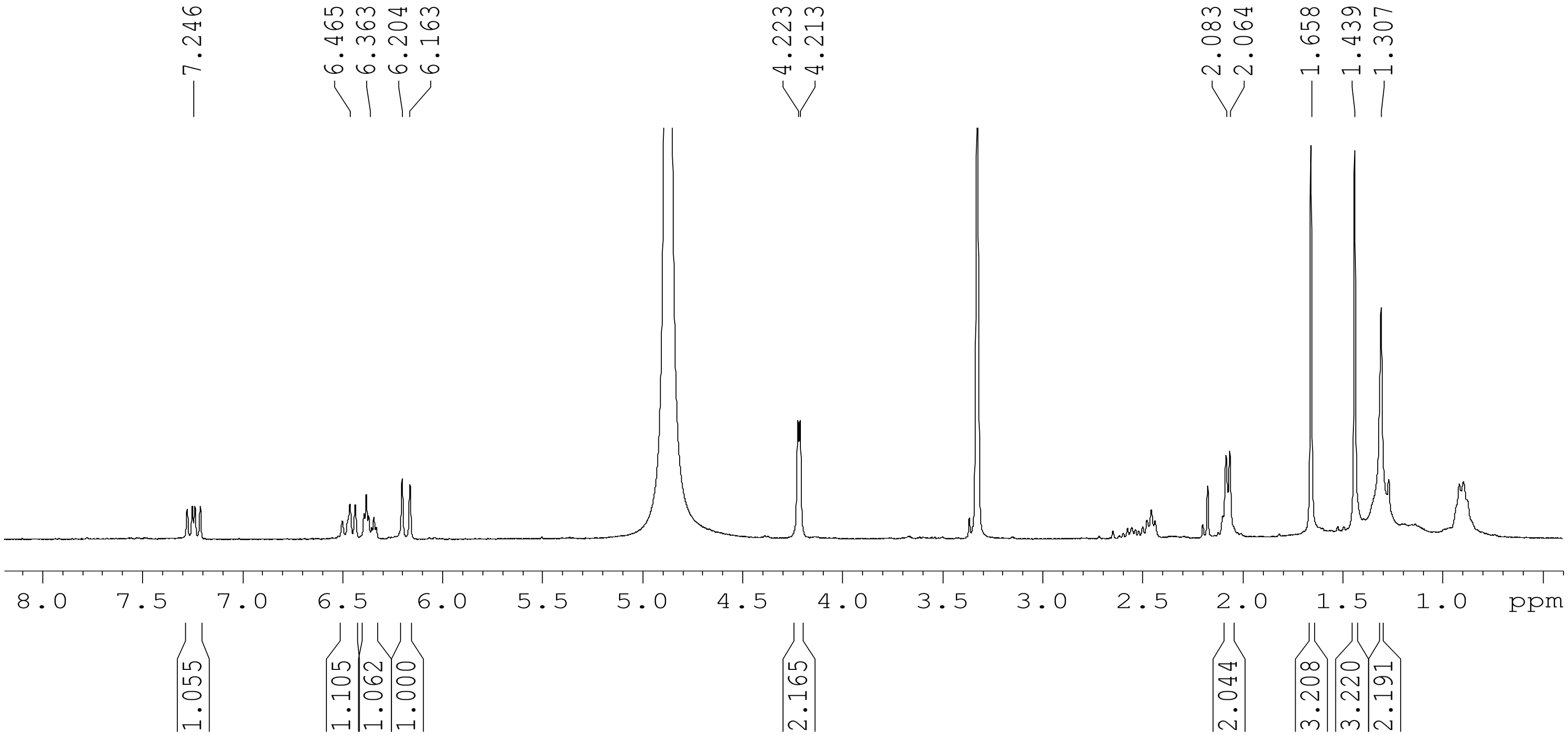

Figure 43. The ^{1}H NMR Spectrum of Compound **6** in CD_3OD (400 MHz)

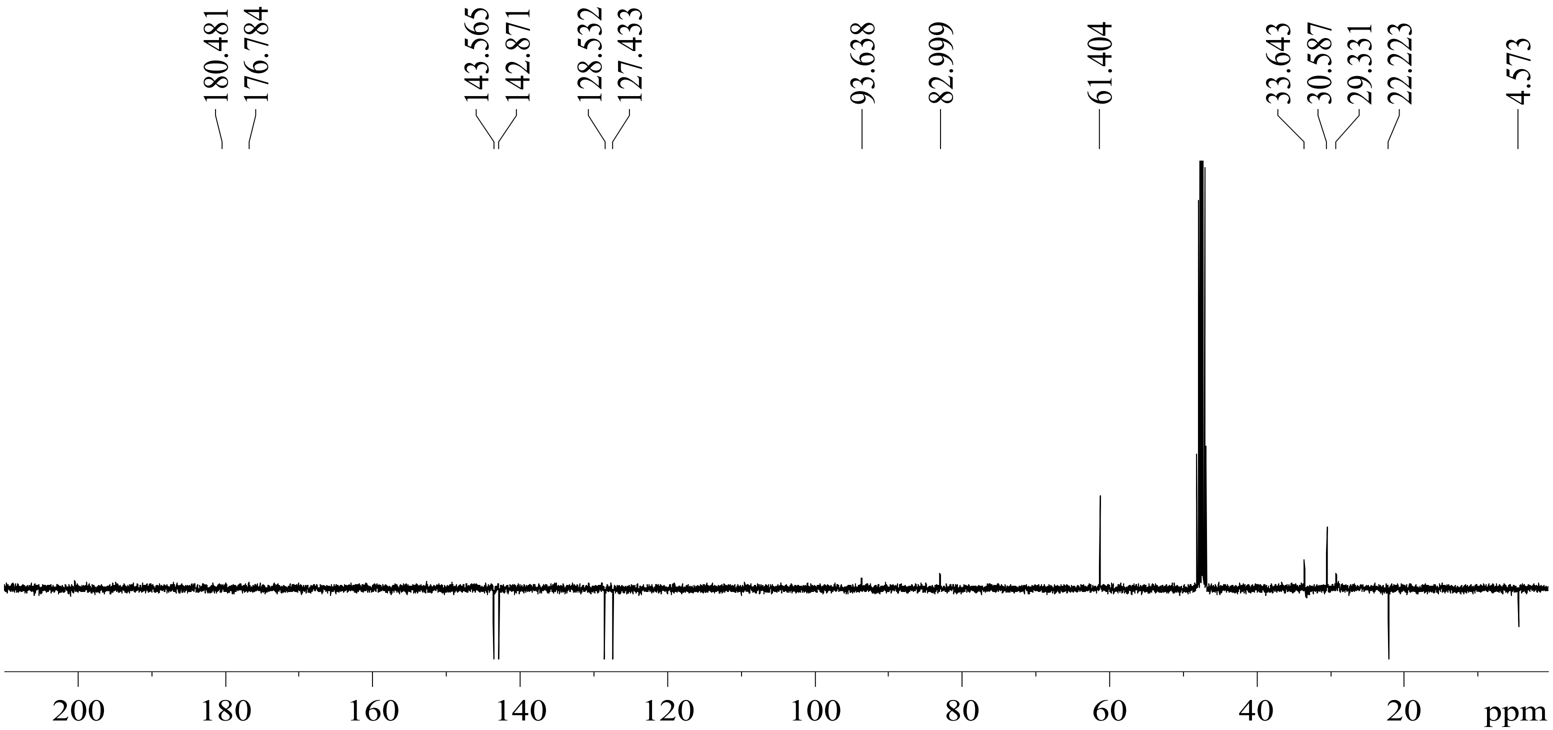

Figure 44. The ^{13}C APT NMR Spectrum of Compound **6** in CD_3OD (100 MHz)

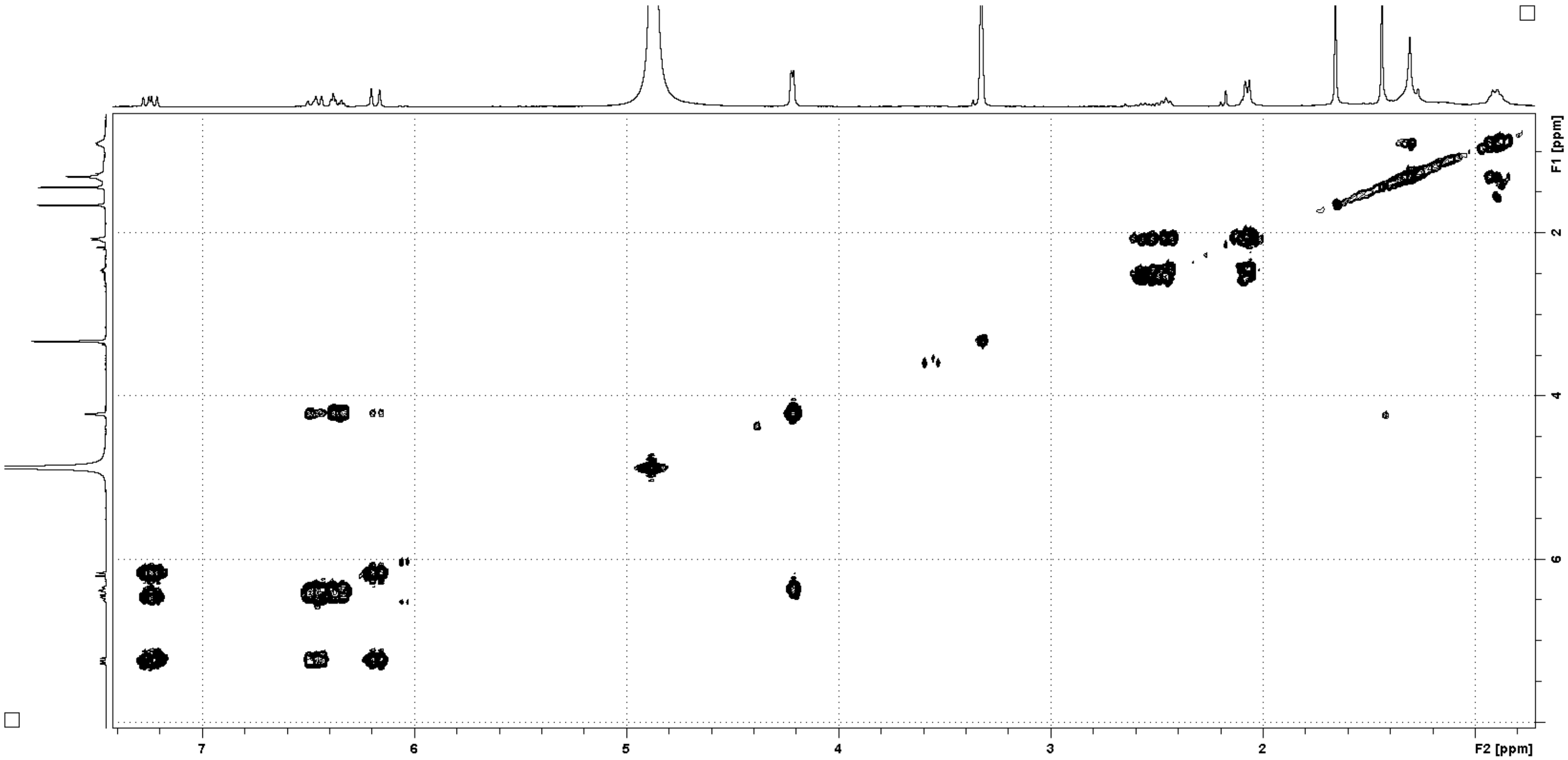

Figure 45. The ^{1}H-^{1}H COSY Spectrum of Compound **6** in CD_3OD (400 MHz)

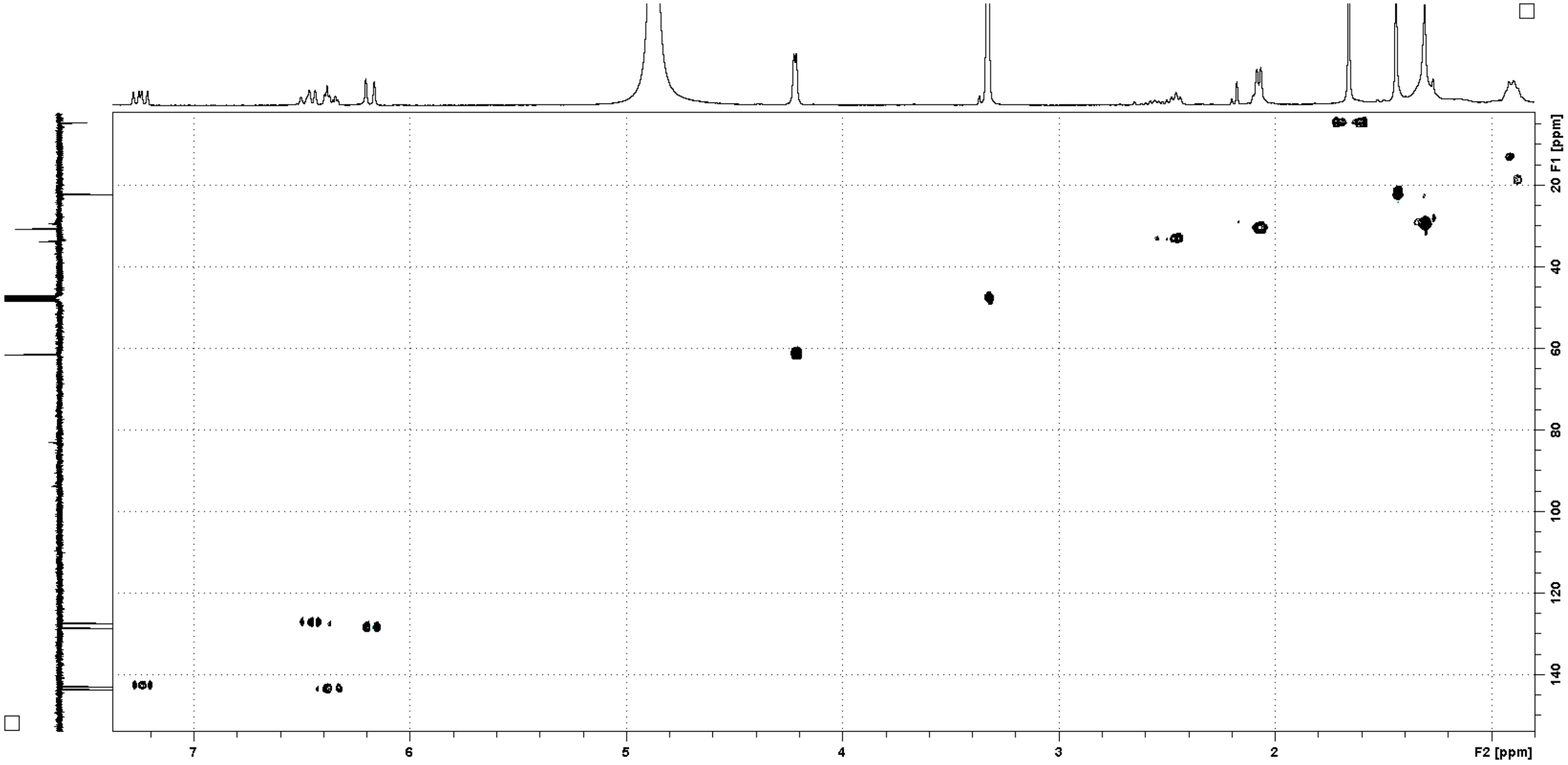

Figure 46. The HSQC Spectrum of Compound **6** in CD_3OD (400 MHz for ^1H)

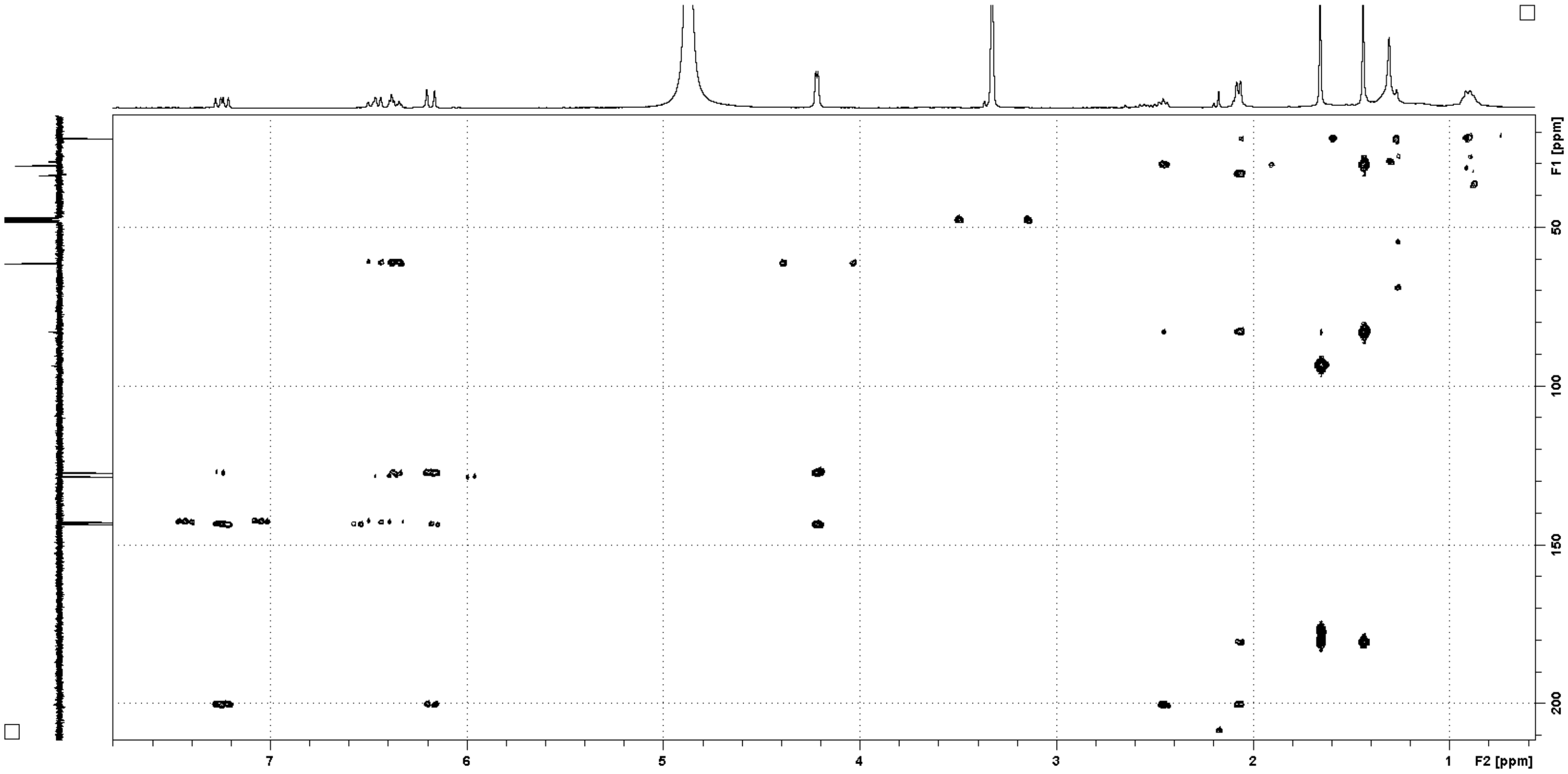

Figure 47. The HMBC Spectrum of Compound **6** in CD_3OD (400 MHz for ^1H)

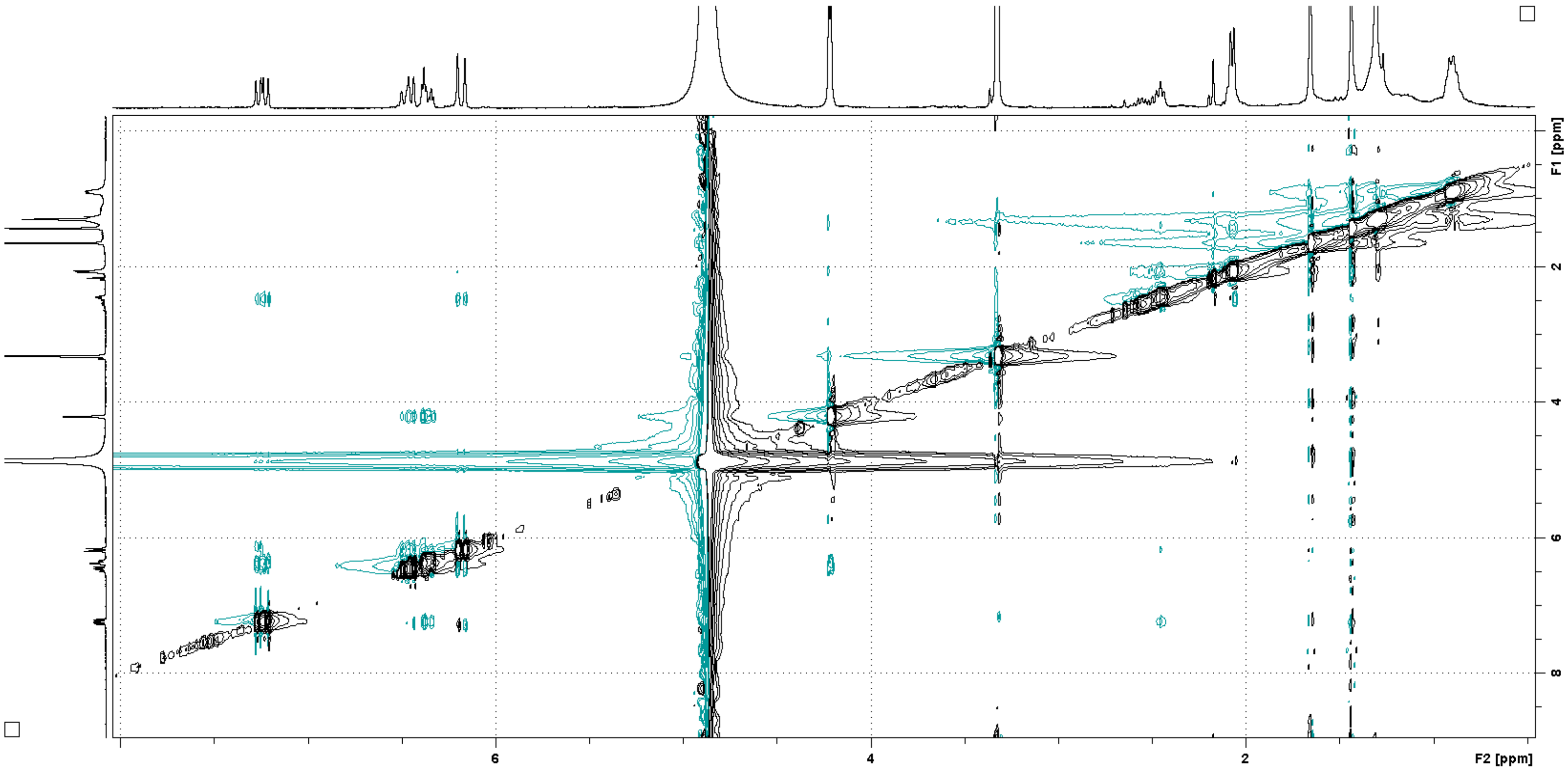

Figure 48. The ROESY Spectrum of Compound **6** in CD_3OD (400 MHz)

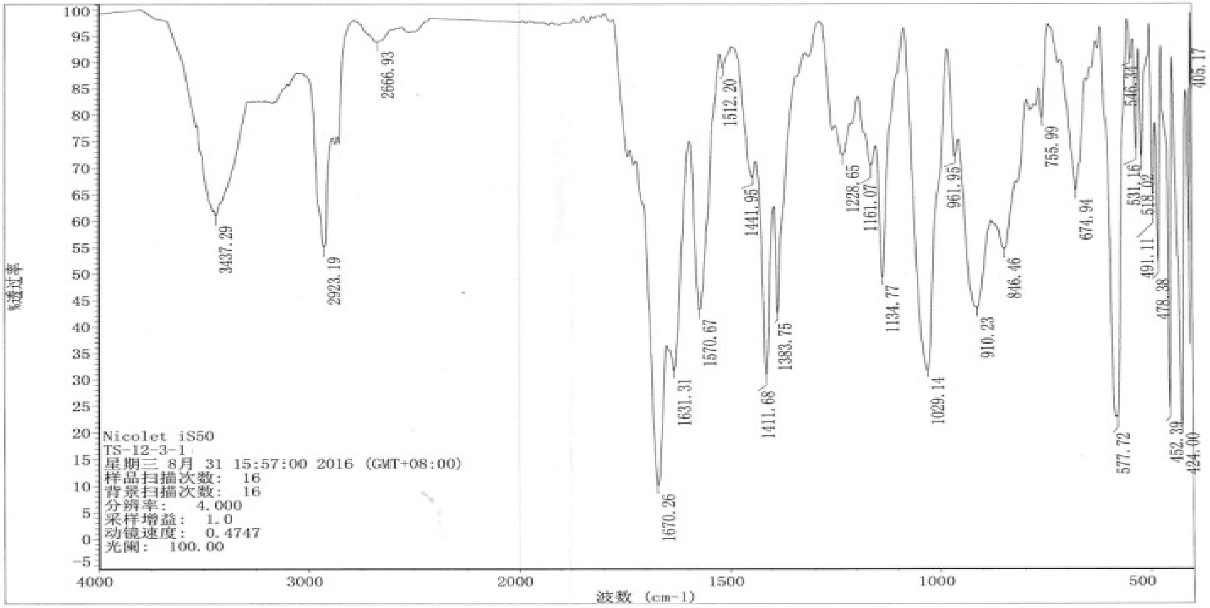

Figure 49. The IR Spectrum of Compound **7**

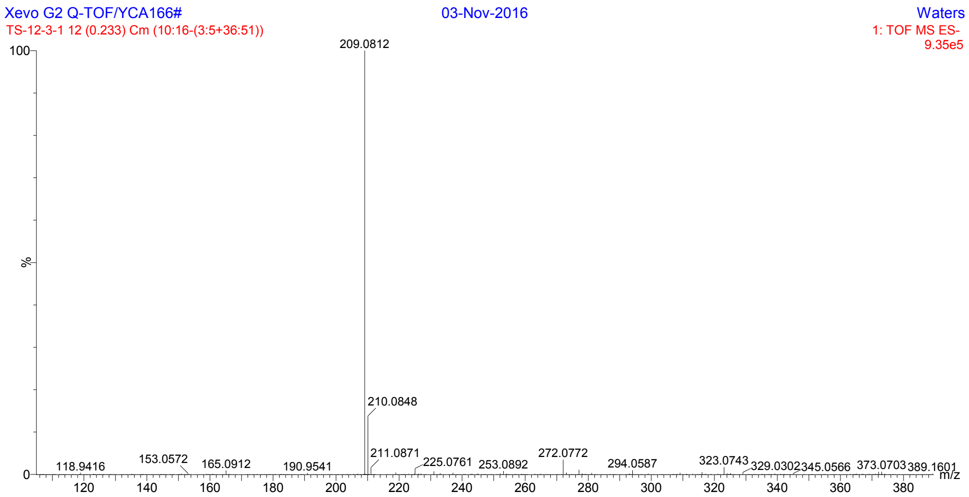

Figure 50. The HRESIMS Data of Compound **7**

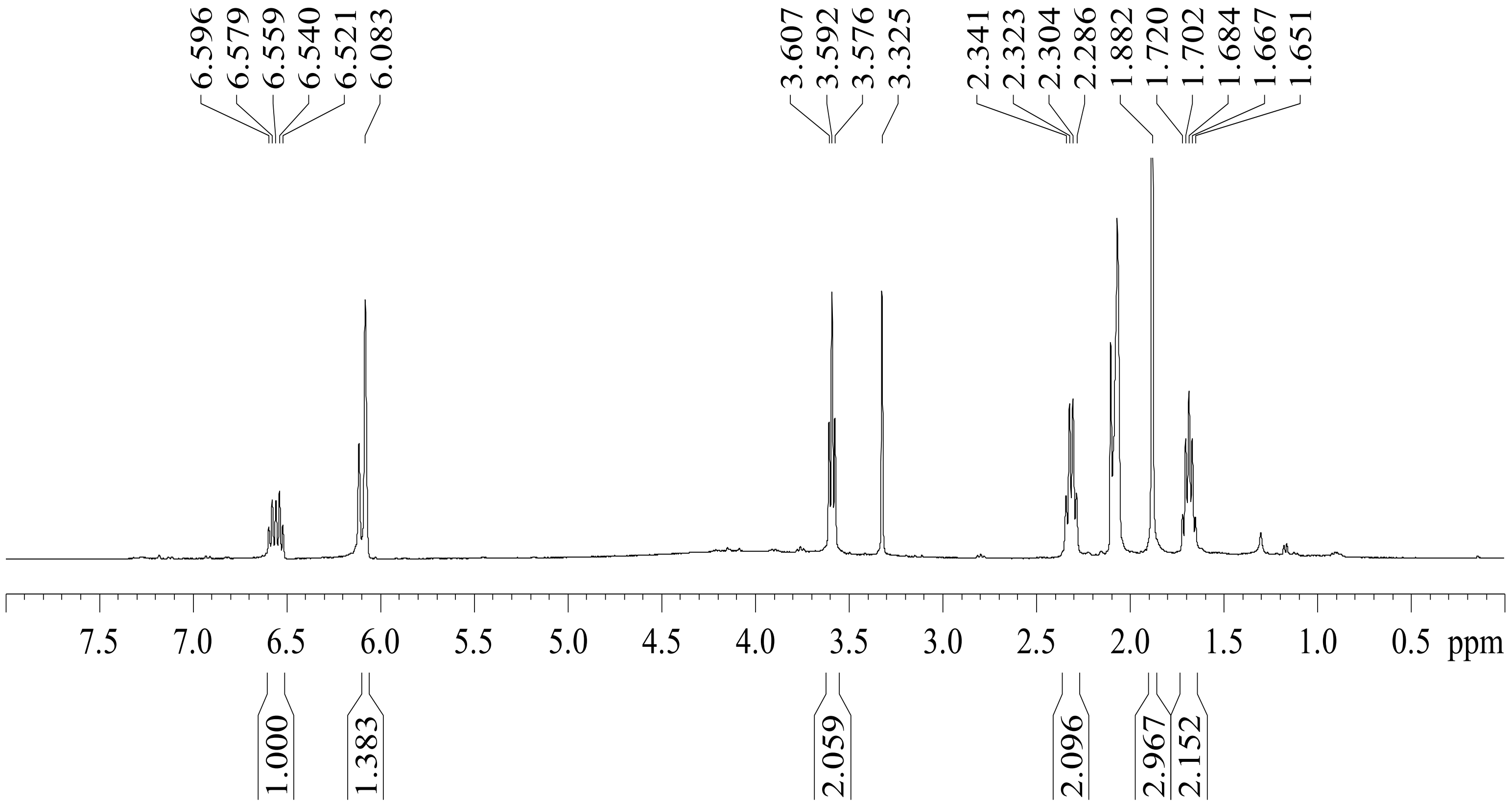

Figure 51. The ^{1}H NMR Spectrum of Compound **7** in Acetone-d_6 (400 MHz)

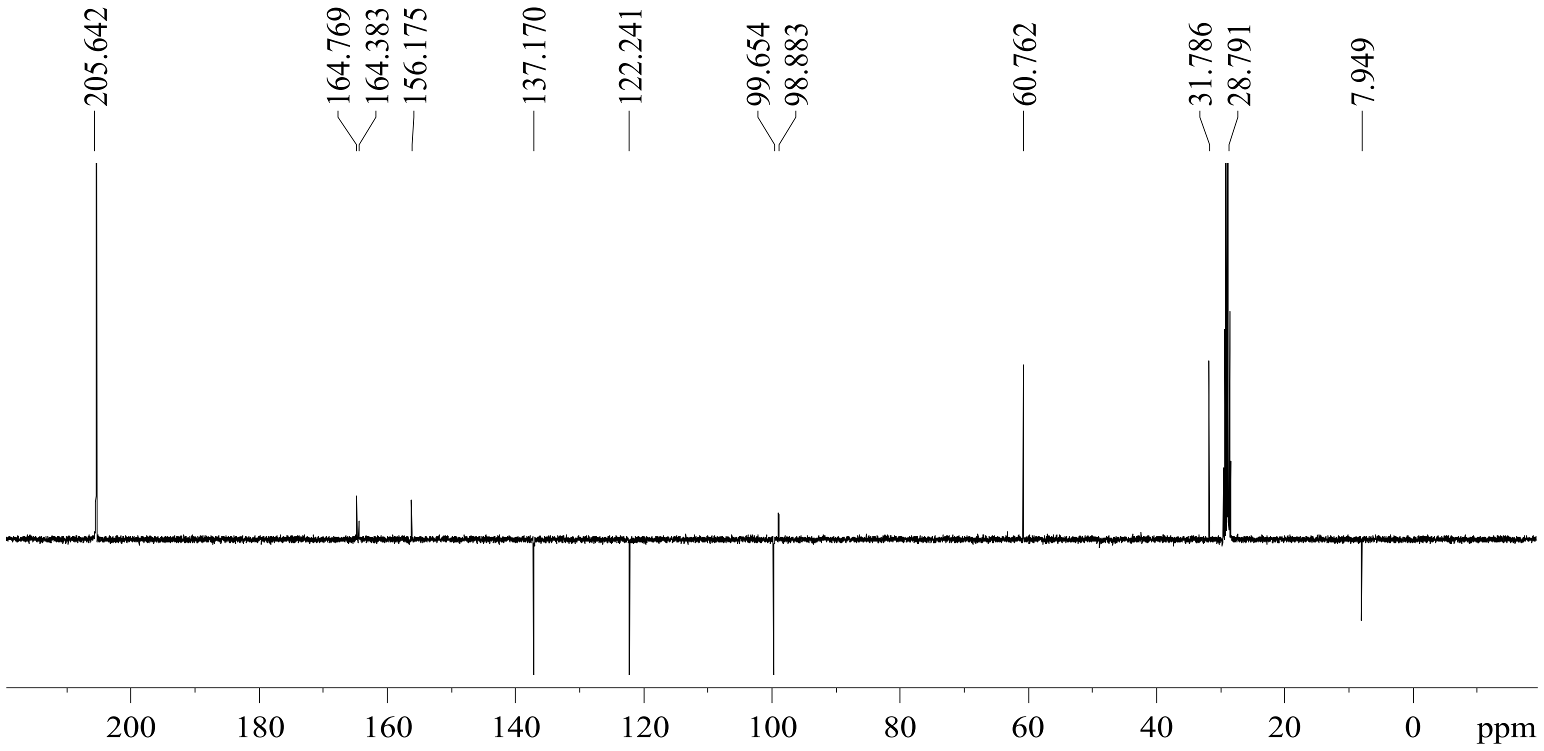

Figure 52. The ^{13}C APT NMR Spectrum of Compound **7** in Acetone-d_6 (100 MHz)

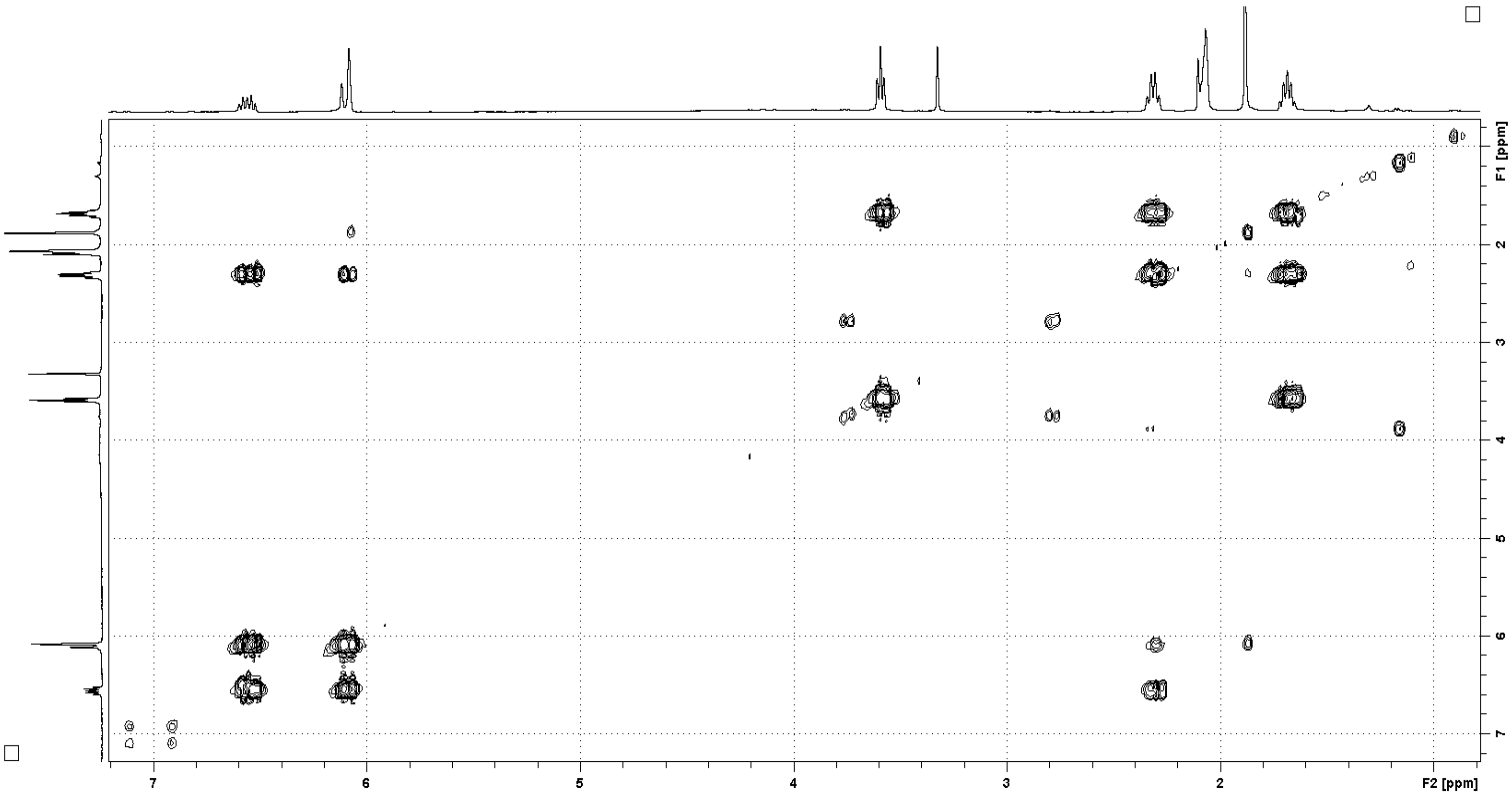

Figure 53. The ^{1}H-^{1}H COSY Spectrum of Compound **7** in Acetone-d_6 (400 MHz)

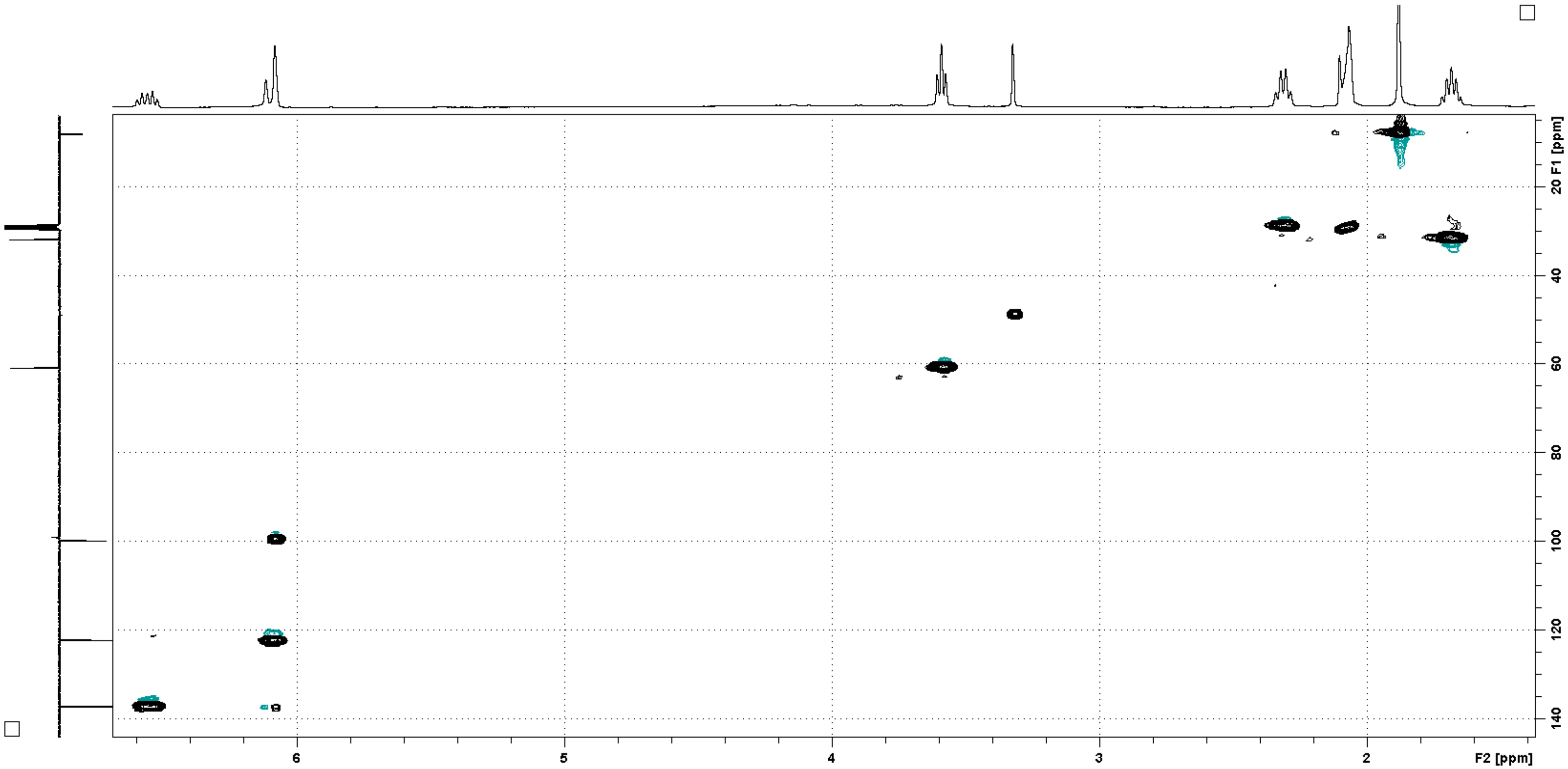

Figure 54. The HSQC Spectrum of Compound **7** in Acetone-d_6 (400 MHz for ^1H)

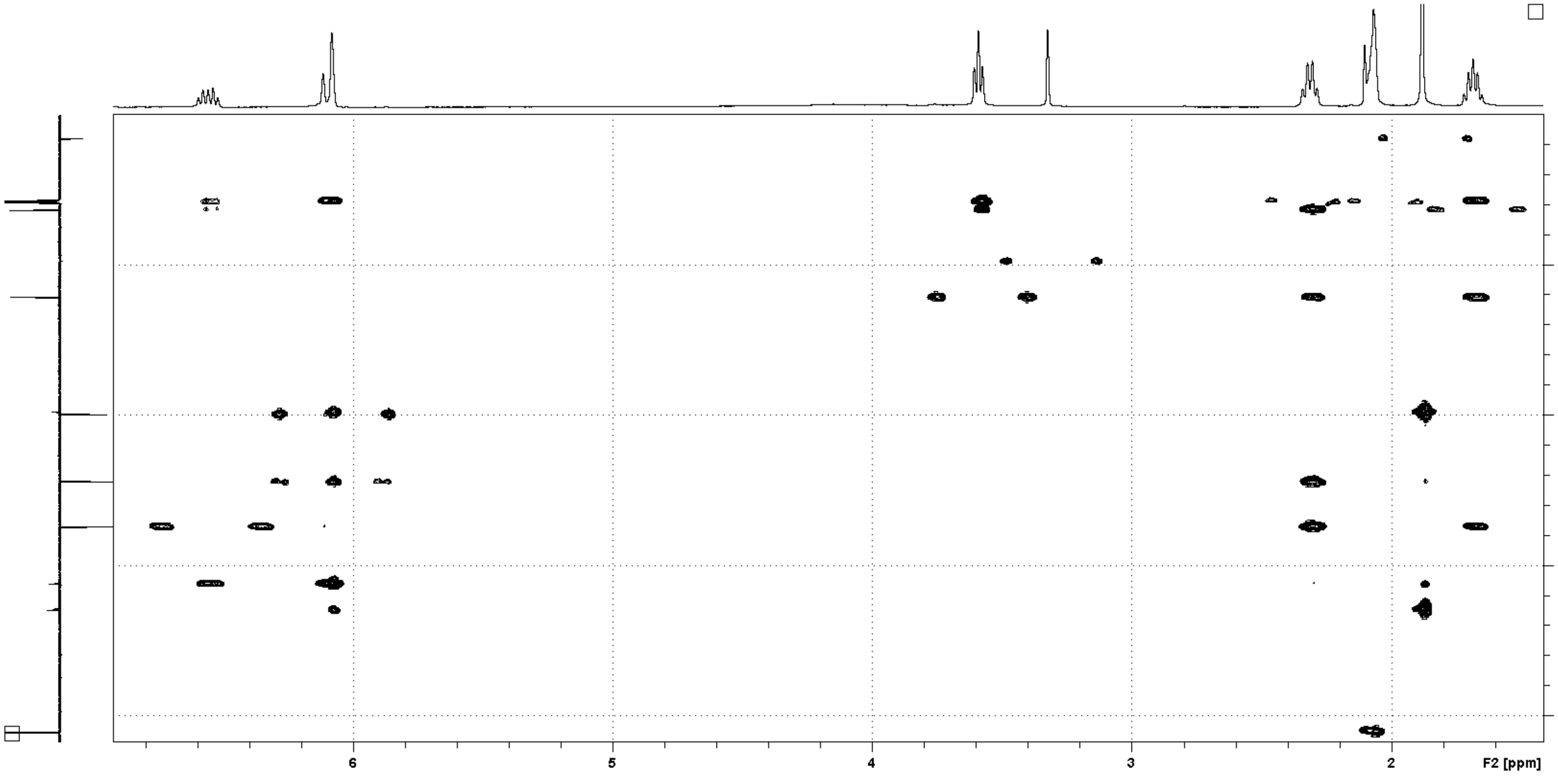

Figure 55. The HMBC Spectrum of Compound **7** in Acetone-d_6 (400 MHz for ^{1}H)

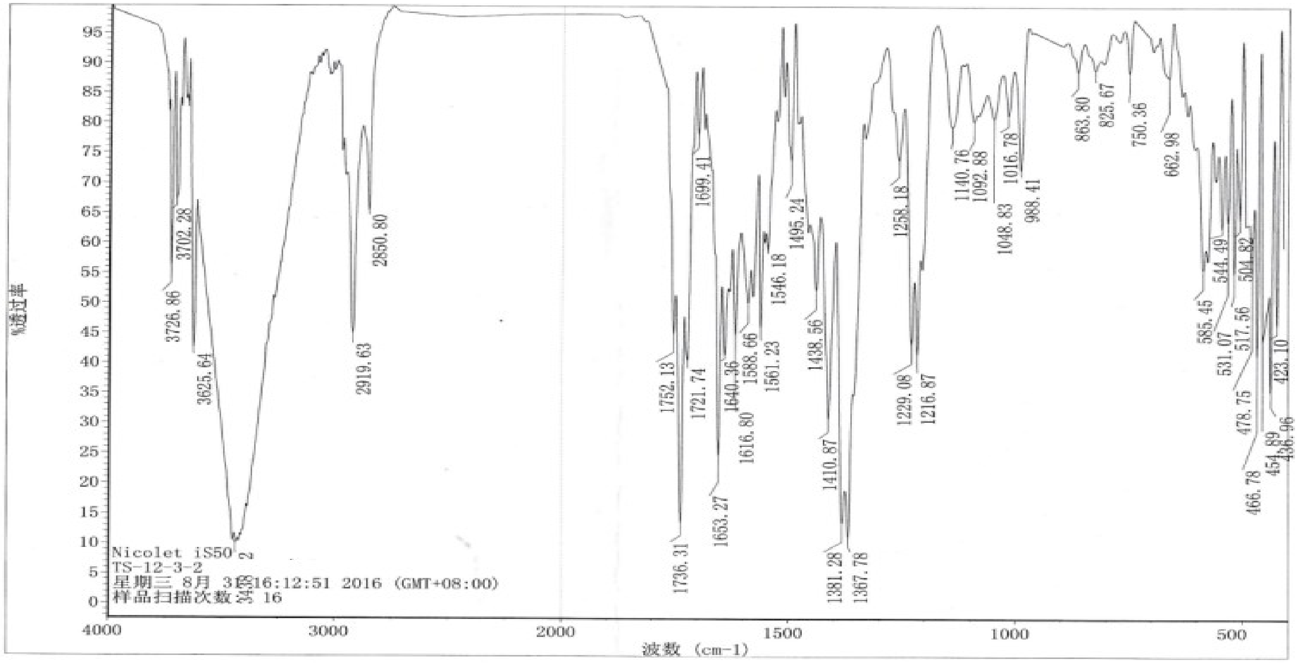

Figure 56. The IR Spectrum of Compound **8**

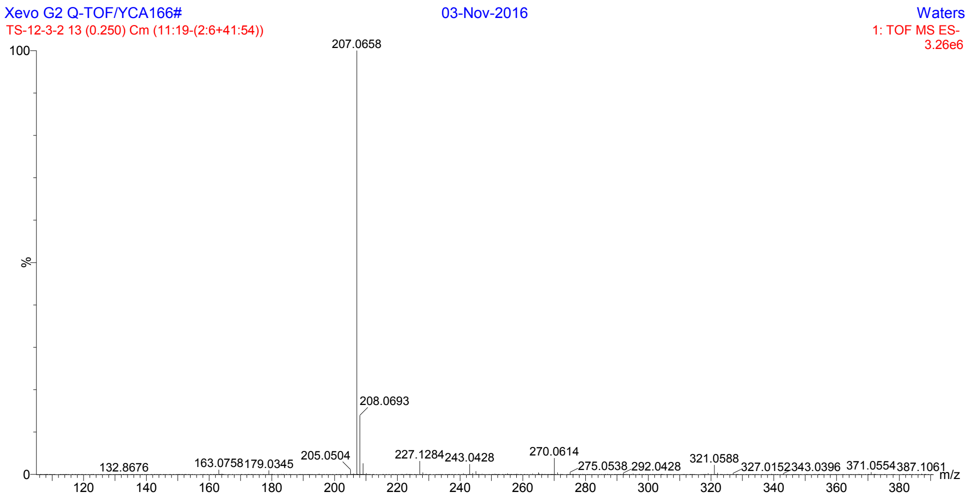

Figure 57. The HRESIMS Data of Compound **8**

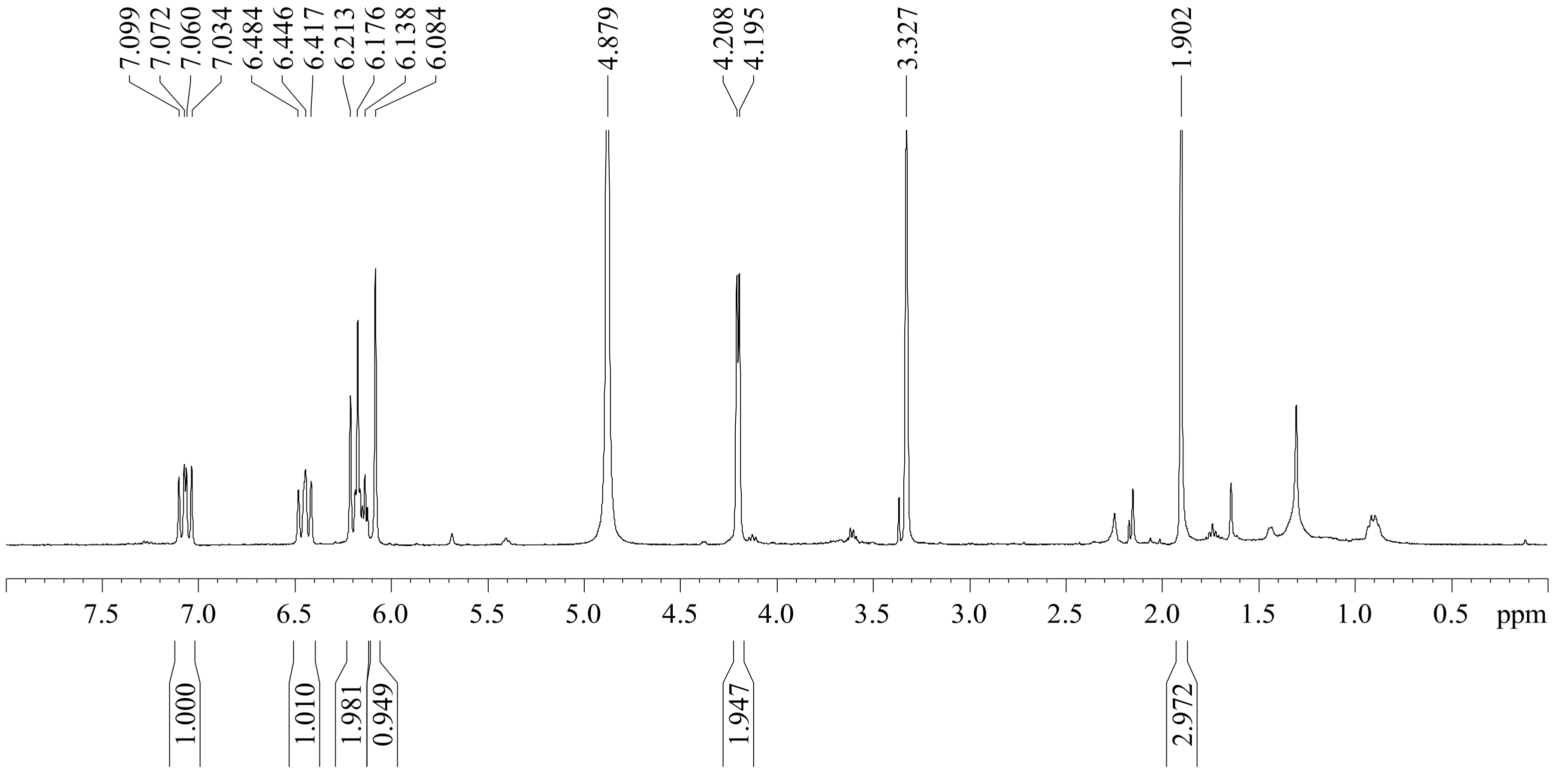

Figure 58. The ^{1}H NMR Spectrum of Compound **8** in CD_3OD (400 MHz)

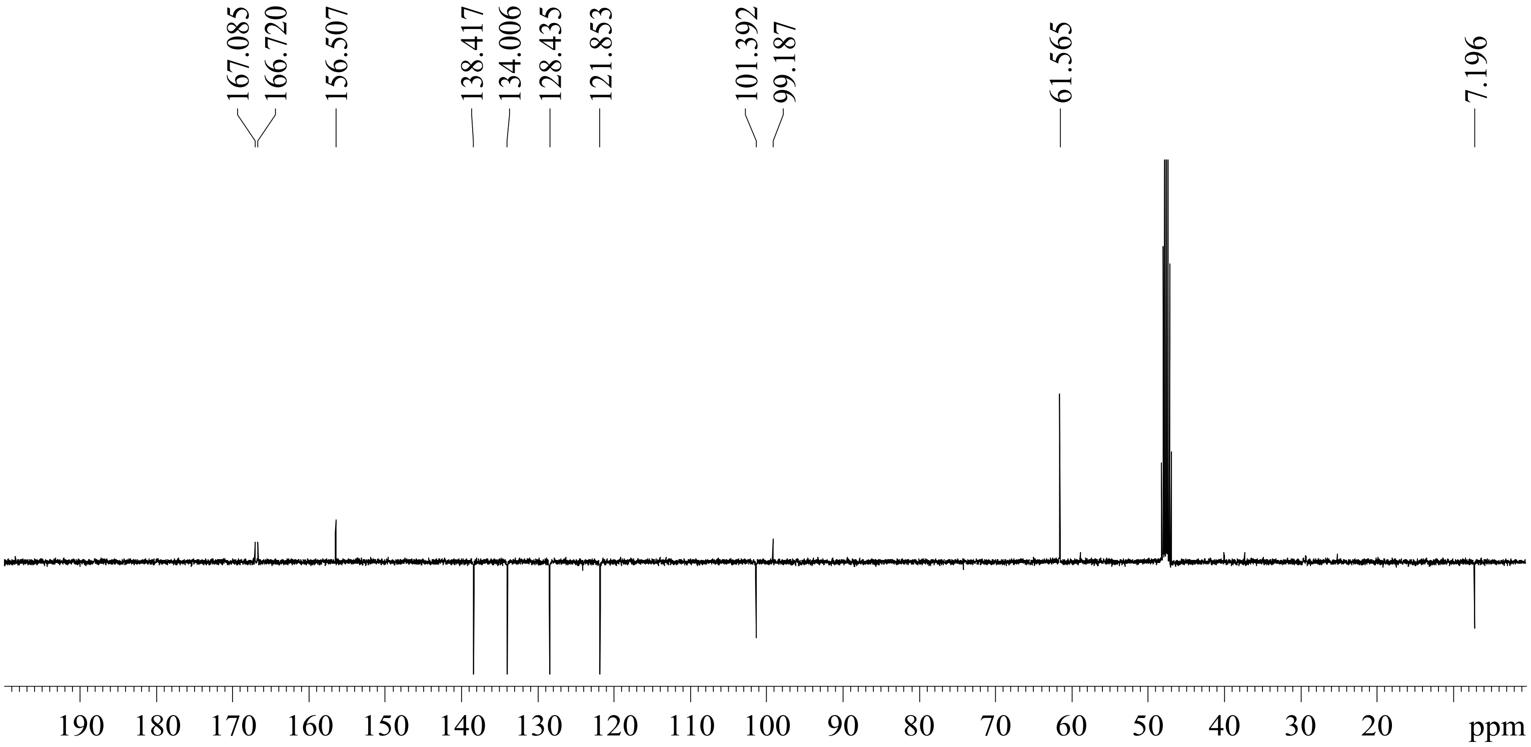

Figure 59. The ^{13}C APT NMR Spectrum of Compound **8** in CD_3OD (100 MHz)

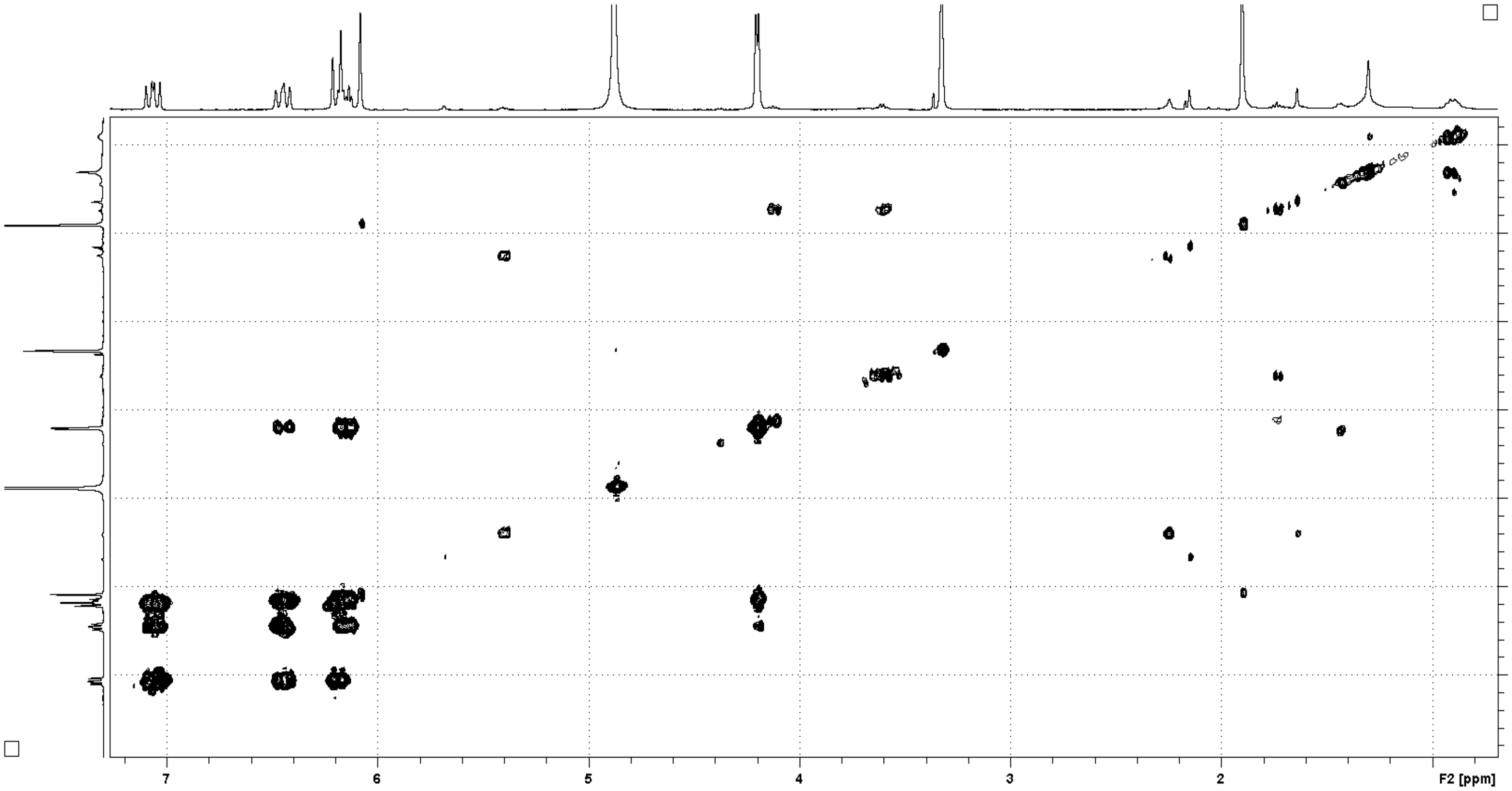

Figure 60. The ^{1}H-^{1}H COSY Spectrum of Compound **8** in CD_3OD (400 MHz)

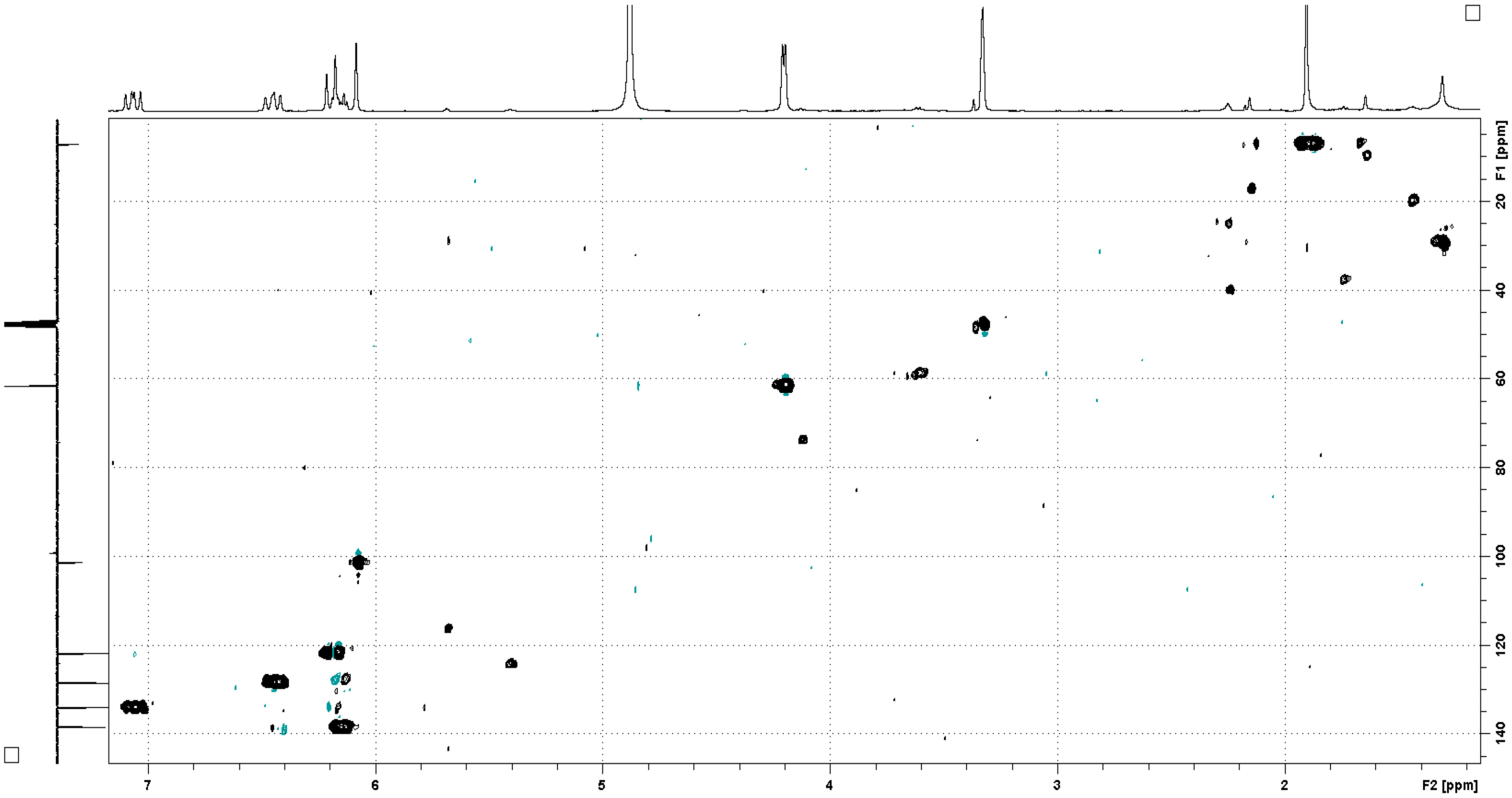

Figure 61. The HSQC Spectrum of Compound **8** in CD_3OD (400 MHz for ^1H)

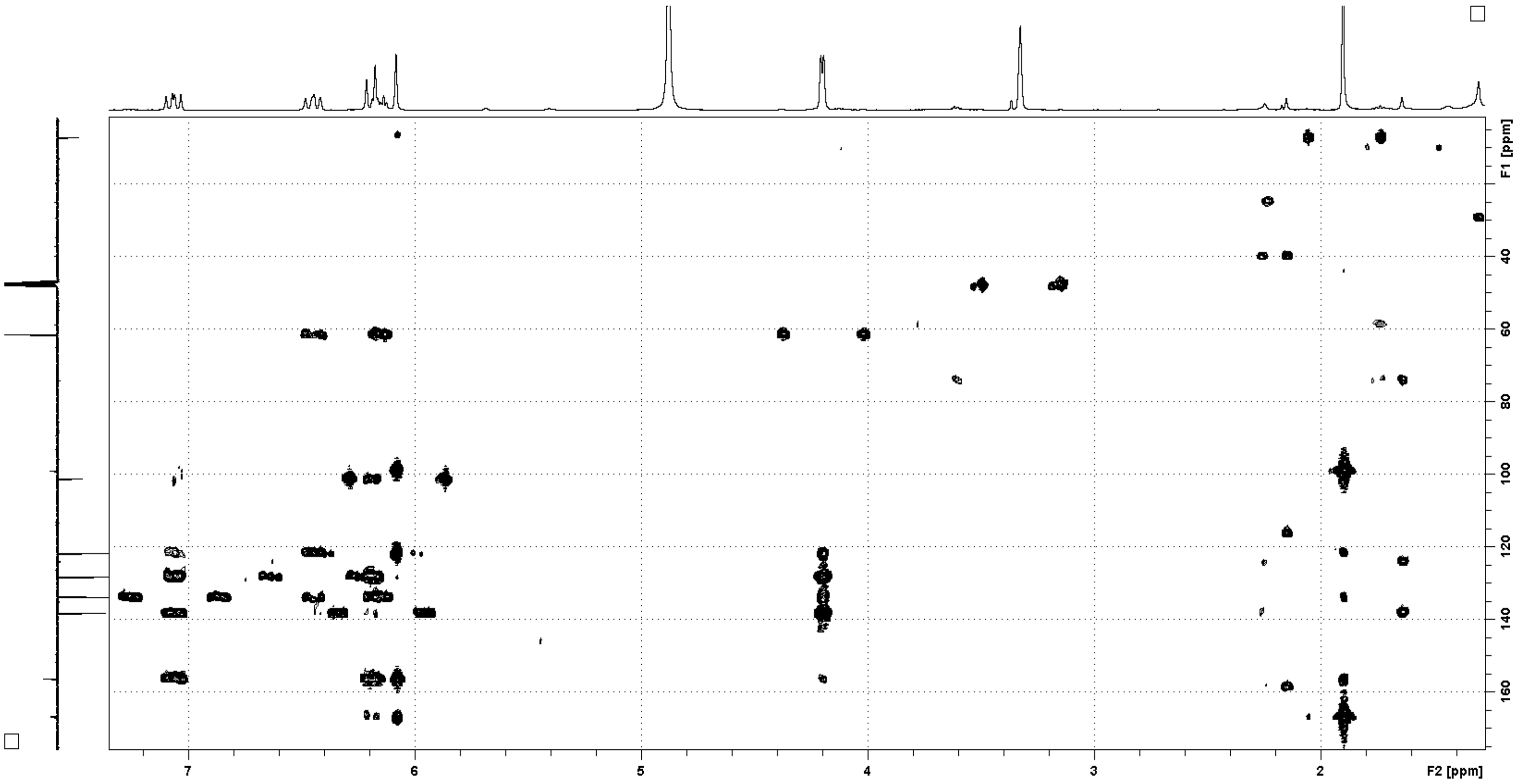

Figure 61. The HMBC Spectrum of Compound **8** in CD_3OD (400 MHz for ^1H)
